# Mechanically Planar-to-Point Chirality Transmission in [2]Rotaxanes

**DOI:** 10.1021/jacs.3c11611

**Published:** 2024-01-24

**Authors:** Julio Puigcerver, Marta Marin-Luna, Javier Iglesias-Sigüenza, Mateo Alajarin, Alberto Martinez-Cuezva, Jose Berna

**Affiliations:** † Departamento de Quimica Organica, Facultad de Quimica, Regional Campus of International Excellence “Campus Mare Nostrum”, Universidad de Murcia, E-30100 Murcia, Spain; ‡ Departamento de Quimica Organica and Centro de Innovacion en Quimica Avanzada (ORFEO-CINQA), Universidad de Sevilla, E-41012 Sevilla, Spain

## Abstract

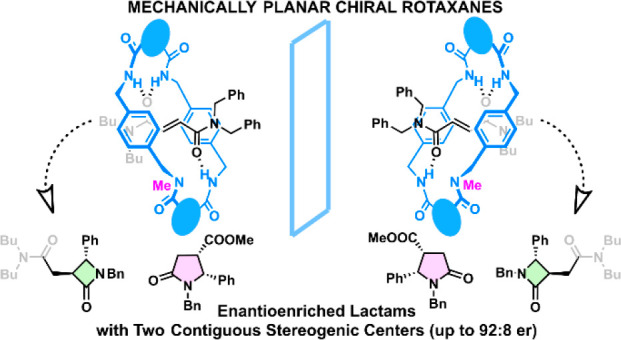

Herein we describe an effective transmission of chirality, from mechanically planar chirality to point chirality, in hydrogen-bonded [2]rotaxanes. A highly selective mono-N-methylation of one (out of four) amide N atom at the macrocyclic counterpart of starting achiral rotaxanes generates mechanically planar chirality. Followed by chiral resolution, both enantiomers were subjected to a base-promoted intramolecular cyclization, where their interlocked threads were transformed into new lactam moieties. As a matter of fact, the mechanically planar chiral information was effectively transferred to the resulting stereocenters (covalent chirality) of the newly formed heterocycles. Upon removing the entwined macrocycle, the final lactams were obtained with high enantiopurity.

The research concerning the synthesis of enantioenriched mechanically interlocked molecules (MIMs) is increasing during the past years,^1^ finding exciting applications in the fields of asymmetric catalysis^2^ or sensing of chiral molecules,^3^ among others.^4^ The inherent architecture of MIMs,^5^ with at least two entwined components, increases the possibilities for the introduction of chiral information. In rotaxanes, the incorporation of point^6^ or axial stereogenic elements,^7^ on the thread or the macrocycle, are the most employed strategies. In addition, MIMs can exhibit types of stereochemistry that rely only on the mechanical bond as a consequence of its interlocked architecture when all their subcomponents are achiral, such as the so-called mechanically planar chirality (MPC)^1a,8^ or, as recently reported, mechanically axial chirality.^9^ Different strategies are followed for the obtention of enantioenriched MPC-rotaxanes, including the resolution of the corresponding racemates by chiral stationary-phase high-performance liquid chromatography (CSP-HPLC),^10^ direct asymmetric syntheses,^11^ desymmetrization of achiral rotaxanes,^12^ kinetic resolutions,^13^ or the use of chiral auxiliaries.^14^ To date, the number of applications for these MPC-rotaxanes is scarce. Takata and co-workers employed these type of systems as chiral inductors for the assembly of helicenes,^15^ whereas the Goldup group synthesized an enantiopure rotaxane with mechanically planar chirality and successfully used it as ligand in gold(I)-catalyzed transformations.^16^ In addition, examples in the use of MPC-rotaxanes in chiral sensing have been also disclosed.^17^ In this line, we have recently reported the synthesis of enantioenriched lactams and β-amino acids,^18^ using a diastereomeric mixture of rotaxanes with covalent/mechanical chirality (mechanoisomers) as reactants. The chiral information was effectively transmitted to the stereocenters of the new lactam core at the thread. However, the stereocontrol was primarily dictated by the covalent stereochemistry, with the mechanical stereochemistry playing a small role on the reaction.

We herein present a strategy for an effective transmission of the chiral information in [2]rotaxanes from mechanically planar chirality to point chirality. For this goal, the intramolecular cyclization of enantiopure MPC-interlocked fumaramides **1**, obtained after desymmetrization of achiral rotaxanes and posterior chiral resolution, was selected as a promising transformation (Scheme 1),^19^ aiming to access to enantioenriched lactams **2** and **3**, both with new stereogenic centers.

**Scheme 1:**
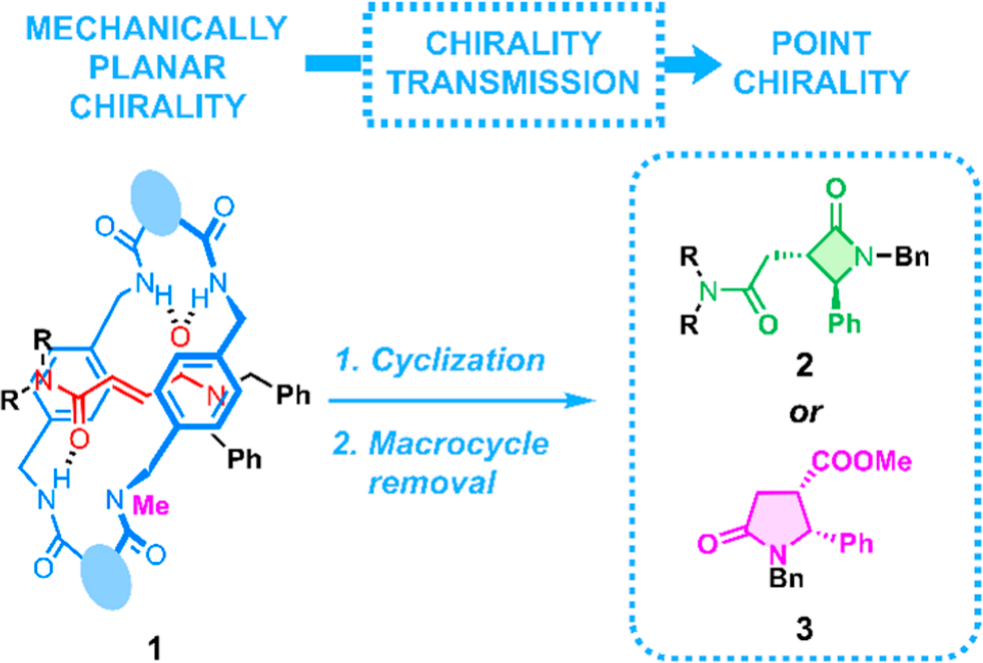
Transmission of Chirality from MPC-rotaxanes to Point Chiral Lactams Our approach: (1) intramolecular cyclization; (2) removal of the macrocycle for liberating the enantioenriched lactams.

To validate our hypothesis, we synthesized the kinetically stable pseudo[2]rotaxane **4a** (Scheme 2),^19a^ with the nonsymmetrical thread *N*,*N*′,*N*′,*N*-dibenzyldibutylfumaramide **T1** (see Supporting Information for synthetic details). The flexible *n*-butyl groups in **T1** were designed to facilitate the subsequent removal of the macrocycle under thermal conditions.^20^ The selective mono-N-methylation of one out of four identical NH amide groups of the entwined macrocycle is a highly challenging task. The reaction of **4a** with NaH in THF at room temperature and methyl iodide as the methylating reagent exclusively yielded the tetramethylated rotaxane **6a**, not detecting the foreseen monomethylated **1a** (Scheme 2).^21^ To overcome this undesired scenario, we synthesized rotaxane **4b**, comprising thread **T1** and an entwined macrocycle with two types of NH amide groups with different acidities (see Scheme S4 for p*K*_a_ calculations).^22^ The reaction of **4b** with NaH and MeI afforded two products, identified as the monomethylated and dimethylated pseudo[2]rotaxanes **1b** and **5b**, respectively, displaying a promising selectivity (57%) toward the desired **1b** (see Table S1). After optimizing the reaction conditions (Table S1 and Figures S1 and S2), we increased the selectivity up to 97% by using CsOH as the base at 0 °C, nearly avoiding the formation of the dimethylated byproduct **5b**, not observing other di-, tri- or tetramethylated derivatives of **4b**. We also explored rotaxane **4c**, featuring smaller *n*-propyl groups as stoppers, exhibiting similar reactivity to **4b**, although the monomethylated **1c** was unstable toward dethreading (Scheme 2 and Table S1). To circumvent chiral resolutions, we attempted the direct enantioselective desymmetrization of rotaxane **4b** through either the use of chiral lithium amides as bases or the employing of chiral methylating reagents (Schemes S1 and S2). Unfortunately, both attempts resulted in the isolation of racemic rotaxane *rac*-**1b**.

**Scheme 2:**
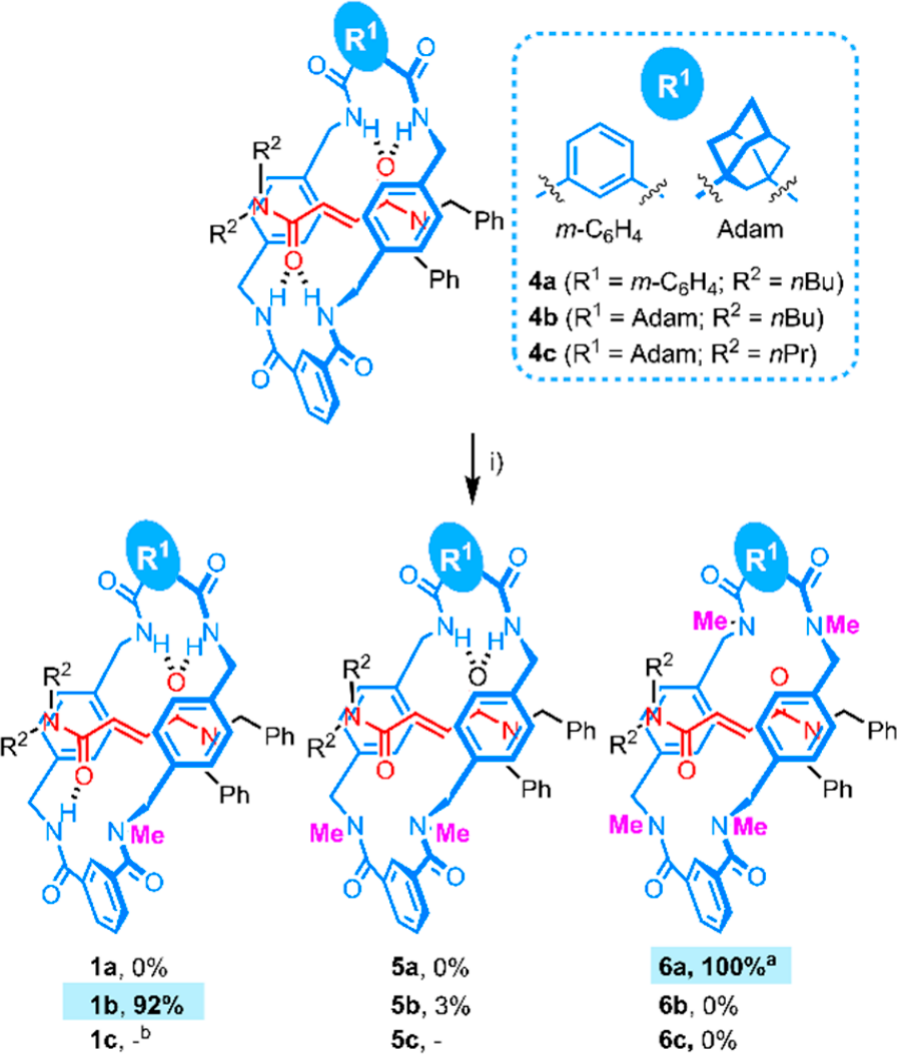
N-Methylation of Rotaxanes **4** for the Formation of *rac*-**1** (*S*_*mp*_ Enantiomers of **1** Are Shown) Reaction carried out with NaH at 25 °C. **1c** was unstable towards the competitive dethreading reaction. Reaction conditions: (i) (1) **1** (10 mg, 1 equiv), THF (1 mL), CsOH (5 equiv), 0 °C, 30 min; (2) MeI (10 equiv), 0 °C, 24 h.

At this point, it should be noted that the methylation of one amide group in **4b** not only induced the emergence of mechanically planar chirality but also led to the desymmetrization of its adamantane core, arising two new stereogenic centers (indicated in Figure 1c). However, this point chirality is fixed once the mechanically planar chirality arises due to the configuration of all stereogenic units being intrinsically linked. Both species were separated by preparative CSP-HPLC (Figure 1a) and their corresponding CD spectra recorded (Figure 1b). We successfully crystallized the *enantiomer 2* (eluted at a retention time of 21.8 min) of **1b**, and its absolute configuration was determined, resulting in **(*R*_*mp*_, 1**^**5**^***S,*1**^**7**^***R*)-1b** according to the CIP rules (Figure 1c, inset, and Scheme S6).^8^ The *N*-methyl group is attached, as expected, to one of the isophthalamide N atoms, highly distorting the tetraamide macrocycle. Moreover, one of the two aromatic walls of the *p*-xylylendiamine fragments is perpendicular to the fumaramide double bond instead of both adopting the habitual sandwich conformation surrounding the fumaramide C=C bond. The distortion of the macrocycle generates a cavity with shorter dimensions,^23^ a plausible reason behind the high selectivity observed in the methylation reaction toward **1b**. In addition, the structure of the nonchiral dimethylated pseudorotaxane **5b** was also elucidated by SCXRD, having both methyl groups linked at the N atoms of the isophthalamide unit (Figure 1d).

**Figure 1:**
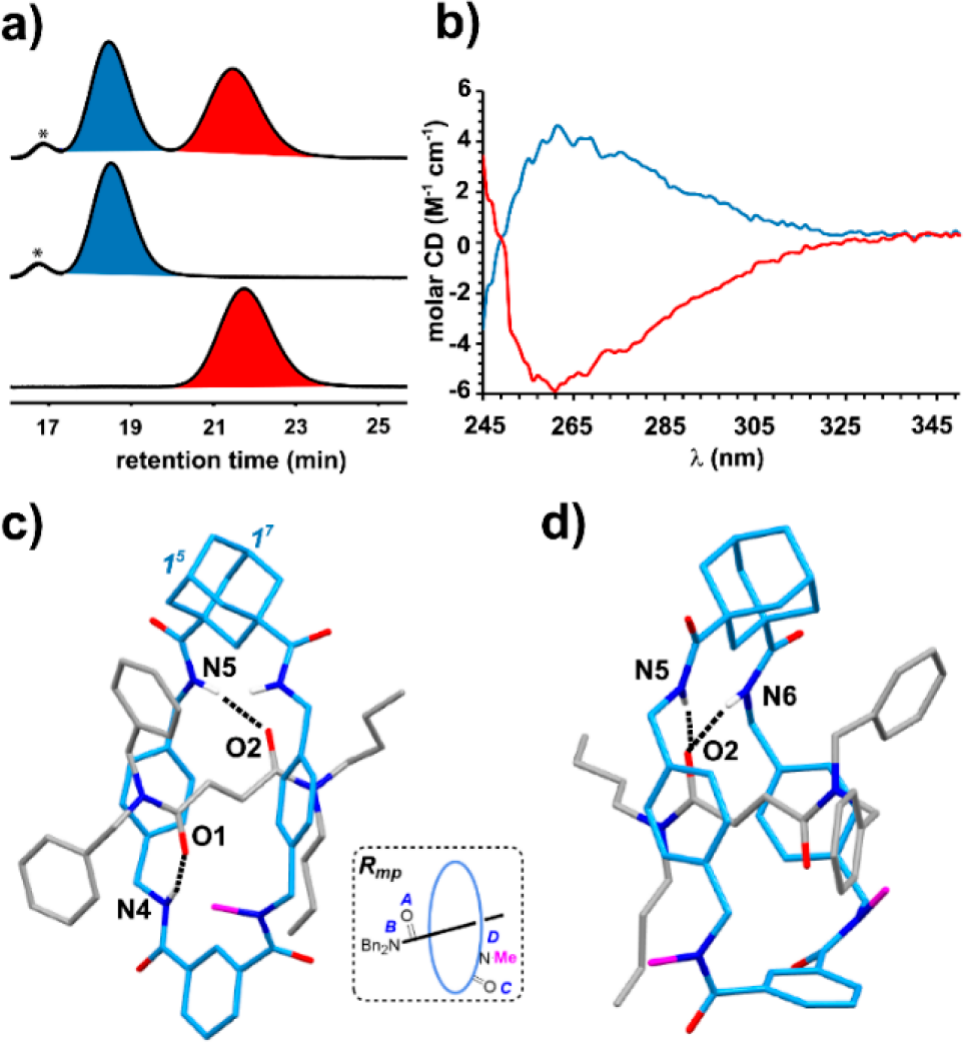
(a) HPLC chromatograms of *rac*-**1b** (top), first enantiomer of **1b** (middle), and second enantiomer of **1b** (bottom) (Chiralpak IC-3 column, 85:13:2 CH_2_Cl_2_:MeCN:iPrOH, 0.5 mL·min^–1^, 254 nm) (*rotaxane **5b**). (b) CD spectra of the first (blue line, 1.0 × 10^–5^ M in CHCl_3_) and second enantiomer of **1b** (red line, 1.2 × 10^–5^ M in CHCl_3_). (c) X-ray structure of **(*R*_*mp*_, 1**^**5**^***S,*1**^**7**^***R*)-1b**. Intramolecular hydrogen-bond lengths (Å) (angles [deg]): N4–H04···O1 2.06 (166); N5–H05···O2 2.07 (161). (d) X-ray structure of **5b**. Intramolecular hydrogen-bond lengths [Å] (angles [deg]): N5–H05···O2 2.16 (166); N6–H06···O2 2.25 (167). Inset: Absolute configuration assignment for **(*R*_*mp*_, 1**^**5**^***S,*1**^**7**^***R*)-1b**.

Having separated both enantiomers of the rotaxane **1b**, we carried out a base-promoted intramolecular cyclization of both interlocked benzylfumaramides (Scheme 3).^19^ The reaction of each enantiomer of **1b** could afford two diastereoisomers of the interlocked *trans*-β-lactam **7b** (plus another two of the corresponding *cis* isomers, never observed previously) (Scheme S3). We expected that the cyclization of enantiopure rotaxanes **1b** would occur diastereoselectively, majorly yielding one of the diastereoisomers of the interlocked *trans*-lactam **7b**. When we carried out the cyclization reaction of the **(*S*_*mp*_*,*1^5^*R,*1^7^*S*)-1b** (*enantiomer 1*) in the presence of CsOH (1 equiv) in DMF at room temperature, we observed a spot-to-spot transformation in less than 1 h. The ^1^H NMR spectrum of the reaction crude was difficult to analyze due to the broadening of the signals as a consequence of the entwined macrocycle, not being possible to determine the ratio of the different isomers of **7b**, which also could not be separated by HPLC (Figure S3). To simplify the data, we performed a dethreading reaction by heating the crude mixture at 180 °C in DMSO solution for 2 h under microwave irradiation (or under conventional heating for 2 days at 120 °C), affording free *trans*-β-lactam **2b**. Fortunately, and as we initially hypothesized, *trans*-β-lactam **2b** was enantiomerically enriched, with a promising 75:25 enantiomeric ratio (er). The decreasing of the temperature until —20 °C (3 equiv of base were needed to speed up the process, requiring 24 h) gave an improved 91:9 er after macrocycle removal, starting from both enantiomers of **1b** (Scheme 3). The structure of the major diastereoisomer of the interlocked lactam **7b**, obtained by cyclization of **(*R*_*mp*_*,*1^5^*S,*1^7^*R*)-1b**, was elucidated by SCXRD (Scheme 3, inset),^24^ confirming its absolute configuration as the **(*R*_*mp*_*,*1^5^*S,*1^7^*R,*3*R,*4*S*)-7b** stereoisomer. We also enantioselectively synthesized the corresponding *cis*-γ-lactam **3b**, by heating the interlocked lactams **7b** in the presence of diluted HCl and methanol,^18^ practically equaling the enantiopurity of β-lactams **2b** (92:8 er). Thanks to the SCXRD technique and the reported data of the enantioenriched lactams **2b** (HPLC chromatograms and specific rotations),^18^ the assignment of the absolute configuration of all compounds was unequivocally accomplished (Scheme S5 and Figure S9).

**Scheme 3:**
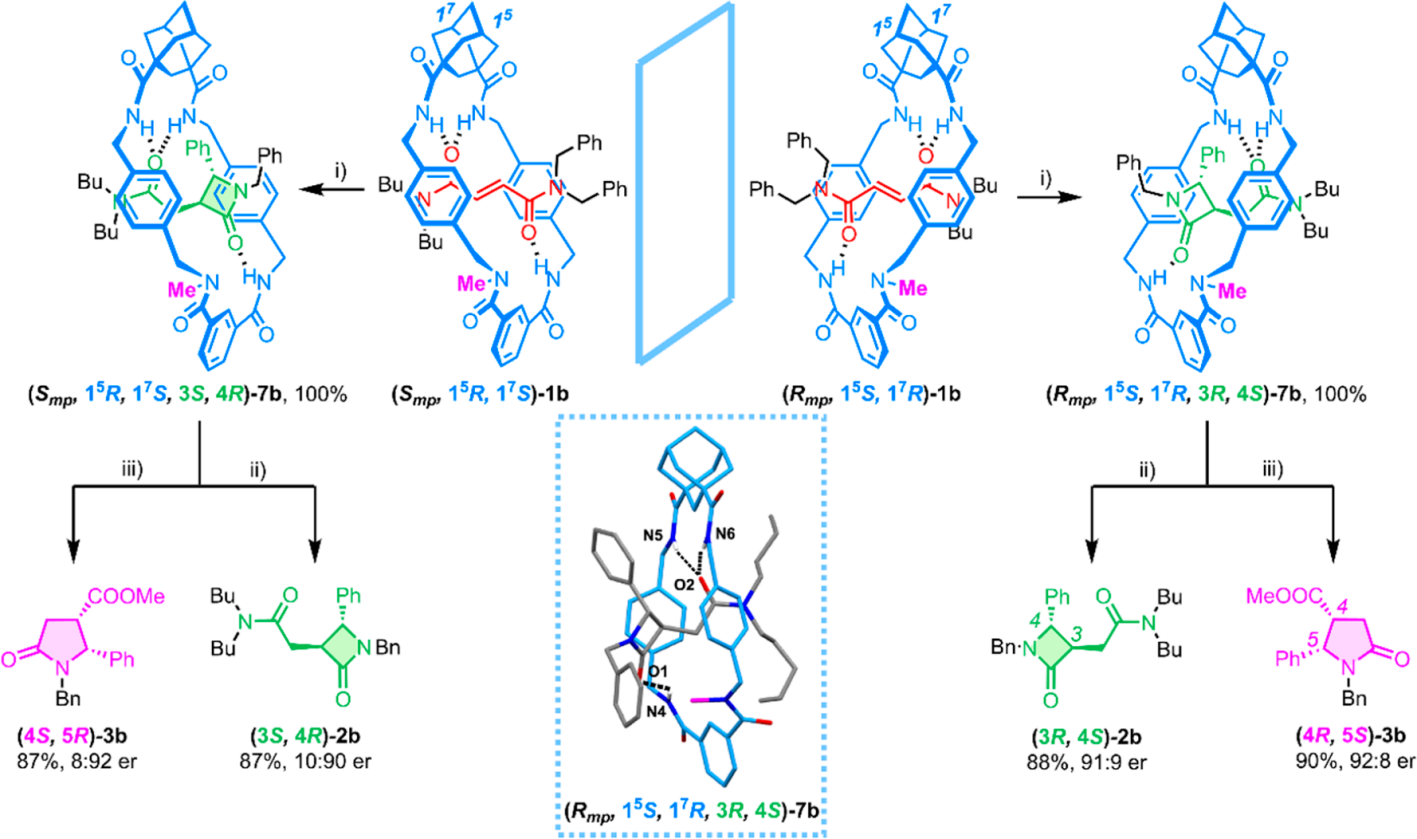
CsOH-Promoted Cyclization of Enantiomers of **1b** To Afford the Interlocked *trans*-Lactams **7b**, Followed by Dethreading for the Obtention of Enantioenriched *trans*-**2b** and *cis*-**3b** Reaction conditions: (i) CsOH (3 equiv), DMF, −20 °C, 24 h; (ii) DMSO, 180 °C, 2 h, MW; (iii) DMSO:MeOH (1:1), HCl, 180 °C, 2 h, MW. Inset: X ray structure of **(*R*_*mp*_*,* 1^5^*S,* 1^7^*R,* 3*R,* 4*S*)-7b**. Intramolecular hydrogen-bond lengths [Å] (angles [deg]): N4–H04···O1 2.12 (136); N5–H05···O2 2.26 (158); N6–H06···O2 2.36 (171).

To gain insight into the observed chirality transmission, we conducted computational investigations focused on the key *trans*-cyclization step, previously described for related fumaramide-based [2]rotaxanes.^18,19d^ These calculations were carried out at the (SMD, DMF)-r2SCAN-3c//wB97XD/def2- SVP theoretical level.^25^ In our computational models, the two butyl groups of the thread were replaced by methyl groups. Thus, we calculated the two transition states **(3*S*, 4*R*)-TS_*Rmp*_** and (**3*R*, 4*S*)-TS_*Rmp*_** which led to the formation of both related interlocked *trans*-lactams **(*R*_*mp*_*,*1^5^*S,*1^7^*R,*3*S,*4*R*)-7b** and **(*R*_*mp*_*,*1^5^*S,*1^7^*R,*3*R,*4*S*)-7b**, respectively (Figure 2). Calculations predicted that the **(3*R*, 4*S*)-TS_*Rmp*_** partner represents the lowest energy transition structure, which leads to the formation of the major isolated lactam **(3*R*, 4*S*)-2b** when starting from rotaxane **(*R*_*mp*_*,*1^5^*S,*1^7^*R*)-1b** (ΔΔ*E*^⧧^ = 11 kJ/mol). To analyze the reason behind this cyclization preference, we decomposed the energy difference, ΔΔ*E*^⧧^, between the two transition structures into the main contributions offered by the differences between the energy of the macrocycles, ΔΔ*E*_mac_, of the threads, ΔΔ*E*_thread_, and also the variances in the interaction energies between both of them, ΔΔ*E*_int_ (see Supporting Information for further details, Table S9).^26^ Whereas the energy differences between the thread and the macrocycle are minima, 2.9 and 0.72 kJ/mol, respectively, the interaction energies between both components at each transition structure are noteworthy, with a difference of 8.9 kJ/mol. The origin of this difference might be due to the formation of CH···π interactions between the N-Me group and one of the phenyl rings of the thread as well as those between the adamantane core and the benzyl group that is forming the new C–C bond. These weak interactions are evident at the **(3*R,*4*S*)-TS_*Rmp*_** transition structure but absent at the **(3*S,*4*R*)-TS_*Rmp*_** one (Figure 2).

**Figure 2:**
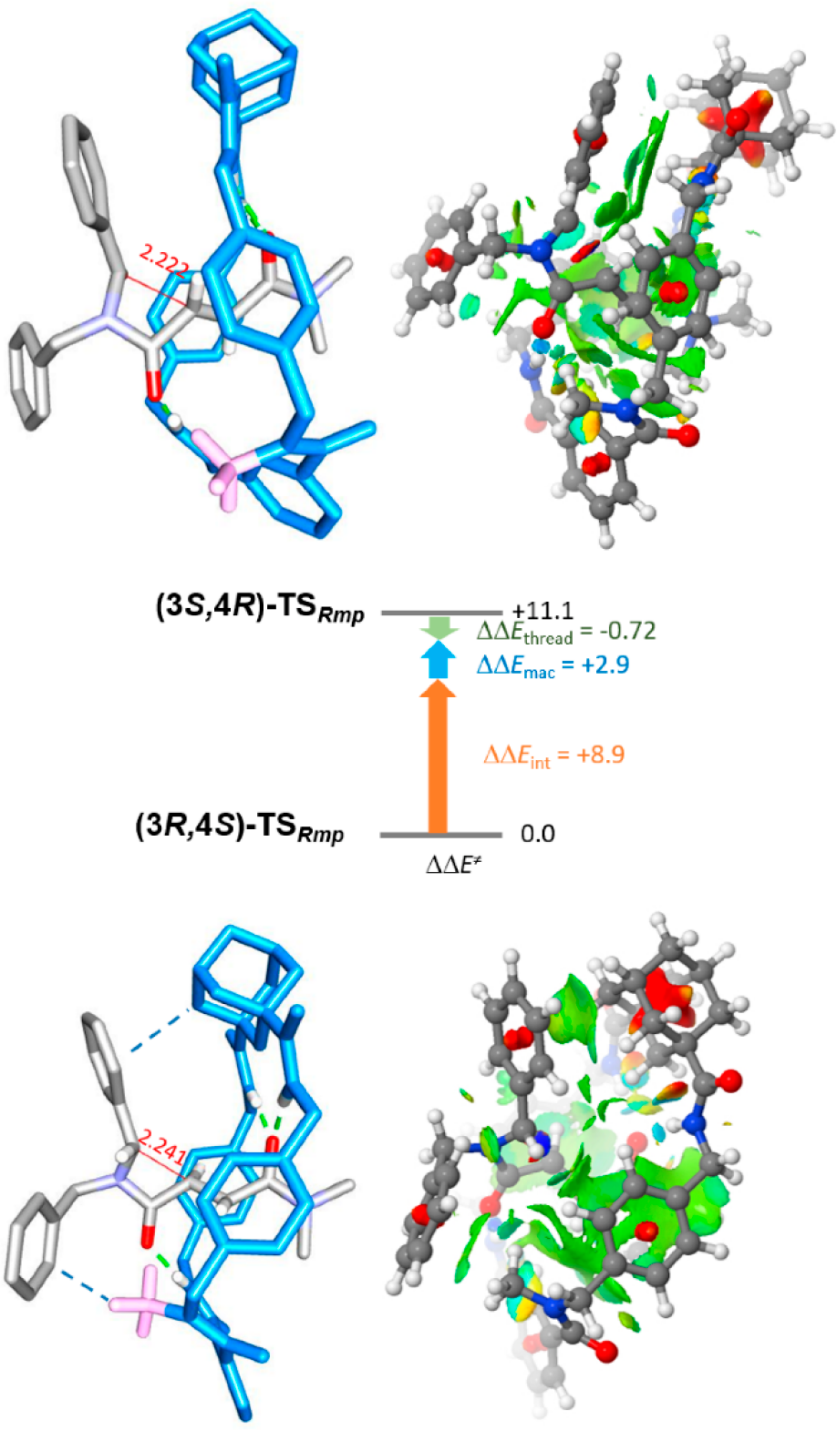
Computed transition structures **(3*S*, 4*R*)-TS_*Rmp*_** and **(3*R*, 4*S*)-TS_*Rmp*_** for the cyclization step (same orientation of the thread) and their noncovalent interaction analysis. The decomposition of the energy difference between **(3*S,*4*R*)-TS_*Rmp*_** and **(3*R*, 4*S*)-TS_*Rmp*_** (ΔΔ*E*^⧧^) into contributions from the energy difference of the macrocycles (ΔΔ*E*_mac_) and threads (ΔΔ*E*_thread_) and between the interaction energies of the thread with the macrocycle (ΔΔ*E*_int_) is shown.

In summary, we have developed a simple preparation of a racemic mechanically planar chiral [2]rotaxane by the desymmetrization of an achiral amide-based [2]rotaxane via a highly selective monomethylation of the entwined macrocycle. After chiral resolution, we explored the transfer of chiral information from the mechanically planar chirality to the newly formed stereogenic centers at the thread during a cyclization process. Pleasantly, the resulting chiral species, isolated as β- and γ-lactams after macrocycle removal, demonstrated high enantiomeric ratios (up to 92:8). This study introduces a novel chirality transfer mode in confined spaces, exemplifying one of the most efficient methods known. It marks a genuine application of MPC-rotaxanes, where the chirality transfer depends on the mechanically planar topology of the initial [2]rotaxane, demonstrating an original approach in this field.

## Supporting Information Available

Supplemental experimental procedures, Figures S1–S9, Schemes S1–S6, Tables S1–S9, Cartesian coordinates of the computed structures, NMR specta, HPLC chromatograms, and supplemental references (PDF)
