# Supplementary material for: Mechanically Planar-to-Point Chirality Transmission in [2]Rotaxanes
Source: J Am Chem Soc. 2024 Jan 24;146(5):2882–7. doi: 10.1021/jacs.3c11611 (PMC10859924; doi:10.1021/jacs.3c11611)
Supplement: Supplementary file 1 — ja3c11611_si_001.pdf [file ja3c11611_si_001.pdf]

Supporting Information for:

Mechanically Planar-to-Point Chirality Transmission in [2]Rotaxanes

Julio Puigcerver,[a] Marta Marin-Luna,[a] Javier Iglesias-Sigüenza,[b] Mateo Alajarin,[a] Alberto Martinez-Cuezva,[a,] Jose Berna,[a,*]*

[a] Departamento de Química Orgánica, Facultad de Química, Regional Campus of International Excellence "Campus Mare Nostrum", Universidad de Murcia, E-30100 Murcia, Spain.
[b] Departamento de Química Orgánica and Centro de Innovacion en Quimica Avanzada (ORFEO-CINQA), Universidad de Sevilla, E-41012 Sevilla, Spain.

Email: amcuezva@um.es; ppberna@um.es

Table of Contents……………………………………………………………………………. S2

1. General experimental section ………………………………………………………… S3

2. Synthesis of threads …………………………………………………………………….. S4

3. Synthesis of [2]rotaxane 4a …………………………………………………………… S4

4. Synthesis of [2]rotaxanes 4b-c ………………………………………………………... S5

5. Selective *N*-Methylation of rotaxanes 4. Screening Conditions ………………………… S7

6. Synthesis of methylated [2]rotaxanes ……………………………………………….. S10

7. Attempts towards the direct asymmetric mono-*N*-methylation of rotaxane 4b ……… S13

8. Optimization of the reaction conditions for the base-catalyzed cyclization of [2]rotaxane 1b and the thermal dethreading of the interlocked lactam 7b. Synthesis of lactams 2 and 3 ……………………………………………………………………………… S15

9. Characterization of the monomethylated macrocycle M1 ………………………………... S21

10. Calculation of the pKa of the deprotonation of model amides ……………………….. S22

11. Crystal data and structure refinements ……………………………………………… S23

12. Determination of the absolute configuration ………………………………………… S30

13. Assignment of the mechanically planar chirality for rotaxane 1b …………………… S32

14. Computational methods …………………………………………………………………. S33

15. ^{1}H and ^{13}C NMR spectra of synthesized compounds …………………………………… S37

16. Copies of HPLC Chromatograms ………………………………………………………. S53

17. References …………………………………………………………………………………. S58

1. General Experimental Section

Unless stated otherwise, all reagents were purchased from Aldrich Chemicals and used without further purification. CsOH (99.9 % purity) was pursached from Alfa Aesar. HPLC grade solvents (Scharlab) were nitrogen saturated and were dried and deoxygenated using an Innovative Technology Inc. Pure-Solv 400 Solvent Purification System. Column chromatography was carried out using silica gel (60 Å, 70-200 μm, SDS) as stationary phase, and TLC was performed on precoated silica gel on aluminun cards (0.25 mm thick, with fluorescent indicator 254 nm, Fluka) and observed under UV light. All melting points were determined on a Kofler hot-plate melting point apparatus and are uncorrected. ^{1}H- and Proton-Decoupled ^{13}C Spectra were recorded on a Bruker Avance 300, 400 and 600 MHz instruments. ^{1}H NMR chemical shifts are reported relative to Me_4Si and were referenced via residual proton resonances of the corresponding deuterated solvent, whereas ^{13}C NMR spectra are reported relative to Me_4Si using the carbon signals of the deuterated solvent. Signals in the ^{1}H and ^{13}C NMR spectra of the synthesized compounds were assigned with the aid of DEPT, APT, or two-dimensional NMR experiments (COSY, HMQC or HMBC). Abbreviations of coupling patterns are as follows: br, broad; s, singlet; d, doublet; t, triplet; q, quadruplet; m, multiplet. Coupling constants (*J*) are expressed in Hz. High-resolution mass spectra (HRMS) were obtained using a time-of-flight (TOF) instrument equipped with electrospray ionization (ESI). The enantiomeric ratios were determined by HPLC analysis employing an Agilent 1100 Series HPLC equipment, and a chiral stationary phase column, specified in the individual experiment, by comparing the samples with the appropriate racemic mixtures. Optical rotation ($[\alpha]_{D}^{25}$) was measured with a JASCO P-2000 polarimeter (concentration g/mL in chloroform as solvent). Circular dichroism was measured with a π*-180 Applied Photophysics spectrometer. The experiments using microwave irradiations were performed in a Discover CEM MW with simultaneous cooling. The power of the equipment was established at 200 W. A microwave vessel (10 mL) equipped with a standard cap (vessel commercially furnished by Discover CEM) and a level of internal pressure maximum of 250 psi was used.

2. Synthesis of threads

Thread T1

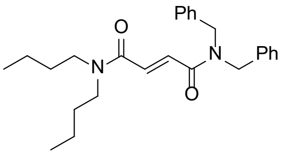

Thread **T1** was prepared by following the described procedure and showed identical spectroscopic data as those reported in bibliography.[1]

Thread T2

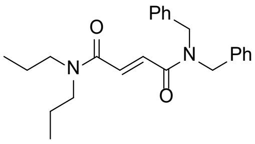

Thread **T2** was prepared following the described procedure and showed identical spectroscopic data as those reported in bibliography.[2]

3. Synthesis of [2]rotaxane 4a

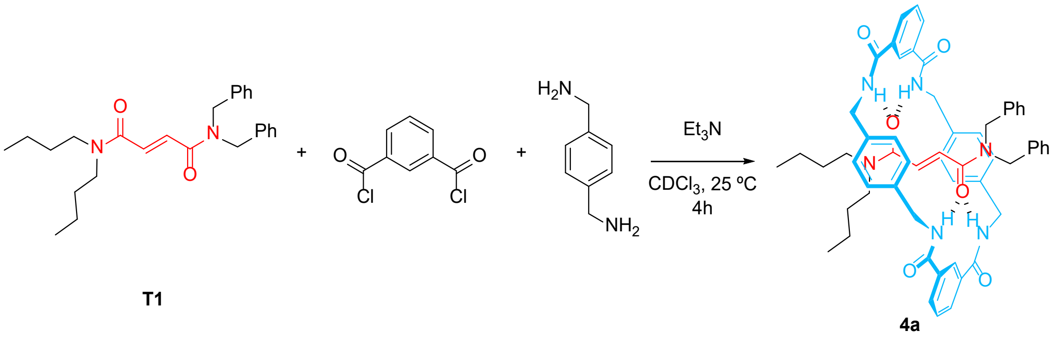

Rotaxane **4a** was prepared following the described procedure and showed identical spectroscopic data as those reported in bibliography.[1]

4. Synthesis of [2]rotaxanes 4b-c

Rotaxane 4b

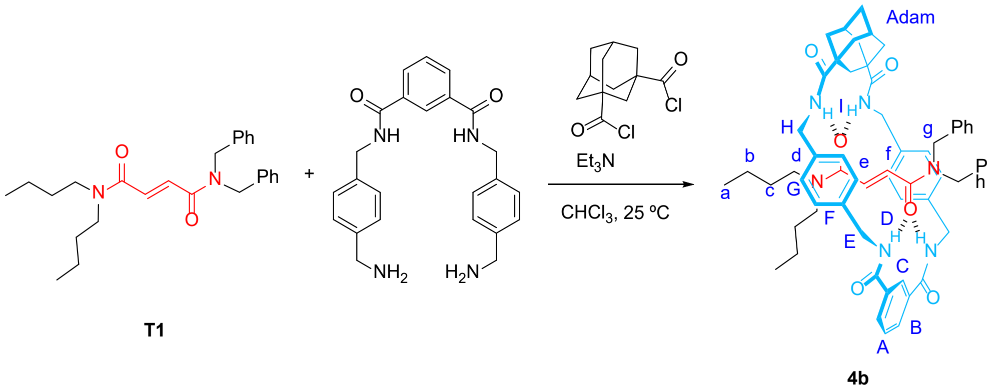

The thread **T1** (430 mg, 1.23 mmol), *N[1],N[3]*-bis[4-(aminomethyl)benzyl]isophthalamide[3] (993 mg, 2.46 mmol) and Et_3N (2.06 mL, 14.8 mmol) in $CHCl_3$ (300 mL) were stirred vigorously whilst solution of 1,3-adamantanedicarbonyl dichloride (640 mg, 2.46 mmol) in $CHCl_3$ (20 mL) was added over a period of 4 h using motor-driven syringe pump. After a further 4 h the resulting suspension was filtered through a Celite® pad, washed with water (2 x 50 mL), a solution of HCl 1M (2 x 50 mL), a saturated solution of $NaHCO_3$ (2 x 50 mL) and brine (2 x 50 mL). The organic phase was dried over $MgSO_4$ and the solvent removed under reduced pressure. The solid crude was subjected to column chromatography on silica gel using a $CHCl_3$/MeOH (40/1) mixture, to give the title product as a white solid (**4b**, 325 mg, 33%). mp 245-246 ºC; *mixture of rotamers (1:0.3) at 298K in $CDCl_3$*; ^1H NMR (400 MHz, $CDCl_3$, 298 K, major rotamer) δ (ppm): 8.88 (s, 1H, H_C), 8.39 (d, *J*= 7.5 Hz, 2H, H_B), 7.69 (t, *J*= 7.5 Hz, 1H, H_A), 7.65-7.30 (m, 9H, H_{Ar+D}), 7.23 (d, *J*= 7.2 Hz, 1H, H_{Ar}), 7.05-6.75 (m, 12H, $H_{F+G+I+Ar}$), 6.02 (s, 2H, H_{e+f}), 5.24 (dd, *J*= 13.6, 9.6 Hz, 2H, H_E), 5.08(dd, *J*= 13.4, 9.3 Hz, 2H, H_H), 4.53 (s, 2H, H_g), 4.43 (s, 2H, $H_{g'}$), 3.50-3.38 (m, 4H, $H_{E'+H'}$), 3.30-3.24 (m, 2H, H_d), 2.94-2.85 (m, 2H, $H_{d'}$), 2.45-0.50 (m, 28H, $H_{c+b+a+Adam}$); ^{13}C NMR (101 MHz, $CDCl_3$, 298 K) δ (ppm): 177.2 (CO), 166.5 (CO), 165.4 (CO), 164.4 (CO), 138.6 (C), 138.0 (C), 135.6 (C), 134.3 (C), 133.6 (C), 132.6 (CH), 130.1 (CH), 129.9 (CH), 129.7 (CH), 129.2 (CH), 129.0 (CH), 127.7 (CH), 126.9 (CH), 122.4 (CH), 51.5 (CH_2), 49.7 (CH_2), 49.2 (CH_2), 47.9 (CH_2), 43.2 (CH_2), 42.8 (CH_2), 41.5 (C), 39.5 (CH_2), 39.0 (CH_2), 38.7 (CH_2), 35.1 (CH_2), 32.4 (CH_2), 30.1 (CH_2), 28.1 (CH), 20.4 (CH_2), 13.9 (CH_3); HRMS (ESI) calcd for $C_{62}H_{73}N_6O_6$ $[M + H]^+$ 997.5586, found 997.5570.

Rotaxane 4c

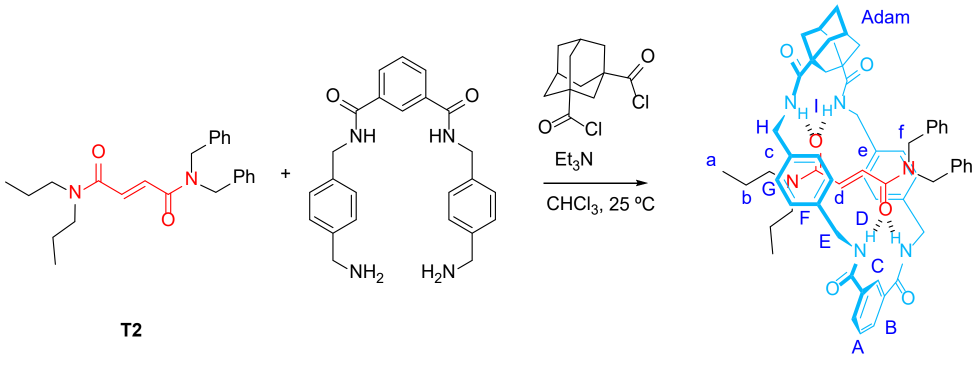

The thread **T2** (700 mg, 1.85 mmol), *N1,N3*-bis[4-(aminomethyl)benzyl]isophthalamide[3] (1.55 g, 3.70 mmol) and Et_3N (3 mL, 22.2 mmol) in $CHCl_3$ (500 mL) were stirred vigorously whilst solution of 1,3-adamantanedicarbonyl dichloride (962 mg, 3.70 mmol) in $CHCl_3$ (20 mL) was added over a period of 4 h using motor-driven syringe pump. After a further 4 h the resulting suspension was filtered through a Celite® pad, washed with water (2 x 50 mL), a solution of HCl 1M (2 x 50 mL), a saturated solution of $NaHCO_3$ (2 x 50 mL) and brine (2 x 50 mL). The organic phase was dried over $MgSO_4$ and the solvent removed under reduced pressure. The solid crude was subjected to column chromatography on silica gel using a $CHCl_3$/MeOH (40/1) mixture, to give the title product as a white solid (**4c**, 215 mg, 12%). mp 220-222 °C; *mixture of rotamers (1:0.3) at 298K in $CDCl_3$*; ^{1}H NMR (600 MHz, $CDCl_3$, 298 K, major rotamer) δ (ppm): 8.88 (s, 1H, H_C), 8.39 (d, *J*= 7.4 Hz, 2H, H_B), 7.69 (t, *J*= 7.4 Hz, 1H, H_A), 7.64-7.35 (m, 8H, H_{Ar+D}), 7.23 (d, *J*= 6.0 Hz, 2H, H_{Ar}), 7.01 (d, *J*= 9.0 Hz, 2H, H_{Ar}), 6.92-6.85 (m, 8H, H_{F+G}), 6.79 (br s, 2H, H_I), 6.00 (s, 2H, H_{d+e}), 5.26 (dd, *J*= 12.9, 9.9 Hz, 2H, H_E), 5.07 (dd, *J*= 13.1, 9.9 Hz, 2H, H_H), 4.53 (s, 2H, H_f), 4.43 (s, 2H, $H_{f'}$), 3.46-3.38 (m, 4H, $H_{E'+H'}$), 3.24-3.21 (m, 2H, H_c), 2.86-2.81 (m, 2H, $H_{c'}$), 2.31-1.49 (m, 16H, $H_{b+c+Adam}$), 1.26-1.21 (m, 2H, H_{adam}), 1.00-0.88 (m, 5H, H_{a+adam}), 0.61 (d, *J*= 11.8 Hz, 2H, H_{adam}), 0.19 (t, *J*= 6.6 Hz, 3H, $H_{a'}$); ^{13}C NMR (150 MHz, $CDCl_3$, 298 K) δ (ppm): 177.2 (CO), 166.5 (CO), 165.4 (CO), 164.5 (CO), 138.6 (C), 138.0 (C), 135.6 (C), 134.3 (C), 133.7 (C), 132.6 (CH), 130.1 (CH), 130.0 (CH), 129.7 (CH), 129.2 (CH), 128.9 (CH), 127.7 (CH), 126.9 (CH), 122.3 (CH), 51.5 (CH_2), 51.0 (CH_2), 50.1 (CH_2), 49.6 (CH_2), 43.2 (CH_2), 42.8 (CH_2), 41.5 (C), 39.5 (CH_2), 38.9 (CH_2), 38.7 (CH_2), 35.1 (CH_2), 28.1 (CH), 23.6 (CH_2), 21.3 (CH_2), 11.9 (CH_3), 10.4 (CH_3); HRMS (ESI) calcd for $C_{60}H_{69}N_6O_6$ $[M + H]^+$ 969.5273, found 997.5282.

5. Selective *N*-Methylation of rotaxanes 4. Screening Conditions.

The selective *N*-monomethylation of rotaxanes **4** was assayed under different conditions, varying the base, solvent and temperature. The conversion into the rotaxanes **1** (monomethylated), **5** (dimethylated) and **6** (tetramethylated) was followed over time by TLC and analytical HPLC provided with a Hypersil Gold (silica, 5 μm) column.

<u>Procedure</u>:

Rotaxane **4** (10 mg, 1 equiv) was placed in a vial dissolved with the suitable solvent (1 mL) under air. The corresponding base was added (5 equiv), the vial capped, and the suspension stirred at the suitable temperature for 1 hour. After this time, MeI (10 equiv) was added and the reaction was stirred for the required time. After this time, the reaction was quenched by the addition of AcOEt (5 mL) and the solution was washed with satured NaCl (3 x 10 mL). The organic phase was dried over $MgSO_4$ and the solvent removed under reduced pressure. The resulting solid was suspended in hexane, filtered and dried. The corresponding mixture of rotaxanes was analyzed by analytical HPLC to calculate the conversion towards the desired monomethylated rotaxanes **1**.

Table S1. Screening conditions for the selective *N*-monomethylation of rotaxanes **1** (only the enantiomer **(*R*$_{mp}$)-4** is shown).

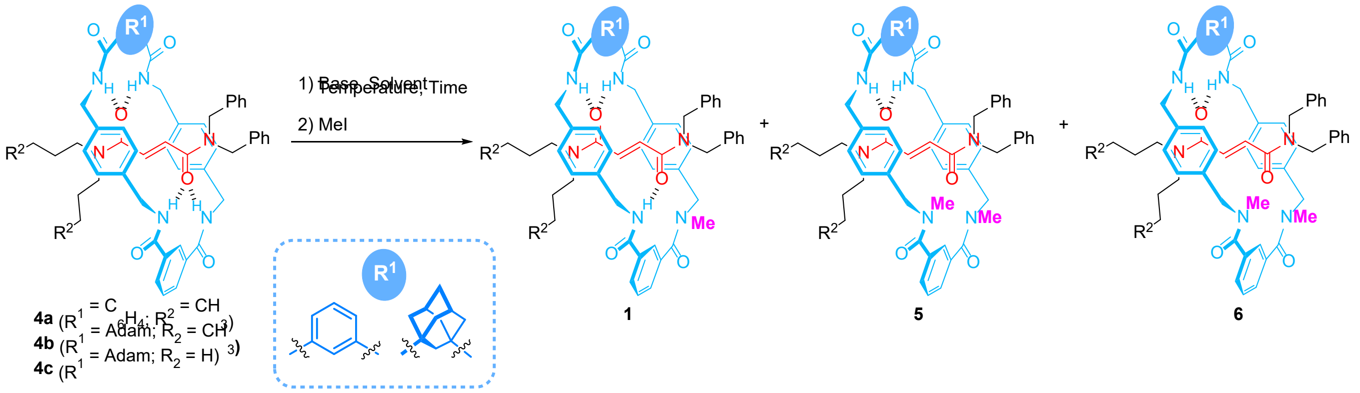

entry	4	solvent	base	T (ºC)	Time (h)	Conversion (%)[a]	ratio 4:1:5:6[a]	selectivity
1	**4a**	THF	NaH	25	1	100	0:0:0:**100**	0
2	**4b**	THF	NaH	25	1	98	2:56:42:0	0.57
3[b]	**4b**	THF	CsOH	25	1	15	85:15:0:0	0.15
4	**4b**	THF	CsOH	25	1	96	4:87:9:0	0.90
5	**4b**	Et_2O	CsOH	25	1	0	100:0:0:0	0
7	**4b**	THF	NaOH	25	1	0	100:0:0:0	0
8	**4b**	THF	KOH	25	1	0	100:0:0:0	0
9	**4b**	THF	CsOH	0	24	95	5:**92**:3:0	0.97
10	**4b**	THF	CsOH	-20	48	27	73:27:0:0	1.00
11[c]	**4c**	THF	CsOH	0	24	-	-	-

[a]Determined by HPLC provided with a Hypersil Gold (silica, 5 µm) column; [b] 1 equiv. of base was used; [c] Monomethylated rotaxane **1c** was unstable and dethreaded.

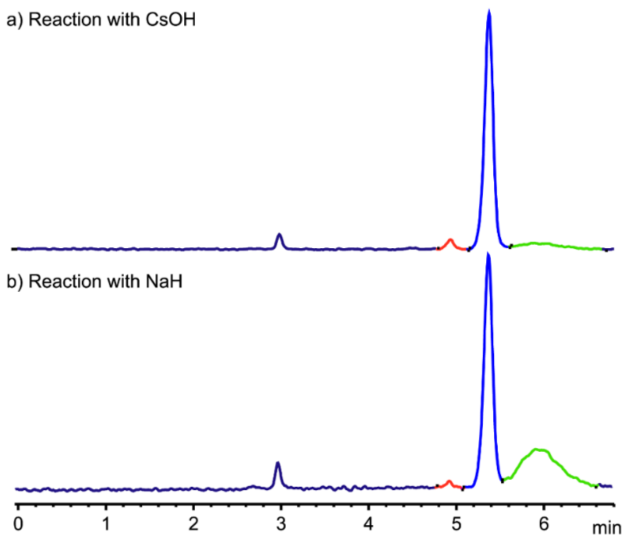

Figure S1. Stack of HPLC chromatograms for the *N*-methylation of rotaxane 4b at 25 ºC in the presence of: a) excess of CsOH; b) excess of NaH. Red colour indicates the remained starting rotaxane **4b**; Blue colour indicates the monomethylated rotaxane **1b**; Green colour indicates the dimethylated rotaxane **5b**. **HPLC conditions**: Hypersil Gold (silica, 5 μm) column, CH_2Cl_2:IPA:NH_4OH (300:2.5:1), 0.75 mL min^{-1}, 280 nm.

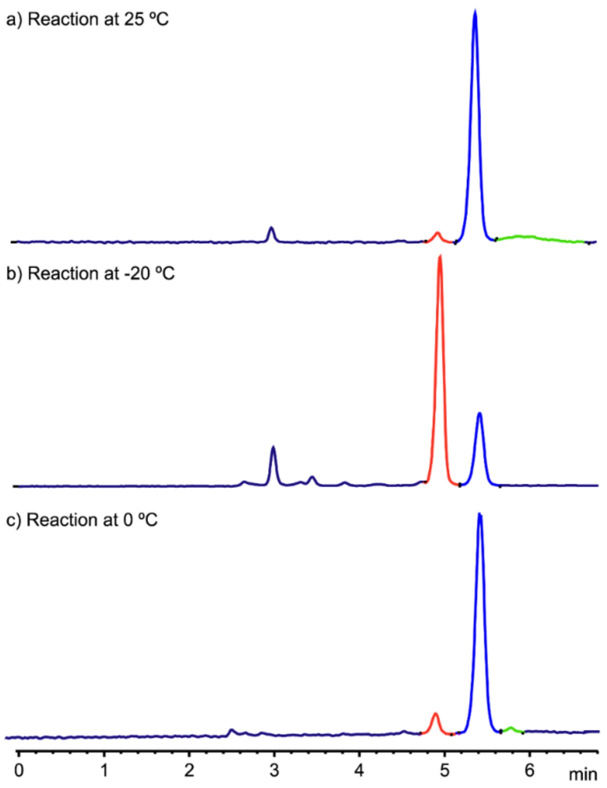

Figure S2. Stack of HPLC chromatograms for the *N*-methylation of rotaxane 4a in the presence of excess of CsOH at different temperatures. Red colour indicates the remained starting rotaxane **4b**; Blue colour indicates the monomethylated rotaxane **1b**; Green colour indicates the dimethylated rotaxane **5b**. **HPLC conditions**: Hypersil Gold (silica, 5 μm) column, CH_2Cl_2:IPA:NH_4OH (300:2.5:1), 0.75 mL min^{-1}, 280 nm.

6. Synthesis of methylated [2]rotaxanes

Rotaxane 6a

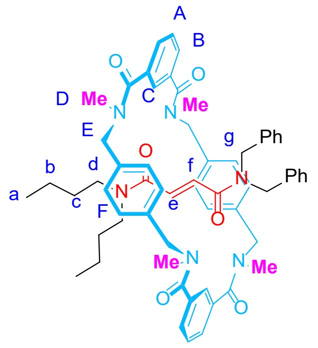

Rotaxane **4a** (50 mg, 0.053 mmol) and NaH 60% dispersion in mineral oil (10 mg, 0.25 mmol) in anhydrous THF (3 mL) were stirred at 0 ºC during 10 min. After this time MeI (150 µL, 2.4 mmol) was added and the solution was washed stirred for 1 h at room temperature. The reaction mixture was quenched with an aqueous solution of HCl 1M (5 mL) and washed with AcOEt (3 x 5mL). The organic phase was dried over $MgSO_4$ and the solvent removed under reduced pressure. The resulting solid was suspended in Et_2O, filtered and dried, to yield the corresponding compound (**6a**, 44 mg, 83%). mp > 300 ºC; ^{1}H NMR (400 MHz, $C_2D_2Cl_4$, 373 K) δ (ppm): 8.01 (s, 1H, H_C), 7.60-7.15 (m, 24H, $H_{Ar+A+B+C+F}$), 7.04-6.83 (m, 3H, H_{Ar+e+f}), 4.70-4.25 (m, 12H, H_{E+g}), 3.40-3.33 (m, 2H, H_d), 3.26-3.06 (m, 2H, $H_{d'}$), 2.93 (s, 6H, H_D), 2.89 (s, 6H, $H_{D'}$), 1.63-1.52 (m, 2H, H_c), 1.40-0.80 (m, 6H, $H_{c'+b}$), 1.00-0.93 (m, 3H, H_a), 0.87-0.75 (m, 3H, H_a); ^{13}C NMR (101 MHz, $C_2D_2Cl_4$, 373 K) δ (ppm): 173.1 (CO), 172.8 (CO), 167.0 (CO), 166.8 (CO), 165.5 (CO), 139.4 (C), 139.1 (C), 139.0 (C), 138.6 (C), 138.5 (C), 135.0 (C), 134.9 (C), 133.2 (C), 131.2 (CH), 130.9 (CH), 130.5 (CH), 130.3 (CH), 130.2 (CH), 130.0 (CH), 129.8 (CH), 129.7 (CH), 129.3 (CH), 127.3 (C), 54.4 (CH_2), 53.8 (CH_2), 39.6 (CH_3), 38.1 (CH_3), 33.9 (CH_2), 32.0 (CH_2), 22.3 (CH_2), 15.8 (CH_3); HRMS (ESI) calcd for $C_{62}H_{71}N_6O_6$ $[M + H]^+$ 995.5430, found 995.5396.

Rotaxane *rac*-1b (only the enantiomer **(*R_{mp}*, 1⁵*S*, 1⁷*R*)-1b** is shown)

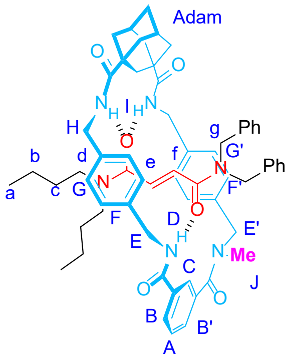

Under the optimized conditions, rotaxane **4a** (150 mg, 0.15 mmol) was placed in a vial and THF was added (5 mL) under air. CsOH was added (113 mg, 0.75 mmol), the vial capped, and the suspension stirred at 0 ºC for 1 hour. After this time, MeI (93 µL, 1.50 mmol) was added and the reaction was stirred at 0 ºC for 24 hours. After this time, the reaction was quenched by the addition of AcOEt (20 mL) and the solution was washed with satured NaCl (5 x 10 mL). The organic phase was dried over $MgSO_4$ and the solvent removed under reduced pressure. The solid crude was subjected to preparative Thin Layer Chromatography on silica gel using a $CHCl_3$/MeOH (40/1) mixture, to give the title product as a white solid (***rac*-1b**, 136 mg, 90%). mp 198-200 ºC; ^{1}H NMR (400 MHz, $CDCl_3$, 318 K, δ (ppm): 8.48 (s, 1H, H_C), 8.27-8.18 (m, 2H, $H_{B+B'}$), 7.65 (d, *J*= 6.3 Hz, 1H, H_D), 7.58-7.53 (m, 1H, H_A), 7.34-7.21 (m, 8H, H_{Ph}), 7.11-6.73 (m, 12H, $H_{Ph+F+F'+G+G'+I}$), 6.30-6.17 (m, 2H, H_{e+f}), 5.34-3.53 (m, 12H, $H_{E+E'+H+H'+g+g'}$), 3.33-3.23 (m, 2H, H_d), 3.07-2.92 (m, 5H, $H_{J+d'}$), 2.14-0.71 (m, 28H, $H_{Adam+a+b+c}$); ^{13}C NMR (101 MHz, $CDCl_3$, 318 K) δ (ppm): 177.8 (CO), 177.3 (CO), 170.5 (CO), 165.7 (CO), 165.6 (CO), 164.7 (CO), 138.1 (C), 137.7 (C), 137.0 (C), 136.4 (C), 136.2 (C), 134.9 (C), 133.7 (C), 130.8 (CH), 130.7 (CH), 130.4 (CH), 129.6 (CH), 129.4 (CH), 129.3 (CH), 129.1 (CH), 128.9 (CH), 128.7 (CH), 128.5 (CH), 128.4 (CH), 126.8 (CH), 124.2 (CH), 51.9 (C), 51.5 (CH_2), 49.8 (CH_2), 49.0 (CH_2), 47.7 (CH_2), 43.6 (C), 43.5 (C), 41.7 (C), 39.3 (CH_2), 39.1 (CH_2), 38.9 (CH_3), 38.6 (CH_2), 35.4 (CH_2), 32.3 (CH_2), 30.1 (CH_2), 28.4 (CH_3), 20.5 (CH_2), 13.9 (CH_3); HRMS (ESI) calcd for $C_{63}H_{75}N_6O_6$ $[M + H]^+$ 1011.5743, found 1011.5729.

Rotaxane 5b

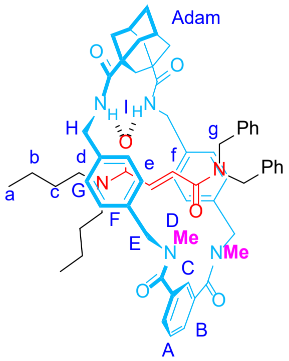

NOTE: under the optimized conditions, the dimethylated rotaxane **5b** is obtained in a minor amount. Thus, for the isolation and characterization of rotaxane **5b**, we employed NaH as the base.

Rotaxane **4b** (100 mg, 0.10 mmol) was placed in a vial and THF was added (5 mL) under air. NaH 60% dispersion in mineral oil (20 mg, 0.50 mmol) was added, the vial capped, and the suspension stirred at 0 ºC for 1 hour. After this time, MeI (62 µg, 1.00 mmol) was added and the reaction was stirred at 25 ºC for an additional hour. After this time, the reaction was quenched by the addition of AcOEt (20 mL) and the solution was washed with satured NaCl (5 x 10 mL). The organic phase was dried over $MgSO_4$ and the solvent removed under reduced pressure. The solid crude was subjected to preparative Thin Layer Chromatography on silica gel using a $CHCl_3$/MeOH (40/1) mixture, to give the title product as a white solid (**5b**, 30.7 mg, 30%). mp 216-218 ºC; ^{1}H NMR (400 MHz, $CDCl_3$, 318 K) δ (ppm): 7.84-6.49 (m, 24H, $H_{Ph+A+B+C+F+G+I}$), 6.10 (br s, 2H, H_{e+f}), 4.58-3.95 (m, 12H, H_{g+E+H}), 3.31-2.83 (m, 10H, H_{D+d}), 2.29-0.88 (m, 28H, $H_{a+b+c+Adam}$); ^{13}C NMR (101 MHz, $CDCl_3$, 318 K) δ (ppm): 177.7 (CO), 171.2 (CO), 164.9 (CO), 164.4 (CO), 137.5 (C), 137.0 (C), 136.7 (C), 136.1 (C), 130.2 (CH), 129.8 (CH), 129.3 (CH), 129.1 (CH), 128.7 (CH), 128.4 (CH), 128.0 (CH), 127.1 (CH), 54.8 (C), 51.4 (CH_2), 48.5 (CH_2), 47.3 (CH_2), 44.3 (CH_2), 43.5 (CH_2), 41.7 (C), 39.2 (CH_2), 38.9 (CH_2), 38.5 (CH_2), 35.5 (CH_2), 32.5 (CH_2), 30.2 (CH_2), 28.5 (CH), 28.3 (CH), 20.6 (CH_2), 20.1 (CH_2), 13.9 (CH_3); HRMS (ESI) calcd for $C_{64}H_{77}N_6O_6$ $[M + H]^+$ 1025.5899, found 1025.5901.

7. Attempts towards the direct asymmetric mono-*N*-methylation of rotaxane 4b.

Several experiments for the direct asymmetric mono-*N*-methylation reaction of rotaxane **4b** were assayed. Two different vias were tested: 1) use of chiral lithium amides for the selective deprotonation, followed by the addition of MeI; 2) use of a chiral methylating reagent. Under the different conditions tested, we always found the desired monomethylated rotaxane **1b** as a racemic mixture. These experiments are summarized below.

7.1. Employment of chiral lithium amide

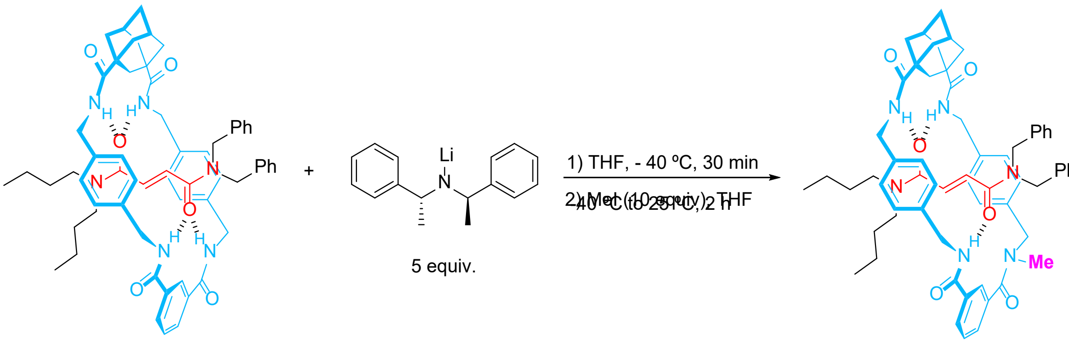

4b **1b, 50:50 er**

Scheme S1. Methylation of rotaxane **4b** employing a chiral lithium amide as the base (only the enantiomer **(*R*$_{mp}$, 1^{5}*S*, 1^{7}*R*)-1b** is shown).

<u>Procedure:</u> to a solution of rotaxane **4b** in anhydrous THF under N_2 atmosphere at - 40 ºC, lithium bis((*R*)-1-phenylethyl)amide[4] (5 equiv) was added. After stirring for 30 min, MeI (10 equiv) was added and the reaction mixture was stirred for 2 hours at 25 ºC. After this time, the reaction was quenched by the addition of water and extract with AcOEt. After purification of the desired **1b** by preparative TLC, the enantiomeric exccess was analyzed by chiral HPLC (racemate, 50:50 e.r.).

7.2. Employment of chiral methylating reagents

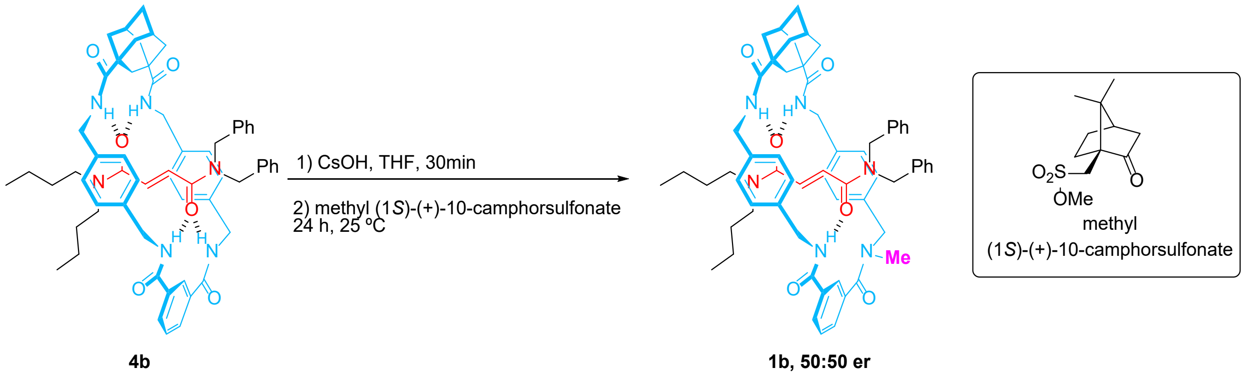

Scheme S2. Methylation of rotaxane **4b** employing a chiral methylating reagent (only the **(*R*$_{mp}$, 1^5*S*, 1^7*R*)-1b** is shown).

Procedure: to a solution of rotaxane **4b** in anhydrous THF at - 40 ºC, CsOH (5 equiv) was added. After stirring for 30 min, methyl (1*S*)-(+)-10-camphorsulfonate (10 equiv) was added and the reaction mixture was stirred for 24 hours at 25 ºC. After this time, the reaction was quenched by the addition of water and extract with AcOEt. After purification of the desired **1b** by preparative TLC, the enantiomeric exccess was analyzed by chiral HPLC (racemate, 50:50 e.r.).

8. Optimization of the reaction conditions for the base-catalyzed cyclization of [2]rotaxane 1b and the thermal dethreading of the interlocked lactam 7b. Synthesis of lactams 2 and 3.

The conditions for the base-catalyzed cyclization of rotaxane **1b** were optimized. In order to accurately measure the conversion and the enantioselectivity of the process, we performed a 2 step-protocol: 1) cyclization in the presence of base to obtain the interlocked lactam **7b**; 2) thermal dethreading (MW irradiation) for the obtention of the non-interlocked lactam **2b**, which its enantiopurity can be easily analyzed by chiral HPLC. Previous studies on the synthesis of the enantioenriched lactam **2b** suggested that CsOH and DMF were the best base and solvent, respectively.[5] Thus we measured the enantiomeric ratio of lactam **2b** (after thermal dethreading) when the cyclization was carried out at different temperatures (Table S2). The addition of exces of base is required in order to accelerate the process.

<u>NOTE:</u> The reaction carried out at lower temperatures (-30 ºC) were extremely slow, thus we settled the best temperature at -20 ºC. We performed the screening reactions by using the **enantiomer 1** (first eluting rotaxane) of rotaxane **1b.** The reaction with the **enantiomer 2** (second eluting rotaxane) was also carried out under the best conditions.

Table S2. Optimization of the temperature.

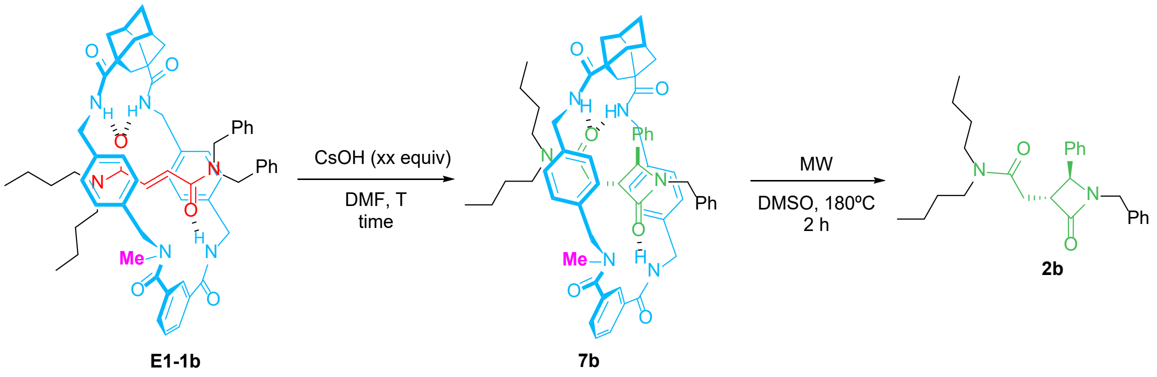

entry	T (ºC)	CsOH (equiv)	Time (h)	Conversion (%)[a]	e.r.[b]
1	25	1	1	100	22:78
2	0	2	24	100	15:85
3	-20	5	24	100	10:90
4[c]	-20	5	24	100	91:9

[a] Determined by TLC; [b] Determined by HPLC provided with a ChiralPak IC3 column; [c] From **enantiomer 2** of rotaxane **1b**.

Interlocked *trans*-β-lactam *rac*-7b

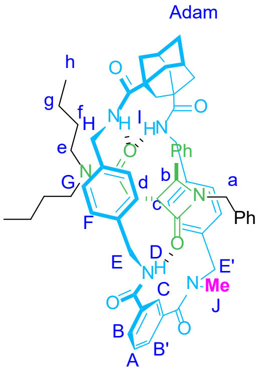

Rotaxane ***rac*-1b** (20 mg, 0.02 mmol) and CsOH (14 mg, 0.1 mmol) in anhydrous DMF (2 mL) were stirred vigorously at -20 ºC for 24 hours. After this time, the reaction was quenched by the addition of AcOEt (20 mL) and the solution was washed with satured NaCl (5 x 10 mL). The resulting residue was subjected to column chromatography (silica gel) by using a $CHCl_3$/MeOH (40/1) mixture as eluent to yield the corresponding *trans*-β-lactam **7b** (as a racemate) as a white solid (19 mg, 95%). mp 97-99 ºC; ^{1}H NMR (400 MHz, $CDCl_3$, 318 K) δ (ppm): 9.02 (br s, 1H, H_D), 8.19 (d, *J*= 7.7 Hz, 1H, H_B), 7.66-7.55 (m, 3H, $H_{A+B'+C}$), 7.45-7.24 (m, 7H, H_{Ph+I}), 7.17-6.77 (m, 12H, H_{Ph+F+G}), 5.94 (br s, 1H, $H_{I'}$), 5.50 (br s, 1H, $H_{E'}$), 5.26 (br s, 1H, H_E), 4.43 (d, *J*= 14.5 Hz, 2H, H_a), 4.27 (m, 2H, $H_{H+H'}$), 4.07-3-92 (m, 2H, H_{E+H}), 3.48-3.36 (m, 3H, $H_{E'+H'+b}$), 3.25-3.17 (m, 1H, H_e), 3.10-3.01 (m, 1H, H_e), 2.96-2.70 (m, 6H, $H_{e'+e'+Adam}$), 2.17-1.97 (m, 9H, $H_{d+J+Adam}$), 1.74-1.14 (m, 14H, $H_{c+f+f'+g+g'+Adam}$), 0.95-0.79 (m, 4H, $H_{d'+h}$), 0.69 (br s, 3H, $H_{h'}$); ^{13}C NMR (101 MHz, $CDCl_3$, 318 K) δ (ppm): 178.4 (CO), 177.8 (CO), 170.8 (CO), 170.2 (CO), 169.9 (CO), 166.8 (CO), 139.0 (C), 137.0 (C), 136.9 (C), 136.3 (C), 135.9 (C), 135.4 (C), 134.6 (C), 131.0 (CH), 130.8 (CH), 130.6 (CH), 130.3 (CH), 130.0 (CH), 129.8 (CH), 129.5 (CH), 129.2 (CH), 129.1 (CH), 128.9 (CH), 128.7 (CH), 128.5 (CH), 128.0 (CH), 123.9 (CH), 62.1 (CH), 52.6 (CH), 51.6 (CH_2), 49.4 (CH_2), 47.6 (CH_2), 45.7 (CH_2), 45.2 (CH_2), 44.4 (CH_2), 44.0 (CH_2), 41.8 (C), 41.5 (C), 39.9 (CH_2), 39.7 (CH_2), 38.6 (CH_2), 38.4 (CH_2), 38.1 (CH), 35.4 (CH_2), 32.4 (CH_2), 31.4 (CH_2), 30.6 (CH_2), 29.8 (CH_2), 28.4 (CH_3), 28.3 (CH_3), 20.8 (CH_2), 19.8 (CH_2), 13.8 (CH_3), 13.7 (CH_3); HRMS (ESI) calcd for $C_{63}H_{75}N_6O_6$ $[M + H]^+$ 1011.5743, found 1011.5742.

NOTE: the same reaction was performed with both enantiomers of rotaxane **1b** (10 mg scale). After purification, the optical rotation was measured.

From **E1-1b**: 9.0 mg, 90 % yield, $[\alpha]_D^{25}$ = - 28 (*c* 0.0035, $CHCl_3$).

From **E2-1b**: 8.5 mg, 85 % yield, $[\alpha]_D^{25}$ = + 26 (*c* 0.003, $CHCl_3$).

In the base-triggered cyclization reaction of **1b**, four *trans*-stereoisomers of the interlocked lactam **7b** can be obtained, two from each enantiomer of rotaxane **1b** (Scheme S3).

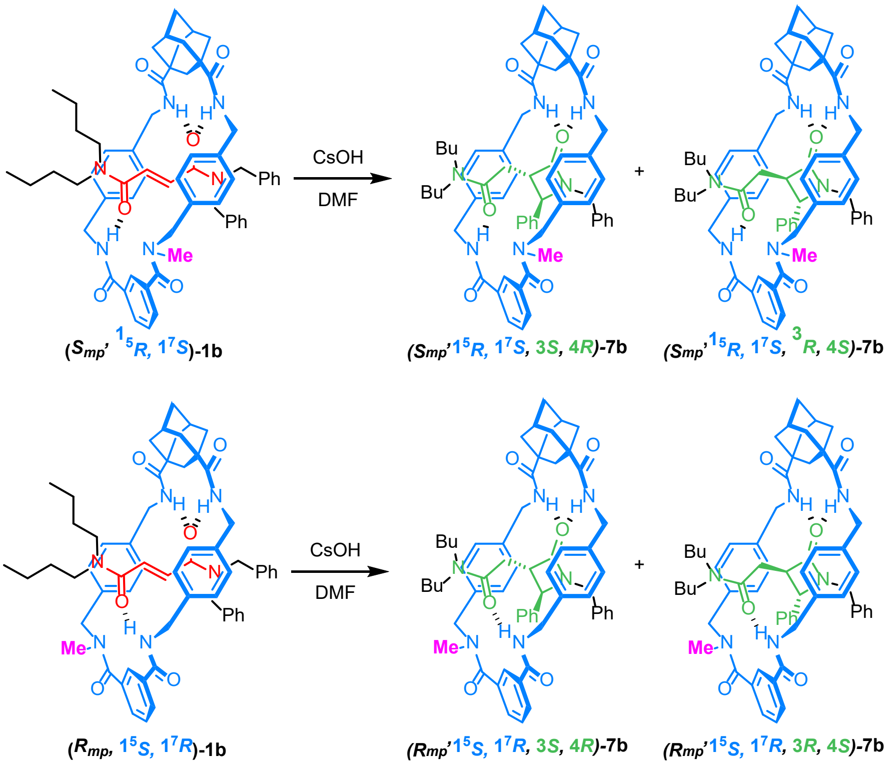

Scheme S3. Possible stereoisomers of **7b** obtained obatained in the intramolecular cyclization of both enantiomers of rotaxane **1b**.

In order to separate the four stereiomers obtained when ***rac*-1b** is employed, we tested different HPLC conditions, including the use of different columns and solvent mixtures. The best conditions found were: Chiralpak IC-3 column, CH_2Cl_2:CH_3CN:IPA (85:13:2), 0.5 mL/min, 20 ºC, 254 nm. Three peaks were observed: 10 min (major peak), 13 min and 15 min (both with similar areas) (Figure S3a). The HPLC chromatogram obtained from the cyclization reaction of **E1-1b** showed only two peaks: 10 min (major), 13 min (minor) (Figure S3b). In the case of the reaction from **E2-1b**, two peaks were observed: 10 min (major), 15 min (minor) (Figure S3c). The main stereoisomers of the interlocked lactam **7b**, starting from both enantiopure enantiomers of **1b**, eluted at similar retention time (t_R = 10 min). From this data we can concluded that, as we initially envisioned, the intramolecular cyclization of the rotaxane **1b** occurrs in a diastereoselective manner.

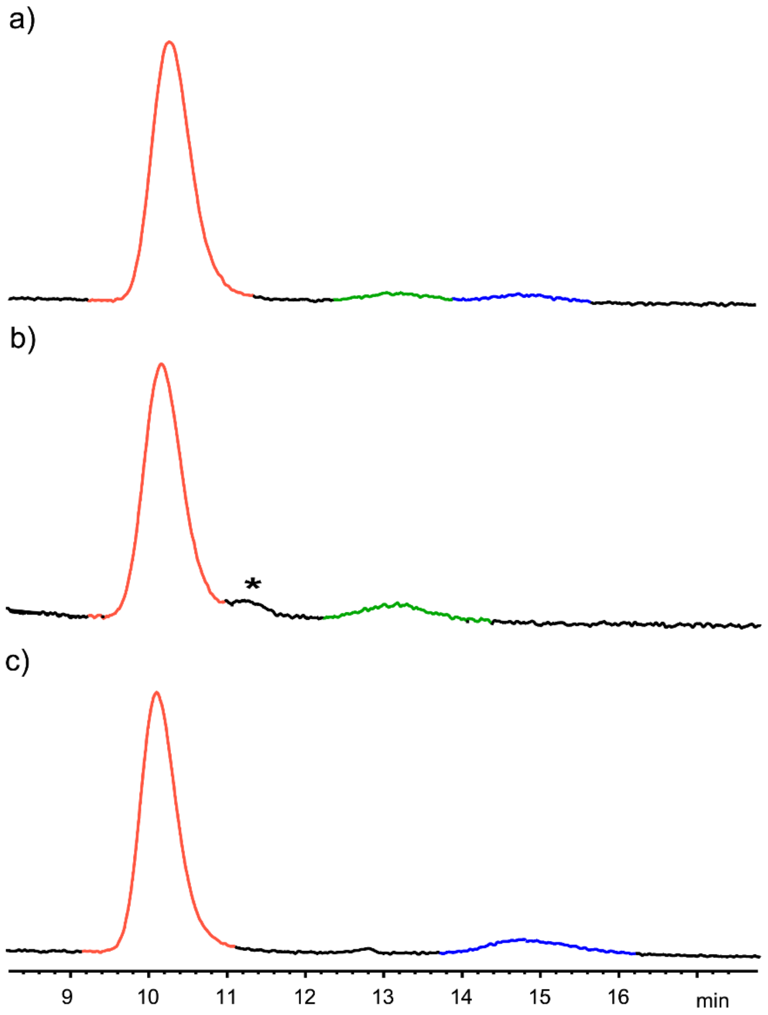

Figure S3. HPLC chromatograms of the mixture of stereoisomers of the interlocked lactam **7b** obtained from: a) ***rac*-1b**; b) **E1-1b**; c) **E2-1b**. Traces of the dimethylated rotaxane **5b** is marked with an asterisk. NOTE: Cyclization reactions were carried out at – 20ºC. **HPLC conditions**: Chiralpak IC-3 column, CH_2Cl_2:MeCN:IPA (85:13:2), 0.5 mL min^{-1}, 254 nm.

***trans*-β-lactam 2b**

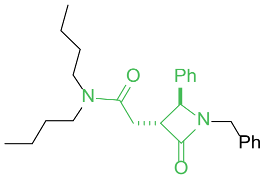

Interlocked *trans*-lactam **7b** (1 equiv.) in DMSO (2 mL) were stirred at 180 ºC for 3 hours under MW irradiation, or alternatively at 120 ºC for 3 days under conventional heating. After this time, the reaction was quenched by the addition of AcOEt (5 mL) and the solution was washed with satured NaCl (5 x 10 mL). The resulting residue was subjected to preparative TLC (silica gel) by using a hexane/Et_2O (1/2) as eluent to yield the corresponding *trans*-β-lactam **2b** as a yellow oil.

This procedure was followed with both interlocked lactams **7b** obtained from each enantiomer of rotaxane **1b** (20 mg scale). After purification, the optical rotation and the enantiomeric ratio were measured.

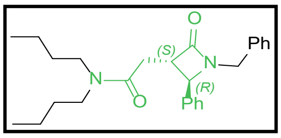

From **E1-1a**: **(3*S*, 4*R*)-2b**

7.0 mg, 87 % yield

$[\alpha]_D^{25}$ = + 18 (*c* 0.0012, $CHCl_3$); 10:90 er

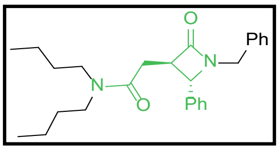

From **E2-1a**: **(3*R*,4*S*)-2b**

7.1 mg, 88 % yield

$[\alpha]_D^{25}$ = - 21 (*c* 0.001, $CHCl_3$); 91:9 er

***cis*-γ-lactam 3b**

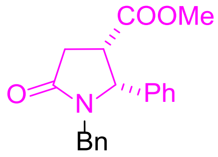

Rotaxane **7b** (10 mg, 1 equiv.) was dissolved in a mixture of DMSO (1 mL), MeOH (1 mL) and HCl 1M (0.1 mL) and was stirred at 120 ºC for 3 days under conventional heating. After this time the reaction was quenched by the addition of AcOEt (5 mL) and the solution was washed with satured NaCl (5 x 10 mL). The resulting residue was subjected to preparative TLC (silica gel) by using a hexane/Et_2O (1/2) mixture as eluent to yield the corresponding *cis*-γ-lactam **3b** as a white solid.

This procedure was followed by using the interlocked lactams obtained from both enantiomers of rotaxane **1b**. After purification, the optical rotation and the enantiomeric ratio were measured.

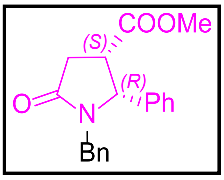

From **E1-1b**: (4*S*, 5*R*)-**3b**

2.6 mg, 87 % yield

$[\alpha]_D^{25}$ = - 5.2 (*c* 0.001, $CHCl_3$); 8:92 er

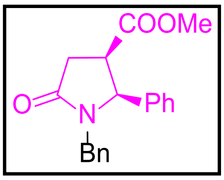

From **E1-1b**: (4*R*,5*S*)-**3b**

2.7 mg, 90 % yield

$[\alpha]_D^{25}$ = + 6.0 (*c* 0.001, $CHCl_3$); 92:8 er

9. Characterization of the monomethylated macrocycle M1

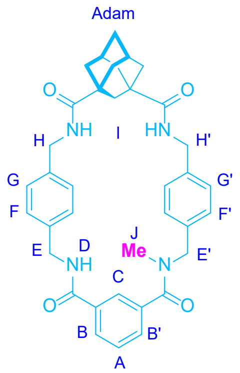

Macrocycle **M1** can be recovered during the purification of the lactams **2b** and **3b**. Starting from rotaxane ***rac*-1b** (20 mg) the title compound was isolated by preparatve TLC as a white solid (9 mg, 75%). mp 97-99 ºC; ^{1}H NMR (300 MHz, DMSO-d_6, 353 K) δ (ppm): 8.76 (br s, 1H, H_D), 7.92 (dt, *J*= 6.7, 2.0 Hz, 1H, H_C), 7.82 (br s, 1H, H_B), 7.61-7.50 (m, 4H, $H_{A+B'+I}$), 7.29-7.16 (m, 8H, $H_{F+F'+G+G'}$), 4.56 (br s, 2H, $H_{E'}$), 4.46 (d, *J*= 5.8 Hz, 2H, H_E), 4.26 (dd, *J*= 5.7, 4.3 Hz, 4H, $H_{H+H'}$), 2.86 (s, 3H, H_J), 2.12 (br s, 2H, H_{Adam}), 1.91-1.63 (m, 12H, H_{Adam}); ^{13}C NMR (75 MHz, DMSO-d_6, 353 K) δ (ppm): 175.9 (CO), 175.6 (CO), 169.5 (CO), 165.3 (CO), 138.6 (C), 138.3 (C), 137.2 (C), 136.2 (C), 135.0 (C), 134.7 (C), 128.4 (CH), 127.9 (CH), 127.8 (CH), 127.0 (CH), 126.9 (CH), 126.9 (CH), 126.7 (CH), 124.5 (CH), 42.2 (CH_2), 41.5 (CH_2), 40.2 (C), 40.1 (CH_2), 39.8 (CH_2), 39.5 (CH_2), 37.6 (CH_2), 37.5 (CH_2), 35.0 (C), 27.6 (CH_3); HRMS (ESI) calcd for $C_{37}H_{41}N_4O_4$ $[M + H]^+$ 605.3122, found 605.3126.

10. Calculation of the pKa of the deprotonation of model amides.

The pKa of the NH amide groups has been calculated for two model molecules: a) *N*-benzylbenzamide and b) *N*-benzyladamantane-1-carboxamide, which mimick the amides present at the macrocycle in rotaxane **1b** (Scheme S4). The calculations were carried out using the software package Marvin Suite version 5.11.5 and chemicalize.org, both developed by ChemAxom.[6] The obtained data indicates the main acidic character of the NH group in the *N*-benzylbenzamide compared to that of the *N*-benzyladamantane-1-carboxamide.

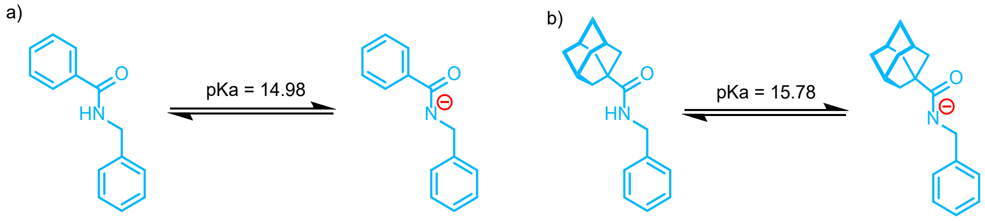

Scheme S4. Calculation of the pK_a for the deprotonation of: a) *N*-benzylbenzamide and b) *N*-benzyladamantane-1-carboxamide.

11. Crystal data and structure refinements.

Rotaxane (*R_{mp}*, 1[5]*S*, 1[7]*R*)-1b

Single crystals of $C_{63}H_{74}N_6O_6$ [AMC_F2_0msp15s_a] were obtained by slow diffusion of pentane placed over a solution of the titled compound in CH_2Br_2. Intensities were registered at low temperature (100.0 K) on a Bruker D8 QUEST system equipped with a multilayer monochromator and a Mo K/a Incoatec microfocus sealed tube (λ = 0.71073 Å). Absorption corrections were based on multi-scans (program SADABS). Using Olex2,[7] the structure was solved with the SHELXT[8] structure solution program using Intrinsic Phasing and refined with the SHELXL[9] refinement package using Least Squares minimisation. Hydrogen atoms were included using a riding model. The structure was deposited with deposition number CCDC 2299443.

Table S3. Crystal data and structure refinement for **(*R_{mp}*, 1[5]*S*, 1[7]*R*)-1b**

Empirical formula	$C_{63}H_{74}N_6O_6$
Formula weight	1011.28
T [K]	100
Wavelength [Å]	0.71073
Crystal system	Monoclinic
Space group	$P2_1$
a (Å)	10.8153(6)
b (Å)	20.2223(11)
c (Å)	12.3818(6)
α (°)	90
β (°)	91.470(2)
γ (°)	90
V [Å^3]	2707.1(2)
Z	2
ρ [g·cm^{-3}]	1.241
μ [mm^{-1}]	0.080
F_{000}	1084
Crystal size [mm^3]	0.32 × 0.21 × 0.12
2Θ range (º)	3.768 to 57.628
h	-14 to 14

k	-27 to 27
l	-16 to 16
Reflections collected	115239
Independent reflections	14051
R(int)	0.0330
Refinement method	Full-matrix least-squares on F2
Parameters	692
Restraints	1
Goodness-of-fit on F^2	1.081
$R1$ [I > 2σ (I)]	0.0366
$wR2$ [I > 2σ (I)]	0.0809
$R1$ (all data)	0.0439
$wR2$ (all data)	0.0887
Δ*ρ* [e·Å^{-3}]	0.23/-0.21
Flack Parameter	0.0(8)

Table S4. Hydrogen bonds for rotaxane ***$(R_{mp}$, 1^5S, $1^7R)$*-1b** [Å and (°)].

D-H...A	d(D-H)	d(H...A)	d(D...A)	<(DHA)
N4-H(04A)...O1	0.89(3)	2.06(3)	2.932(2)	166 (2)
N5-H(05)...O2	0.86(3)	2.07(3)	2.890(2)	161 (2)

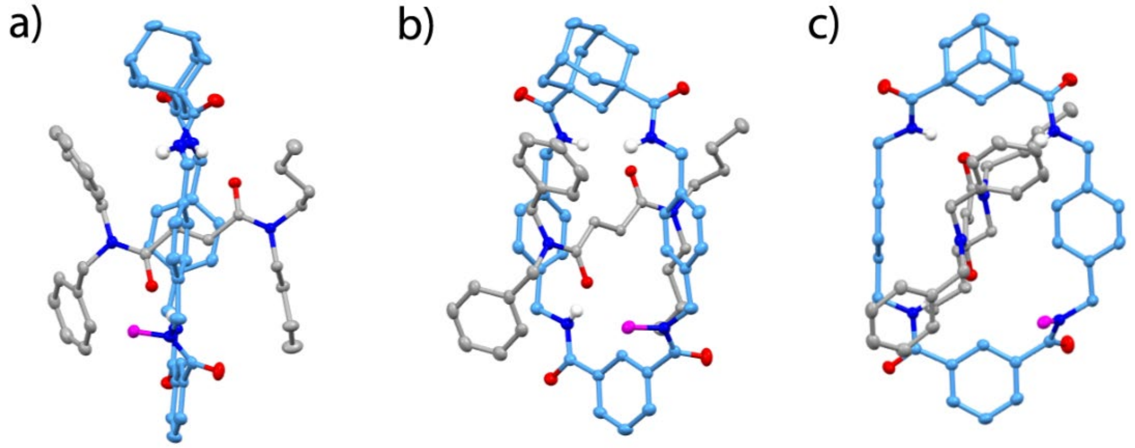

Figure S4. Molecular structure of ***$(R_{mp}$, 1^5S, $1^7R)$*-1b** with thermal ellipsoids drawn at 50% probability: a) lateral view; b) tilted view; c) front view. For clarity, solvent molecules and selected hydrogens atoms have been deleted.

Rotaxane 5b

Single crystals of $C_{64}H_{76}N_6O_6$ [AMC_VII_053_dimet_0msp_a] were obtained by slow diffusion of pentane over a solution of the titled compound in CH_2Cl_2. Intensities were registered at low temperature (100.0 K) on a Bruker D8 QUEST system equipped with a multilayer monochromator and a Mo K/a Incoatec microfocus sealed tube (λ = 0.71073 Å). Absorption corrections were based on multi-scans (program SADABS). Using Olex2,[7] the structure was solved with the SHELXT[8] structure solution program using Intrinsic Phasing and refined with the SHELXL[9] refinement package using Least Squares minimisation. Hydrogen atoms were included using a riding model. Solvent molecules could not be modelled satisfactorily and were therefore removed from the electon density map using the OLEX2 solvent mask command. 40 electrons were found in a volume of 338 $Å^3$ in 1 void per unit cell. This is consistent with the presence of 1 molecule of CH_2Cl_2 per Unit Cell which account for 42 electrons. The structure was deposited with deposition number CCDC 2299445.

Table S5. Crystal data and structure refinement for **5b**

Empirical formula	$C_{64}H_{76}N_6O_6$
Formula weight	1025.30
T [K]	100
Wavelength [Å]	0.71073
Crystal system	Triclinic
Space group	P-1
a (Å)	11.2795(11)
b (Å)	15.2269(12)
c (Å)	17.9241(15)
α (°)	99.829(3)
β (°)	94.467(3)
γ (°)	105.481(3)
V [$Å^3$]	2898.4(4)
Z	2
ρ [g·cm^{-3}]	1.175
μ [mm^{-1}]	0.076
F_{000}	1100
Crystal size [mm^3]	0.14 × 0.08 × 0.04

2Θ range (º)	4.008-56.8
h	-15 to 15
k	-20 to 20
l	-23 to 23
Reflections collected	108755
Independent reflections	14478
R(int)	0.0418
Refinement method	Full-matrix least-squares on F2
Parameters	697
Restraints	0
Goodness-of-fit on F^2	1.046
$R1$ [I > 2σ (I)]	0.0537
$wR2$ [I > 2σ (I)]	0.1386
$R1$ (all data)	0.0722
$wR2$ (all data)	0.1517
$\Delta\rho$ [e·Å^{-3}]	0.33/-0.45

Table S6. Hydrogen bonds for rotaxane **5b** [Å and (°)].

D-H...A	d(D-H)	d(H...A)	d(D...A)	<(DHA)
N5-H(05A)...O2	0.88(2)	2.16 (3)	3.026(2)	166 (2)
N6-H(06)...O2	0.91(2)	2.25 (2)	3.148(2)	167 (2)

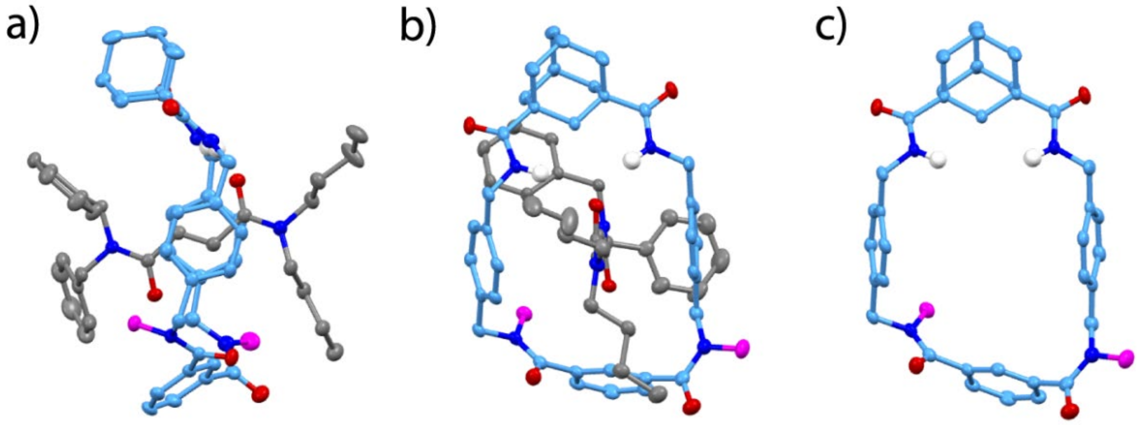

Figure S5. Molecular structure of **5b** with thermal ellipsoids drawn at 50% probability: a) lateral view; b) front view; c) front view after manually deleting the embedded thread. For clarity, selected hydrogens atoms have been deleted.

Rotaxane (*R*$_{mp}$, 1^5*S*, 1^7*R*, 3*R*, 4*S*)-7b

Single crystals of $C_{63.5}H_{75.5}Cl_{1.5}N_6O_{6.5}$ [AMC_VII_062_0mspp1_a] were by slow diffusion of pentane over a solution of the titled compound in $CHCl_3$. Intensities were registered at low temperature (100.0 K) on a Bruker D8 QUEST system equipped with a multilayer monochromator and a Mo K/a Incoatec microfocus sealed tube (λ = 0.71073 Å). Absorption corrections were based on multi-scans (program SADABS). Using Olex2,[7] the structure was solved with the SHELXT[8] structure solution program using Intrinsic Phasing and refined with the SHELXL[9] refinement package using Least Squares minimisation. Hydrogen atoms were included using a riding model. Two molecules of the rotaxane are included in the unit cell, one of them with disorder, which was modelled, originated due to the flexibility of the butyl groups at the thread. Moreover, one molecule of water and one molecule of $CHCl_3$ are also included. The structure was deposited with deposition number CCDC 2299444.

Table S7. Crystal data and structure refinement for **(*R*$_{mp}$, 1^5*S*, 1^7*R*, 3*R*, 4*S*)-7b**

Empirical formula	$C_{63.5}H_{75.5}Cl_{1.5}N_6O_{6.5}$
Formula weight	1079.97
T [K]	100
Wavelength [Å]	0.71073
Crystal system	Triclinic
Space group	P1
a (Å)	12.7358(4)
b (Å)	14.2956(5)
c (Å)	17.9078(6)
α (°)	89.4710(10)
β (°)	71.6740(10)
γ (°)	66.3290(10)
V [Å^3]	2808.86(16)
Z	2
ρ [g·cm^{-3}]	1.277
μ [mm^{-1}]	0.151
F_{000}	1152
Crystal size [mm^3]	0.29 × 0.23 × 0.2
2Θ range (º)	3.678-56.908

h	-17 to 16
k	-19 to 19
l	-23 to 23
Reflections collected	197271
Independent reflections	28077
R(int)	0.0273
Refinement method	Full-matrix least-squares on F2
Parameters	1443
Restraints	3
Goodness-of-fit on F^2	1.052
*R*1 [I > 2σ (I)]	0.0649
*wR*2 [I > 2σ (I)]	0.1871
*R*1 (all data)	0.0686
*wR*2 (all data)	0.1936
Δ*ρ* [e·Å^{-3}]	1.74/-1.53
Flack Parameter	0.133(8)

Table S8. Hydrogen bonds for rotaxane **(*R*$_{mp}$, 1^5*S*, 1^7*R*, 3*R*, 4*S*)-7b** [Å and (°)].

**D-H...A**	**d(D-H)**	**d(H...A)**	**d(D...A)**	**<(DHA)**
O13-H(13A)…Cl1	0.87	1.88	2.607(14)	139.8
O13-H(13B)…O10	0.87	2.18	3.008(13)	159.1
N4-H(4)…O1	0.88	2.12	2.818(4)	135.7
N5-H(5)…O2	0.88	2.26	3.096(4)	158.1
N6-H(6)…O2	0.88	2.36	3.230(4)	171.1
N10-H(10)…O7	0.88	2.08	2.854(5)	145.5
N11-H(11)…O8	0.88	2.34	3.216(4)	171.9
N12-H(12)…O8	0.88	2.20	3.071(4)	169.4

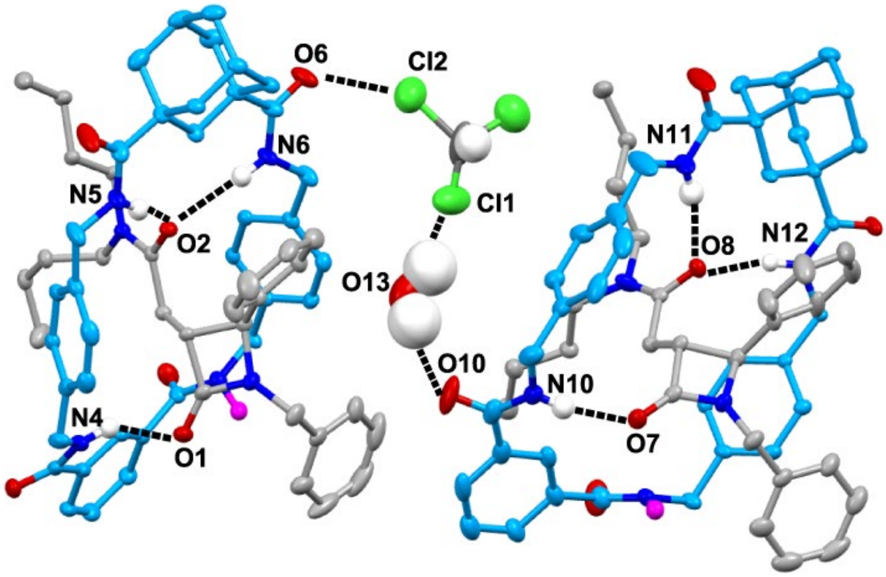

Figure S6. Molecular structure of **(*R*$_{mp}$, 1^{5}*S*, 1^{7}*R*, 3*R*, 4*S*)-7b (molecule A+B)** with thermal ellipsoids drawn at 50% probability, showing the non-covalent interactions between the two molecules and the solvent molecules (H_2O and $CHCl_3$). For clarity, selected hydrogens atoms have been deleted.

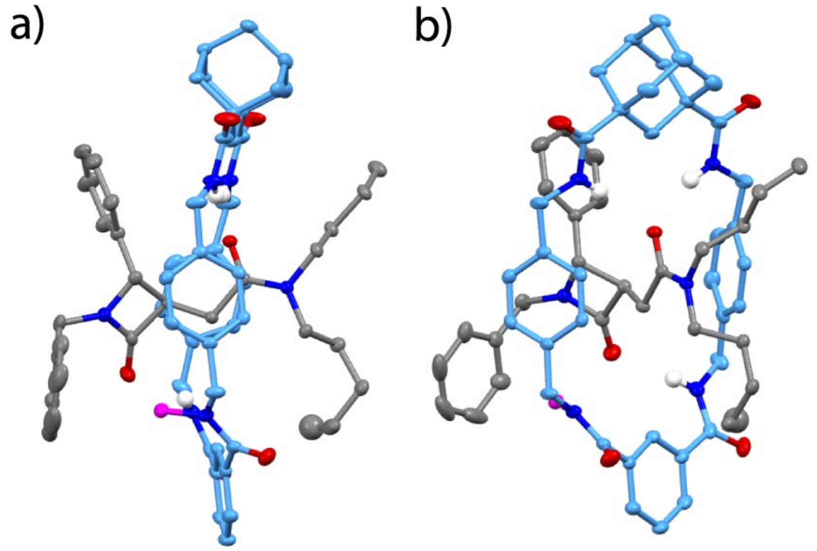

Figure S7. Molecular structure of **(*R*$_{mp}$, 1^{5}*S*, 1^{7}*R*, 3*R*, 4*S*)-7b (molecule A)** with thermal ellipsoids drawn at 50% probability: a) lateral view; b) front view. For clarity, selected hydrogens atoms and solvent molecules have been deleted.

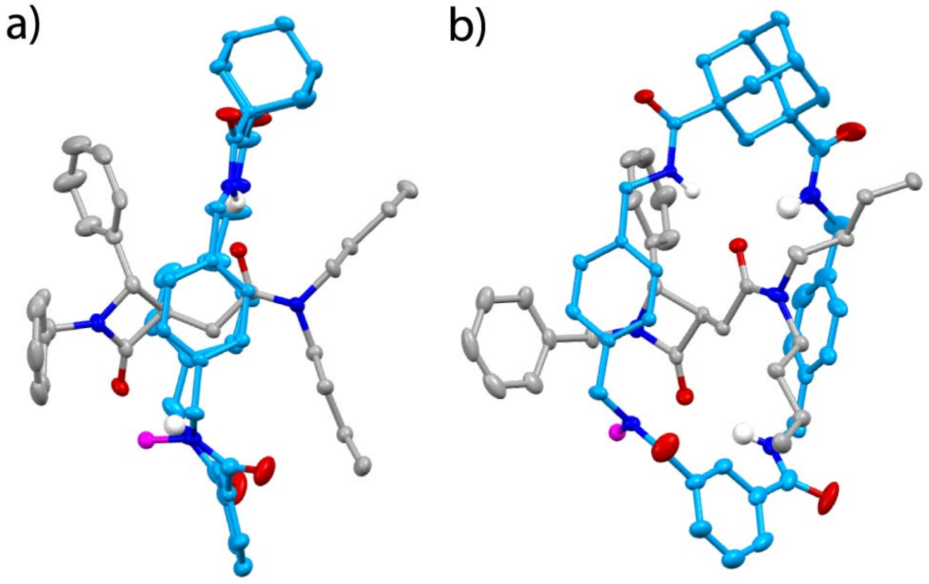

Figure S8. Molecular structure of **(*R*$_{mp}$, 1^5*S*, 1^7*R*, 3*R*, 4*S*)-7b (molecule B)** with thermal ellipsoids drawn at 50% probability: a) lateral view; b) front view. For clarity, selected hydrogens atoms and solvent molecules have been deleted. This molecule showed disorder in one butyl group, which was modelled (for clarity was deleted in this figure).

12. Determination of the absolute configuration.

The absolute configuration of all synthesized compounds were elucidated by means of single-crystal X-ray difraction technique and by the comparison with reported HPLC and specific rotation ([α]) values (Scheme S5).[10] The structure of the enantiopure rotaxane **1b** (second eluting enantiomer; t_R: 21.8 min) was elucidated by SCXRD, and its absolute configuration determined by anomalous dispersion characterized by the Flack parameter, being the ***(R*$_{mp}$*,* 1^5*S*, 1^7*R*)-1b**. The base-triggered cyclization of ***(R*$_{mp}$*,* 1^5*S*, 1^7*R*)-1b** at -20 ºC yielded two diastereosiomers: **(*R*$_{mp}$, 1^5*S*, 1^7*R*, 3*R*, 4*S*)-7b (major)** and **(*R*$_{mp}$, 1^5*S*, 1^7*R*, 3*S*, 4*R*)-7b (minor)**, in a 9:1 ratio aprox. The structure and the absolute configuration of the major diastereoisomer **(*R*$_{mp}$, 1^5*S*, 1^7*R*, 3*R*, 4*S*)-7b** was elucidated by SCXRD (the crystal used for the SCXRD was injected in the HPLC system, see Figure S9). Finally, the lactam **2b**, obtained after thermal dethreading of **(*R*$_{mp}$, 1^5*S*, 1^7*R*, 3*R*, 4*S*)-7b**, has a specific rotation of $[\alpha]_D^{25}$ = - 21, while the reported one **(3*S*,4*R*)-2b** had a value of + 20. While the main enantiomer **(3*S*,4*R*)-2b** (previously reported) was the second eluting enantiomer (same HPLC column and solvent system was employed: Chiralpak IC3 column, hex:IPA solvent system), in this work, the main enantiomer of lactam **2b** was eluting the first.

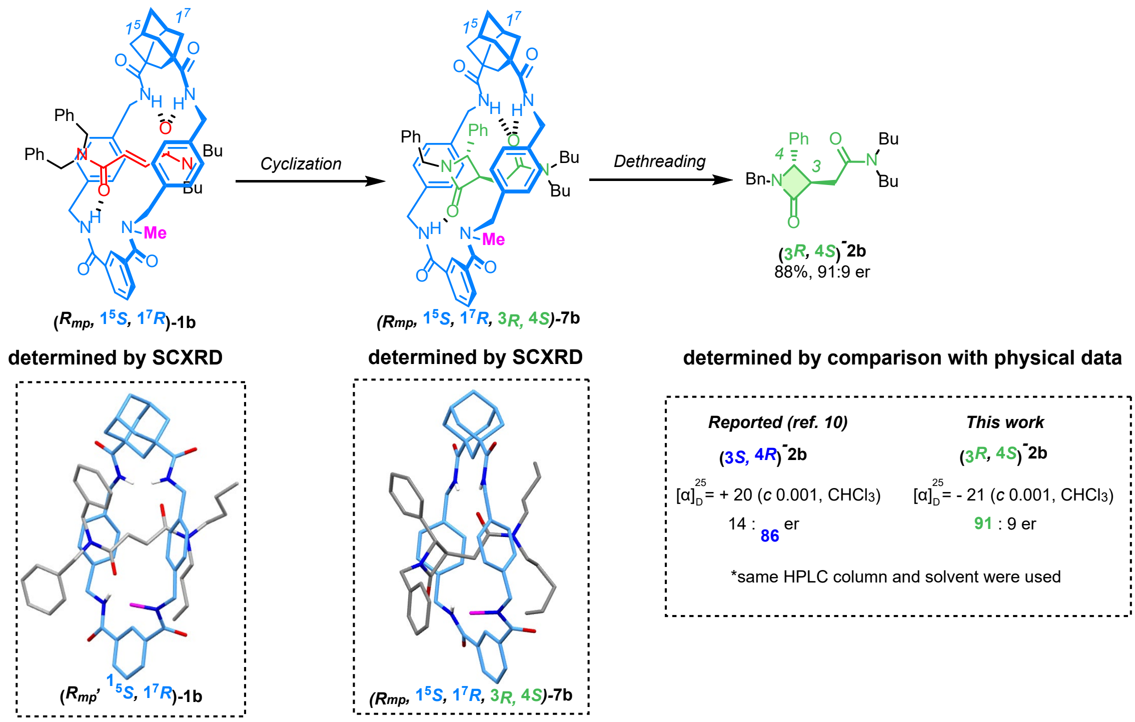

Scheme S5. Determination of the absolute configuration starting from enantiopure ***(R*****mp*****,*** **1**^{5}***S*****,** **1**^{7}***R*****)-1b** (second eluting enantiomer).

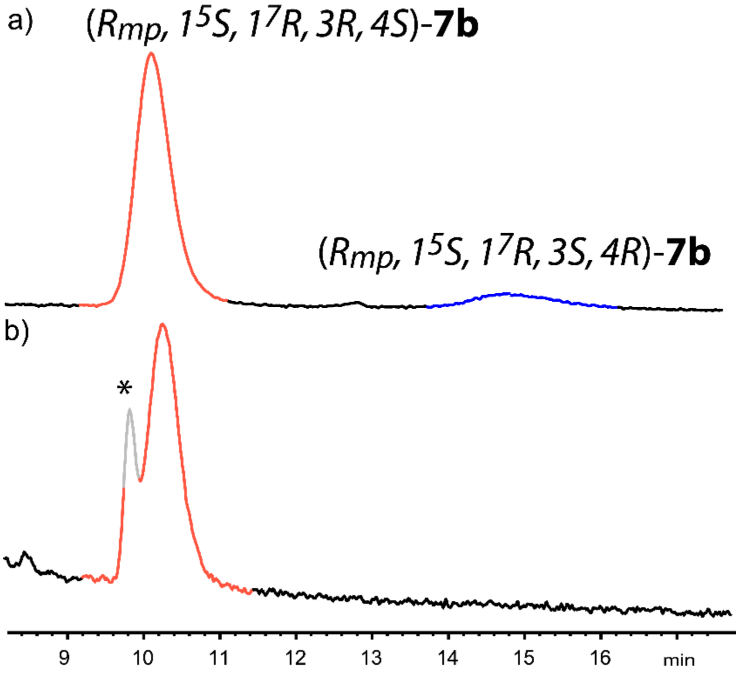

Figure S9. HPLC chromatogram of: a) the mixture of diastereosiomers **7b** obtained from the cyclization of ***(R*****mp*****,*** **1**^{5}***S*****,** **1**^{7}***R*****)-1b**; b) crystal of ***(R*****mp*****,*** **1**^{5}***S*****,** **1**^{7}***R*****, 3*****R*****, 4*****S*****)-7b** measured by SCXRD (impurity marked with an asterisk). HPLC conditions: Chiralpak IC-3 column, CH_2Cl_2:MeCN:IPA (85:13:2), 0.5 mL min^{-1}, 254 nm.

13. Assignment of the mechanically planar chirality for rotaxane 1b.

For the assignment of the stereochemistry of rotaxane **1b**, we have applied the proposed method by Vögtle (Scheme S6):[11]

(i) Determine the atom of highest priority in the thread using the CIP rules: label "A".

The atom at the thread with highest priority is the oxygen atom closer to the benzyl groups.

(ii) Having A, determine the highest priority atom (using the CIP rules) that allows thread direction: label "B".

The atom with highest priority that allows thread direction is the nitrogen atom attached to the benzyl groups.

(iii) Repeat the same process for the macrocycle. Label the highest priority atom as "C" and label as "D" the highest priority atom found that allows macrocycle direction.

The atom at the macrocycle with highest priority is the oxygen atom of the isophthlamide unit closer to the methylated nitrogen.

The atom with highest priority that allows a macrocycle direction is the methylated nitrogen atom.

(iv) View the assembly along the direction A-B at the thread and observe the orientation of C-D at the macrocycle: clockwise (R_{mp}) or anticlockwise (S_{mp}).

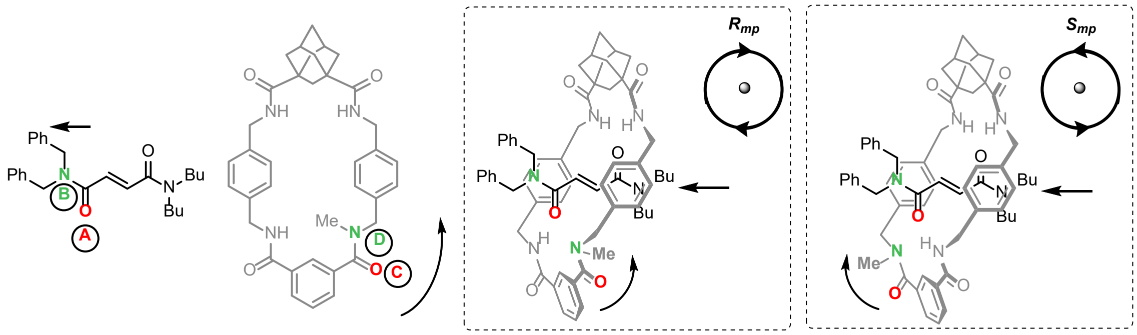

Scheme S6. Assignment of the mechanically planar chirality for **1b**.

14. Computational methods

The geometries of the molecules were optimized by using the wB97X-D[12] hybrid-functional with the def2-SVP basis sets.[13] The nature of minimum and transition structures of all stationary points on the potential energy surface was confirmed by frequency analysis at the same level of theory. The stability of the resulting wave functions was checked for all the optimized structures.[14] The computed thermochemical corrections at wB97X-D/def2-SVP level were combined with single-point energy calculations at the SMD(DMF)/r2-SCAN-3c[15]//wB97X-D/def2-SVP level to yield Gibbs free energies G at 298.15 K ($G_{298,sol}$). Solvent effects were calculated with the SMD solvation model with *N,N*-dimethylformamide parameters.[16] The ultrafine grid implemented in Gaussian 16 C. 01 was used.[17] Single point calculations were performed by using the program ORCA 5.0.[18]

Decomposition of the energy

The energy of **(3*S*, 4*R*)-TS*Rmp*** relative to **(3*R*, 4*S*)-TS*Rmp*** was decomposed as follows:

$$\Delta\Delta E^{\neq} = \Delta\Delta E_{int} + \Delta\Delta E_{thread} + \Delta\Delta E_{mac}$$

where $\Delta\Delta E_{thread}$ is the difference in the energy of the threads, and $\Delta\Delta E_{mac}$ is the difference between the macrocycles. The difference in interaction energies between thread and macrocycle is given by $\Delta\Delta E_{int}$. All these calculations were performed taking the geometry of the thread and macrocycle in their respective TS geometries.

Table S9. Electronic energies (E_{SCF},298,sol; in Hartree) computed at the SMD(DMF)/ r2SCAN-3c//wB97X-D/def2-SVP level of theory for the computed transition structures and their components. Energy differences are given in kJ mol^{-1}.

Structure	Thread+Macrocyle	Thread	Macrocycle	*E*int
**(3*S*, 4*R*)-TS*Rmp***	-2988,32756	-1034,59848	-1953,68426	-0,04482836
**(3*R*, 4*S*)-TS*Rmp***	-2988,33177	-1034,59820	-1953,68536	-0,04820841
	$\Delta\Delta E^{\neq}$	$\Delta\Delta E_{thread}$	$\Delta\Delta E_{mac}$	$\Delta\Delta E_{int}$
**Diff [(3*S*,4R)-TS*Rmp* - (3*R*,4S)-TS*Rmp*]**	+11.1	-0.72	+2.90	+8.87

Cartesian coordinates

(3*S*,4*R*)-TS$_{Rmp}$
SCF = -2985.84635244

-1 1

O	5.269617	-2.009238	2.211963
O	4.092816	1.884595	-3.818324
O	-5.639944	1.557977	-2.671402
N	3.172140	-1.677850	1.459180
H	2.537311	-1.294577	0.758322
N	2.380249	0.801164	-2.808958
H	2.077626	0.144597	-2.087178
N	-4.262288	1.599975	-0.874289
H	-3.867586	1.113107	-0.064603
C	-4.923736	-2.652921	-0.561223
C	-5.973744	-3.201763	-1.300951
H	-6.138436	-4.280879	-1.269584
C	-6.784141	-2.378736	-2.079147
H	-7.613071	-2.807649	-2.646973
C	-6.515380	-1.016218	-2.159866
H	-7.101132	-0.354708	-2.800356
C	-5.450551	-0.460025	-1.444174
C	-4.663366	-1.281765	-0.631720
H	-3.820471	-0.873648	-0.067307
C	-2.857379	-4.251866	2.190647
H	-2.839528	-5.199792	1.638631
C	-1.455049	-3.696595	2.293287
C	-1.020335	-3.040512	3.445856
H	-1.708350	-2.909093	4.286386
C	0.283435	-2.557494	3.539796
H	0.607655	-2.039713	4.446491
C	1.188798	-2.729829	2.490145
C	0.745193	-3.368869	1.326173
H	1.424007	-3.472838	0.478232
C	-0.560522	-3.835692	1.225210
H	-0.902891	-4.322482	0.308460
C	2.616480	-2.276911	2.651357
H	2.687017	-1.572917	3.495788
H	3.254990	-3.134626	2.921715
C	4.518630	-1.567554	1.352614
C	3.692357	1.123705	-2.949100
C	1.397180	1.244016	-3.769628
H	1.879444	2.036783	-4.361702
C	0.113150	1.744191	-3.150768
C	-1.114953	1.415113	-3.734310
H	-1.131465	0.792405	-4.633510
C	-2.313233	1.830492	-3.167618
H	-3.264900	1.543140	-3.621750
C	-2.317274	2.577321	-1.982397
C	-1.093855	2.951243	-1.429794
H	-1.085235	3.537402	-0.506242

C	0.108414	2.538463	-2.004691
H	1.057693	2.794706	-1.527876
C	-3.622337	2.845628	-1.260896
H	-3.439458	3.447124	-0.361179
H	-4.325625	3.389476	-1.906737
C	-5.139352	0.993783	-1.705694
O	-3.520670	-4.544102	-0.421992
N	-3.795534	-3.375650	1.503432
C	-4.015209	-3.598187	0.178593
O	1.182527	-1.330540	-0.977868
O	-2.931359	0.556730	1.509878
N	-0.420412	-1.738270	-2.529528
N	-0.990517	1.515867	2.294785
C	-1.589197	2.415472	3.263302
H	-0.798517	2.707424	3.971279
C	1.472827	1.674444	2.354731
C	2.652341	2.263517	1.836925
H	2.602030	2.786289	0.877082
C	3.855319	2.195876	2.524498
H	4.742484	2.672487	2.098289
C	3.943789	1.537503	3.755948
H	4.896058	1.471861	4.285297
C	2.790267	0.960491	4.287683
H	2.839059	0.440463	5.248979
C	1.578393	1.022668	3.605756
H	0.688477	0.544023	4.023715
C	-1.824142	-1.947045	-2.824816
H	-2.402781	-1.019147	-2.721933
H	-2.282647	-2.716757	-2.178413
C	0.535050	-2.487389	-3.309691
H	1.543965	-2.108190	-3.110615
H	0.312150	-2.374910	-4.382626
C	0.241151	1.719192	1.618430
H	0.189522	2.379042	0.750949
C	-0.697877	-0.095645	0.745716
H	0.069746	-0.645273	1.295768
C	-1.695600	0.640184	1.558934
C	-0.966022	-0.447765	-0.556569
C	-0.009651	-1.193430	-1.330816
H	-2.361601	1.861637	3.816647
H	0.526055	-3.567160	-3.067130
H	-1.921603	-2.274596	-3.869358
H	-1.848590	-0.037944	-1.045939
H	1.154621	0.425715	-4.471130
C	-2.195048	3.639236	2.614343
C	-3.573891	3.730741	2.403152
C	-1.370453	4.674786	2.158092
C	-4.122251	4.842549	1.764812

H	-4.215578	2.905606	2.720179
C	-1.915123	5.779474	1.505182
H	-0.290322	4.602707	2.311335
C	-3.293892	5.868438	1.311092
H	-5.200968	4.896656	1.600853
H	-1.259294	6.576248	1.146500
H	-3.721461	6.733522	0.799010
H	-3.254762	-4.446566	3.199747
C	4.685226	0.426068	-2.012575
C	4.042688	-0.204679	-0.769695
C	5.765649	1.428522	-1.569505
C	5.357770	-0.695095	-2.840362
H	3.527228	0.567776	-0.174929
H	3.287113	-0.938908	-1.077277
C	5.094182	-0.913707	0.092830
H	5.296002	2.232674	-0.977757
H	6.207655	1.898706	-2.460425
C	6.830527	0.711534	-0.729748
H	4.588597	-1.413347	-3.172777
H	5.809027	-0.254192	-3.743461
C	6.418002	-1.409128	-1.994410
C	6.180639	0.093606	0.513090
C	5.758649	-2.022803	-0.752907
H	7.596662	1.440069	-0.416515
C	7.487937	-0.396118	-1.564575
H	6.883247	-2.209302	-2.594013
H	6.924266	-0.424501	1.136791
H	5.724524	0.876113	1.137295
H	4.996203	-2.761839	-1.053278
H	6.501026	-2.549120	-0.132682
H	7.977106	0.036167	-2.454087
H	8.270297	-0.900674	-0.972877
C	-4.365249	-2.283398	2.272454
H	-3.710131	-1.398565	2.298513
H	-5.330662	-1.978555	1.850741
H	-4.542546	-2.634191	3.300839

(3*R*,4*S*)-TS*Rmp*
SCF = -2985.84866339

-1 1

O	-4.955105	2.836959	-2.084626
O	-4.425250	-1.395586	3.816369
O	4.875677	-0.722602	3.531277
N	-3.041169	2.684205	-0.884498
H	-2.502045	2.242690	-0.135087
N	-2.690153	-0.154877	3.052191
H	-2.313127	0.470024	2.334902
N	4.101228	-1.772656	1.687183
C	5.500775	2.027410	-0.214404
C	6.749679	2.445676	0.250240
H	7.217843	3.298697	-0.244139
C	7.355033	1.796776	1.322451
H	8.331002	2.129544	1.683308
C	6.705735	0.739171	1.950913
H	7.142960	0.249347	2.822862
C	5.460158	0.298234	1.490646
C	4.860376	0.949768	0.407749
H	3.872337	0.635966	0.064391
C	3.142320	2.877622	-3.061491
H	3.642526	3.837200	-3.257984
H	3.190484	2.275675	-3.983604
C	1.695821	3.099369	-2.677604
C	0.647863	2.605755	-3.456846
H	0.873584	2.032870	-4.360804
C	-0.678743	2.827578	-3.092036
H	-1.490232	2.431889	-3.709026
C	-0.987834	3.550468	-1.937459
C	0.062931	4.019359	-1.144044
H	-0.163401	4.563730	-0.223348
C	1.386139	3.797066	-1.507210
H	2.197034	4.168538	-0.874988
C	-2.421310	3.809870	-1.547243
H	-3.033991	4.032100	-2.431501
C	-4.316574	2.322276	-1.176520
C	-3.957115	-0.640539	2.976419
C	-1.813558	-0.506305	4.136708
H	-1.631067	0.368811	4.785373
H	-2.355354	-1.246079	4.746795
C	-0.482116	-1.072782	3.695322
C	0.635206	-0.932035	4.524552
H	0.540261	-0.379717	5.464496
C	1.866565	-1.472049	4.169169
H	2.741066	-1.328498	4.806861
C	2.012268	-2.168977	2.964818
C	0.897691	-2.306766	2.135028
H	0.997172	-2.834634	1.182006
C	-0.334265	-1.762660	2.490096
H	-1.183707	-1.851852	1.809209
C	3.364021	-2.699268	2.545208
H	3.977111	-2.902833	3.433546
C	4.783914	-0.772832	2.310537
O	5.347587	3.914883	-1.650927
N	3.881063	2.220638	-2.010748
H	3.512232	1.296697	-1.760637
C	4.899292	2.813944	-1.357672
O	-1.195911	1.650934	1.200495
O	2.460563	-0.121565	-1.959954
N	0.658796	2.027938	2.448493
N	0.545678	-1.319446	-2.367316
C	1.085310	-2.139605	-3.438107
H	0.232475	-2.563228	-3.989992
H	1.647862	-1.486184	-4.120207

C	-1.758046	-2.031511	-1.993742
C	-2.620356	-2.963832	-1.370115
H	-2.298053	-3.438218	-0.438145
C	-3.844585	-3.306124	-1.930827
H	-4.481662	-4.034627	-1.421485
C	-4.267903	-2.730628	-3.132376
H	-5.232132	-2.999896	-3.568690
C	-3.443450	-1.789534	-3.751876
H	-3.767571	-1.309366	-4.679133
C	-2.218172	-1.438853	-3.194284
H	-1.588735	-0.689917	-3.682432
C	2.089380	1.939794	2.653744
H	2.639708	2.197275	1.736839
H	2.381966	2.660814	3.429116
C	-0.115936	2.697602	3.461563
H	-1.173884	2.654985	3.180306
H	0.017501	2.211517	4.443635
C	-0.456496	-1.722325	-1.451798
H	-0.115911	-2.350554	-0.626640
C	0.359956	0.242298	-0.746234
H	-0.583445	0.623988	-1.144209
C	1.262049	-0.353027	-1.761101
C	0.834688	0.670900	0.468102
C	0.031891	1.464508	1.363808
H	0.179648	3.757083	3.561158
H	2.411138	0.938894	2.988783
H	1.825008	0.345461	0.785308
H	-2.459411	4.699603	-0.892905
C	1.980142	-3.244262	-2.922250
C	3.371857	-3.140739	-3.006074
C	1.422050	-4.368742	-2.301687
C	4.191380	-4.148881	-2.500353
H	3.810971	-2.247117	-3.455165
C	2.240292	-5.369235	-1.778621
H	0.334416	-4.453543	-2.229487
C	3.627953	-5.264978	-1.882054
H	5.277435	-4.054651	-2.575942
H	1.791088	-6.239863	-1.294731
H	4.269429	-6.052555	-1.479175
C	-4.965350	1.252774	-0.291524
C	-4.090001	0.834791	0.891864
C	-5.266762	-0.000625	-1.135466
C	-6.293446	1.829007	0.242424
H	-3.147542	0.412941	0.511800
H	-3.831110	1.720944	1.493594
C	-4.795097	-0.200589	1.773181
H	-4.324231	-0.419780	-1.519799
H	-5.877737	0.284145	-2.006215
C	-5.991757	-1.043554	-0.274754
H	-6.088035	2.733590	0.841087
H	-6.914002	2.139673	-0.611275
C	-7.012616	0.782032	1.100568
C	-5.107718	-1.442499	0.913560
C	-6.123091	0.390386	2.287101
H	-6.192347	-1.934585	-0.890201
C	-7.310921	-0.458655	0.247237
H	-7.957037	1.207580	1.479216
H	-5.604853	-2.199117	1.540800
H	-4.166288	-1.879220	0.545998
H	-5.915239	1.275942	2.912618
H	-6.618731	-0.352555	2.930213
H	-7.965656	-0.186008	-0.597744
H	-7.848526	-1.211325	0.848876
H	3.242702	-3.637218	1.984604
C	4.044729	-2.004997	0.255310
H	3.137925	-1.594523	-0.212258
H	4.910058	-1.560227	-0.249377
H	4.066038	-3.087265	0.065132

15. ^{1}H and ^{13}C NMR Spectra of synthesized compounds

4b (^{1}H NMR, 400 MHz, $CDCl_3$, 298K)

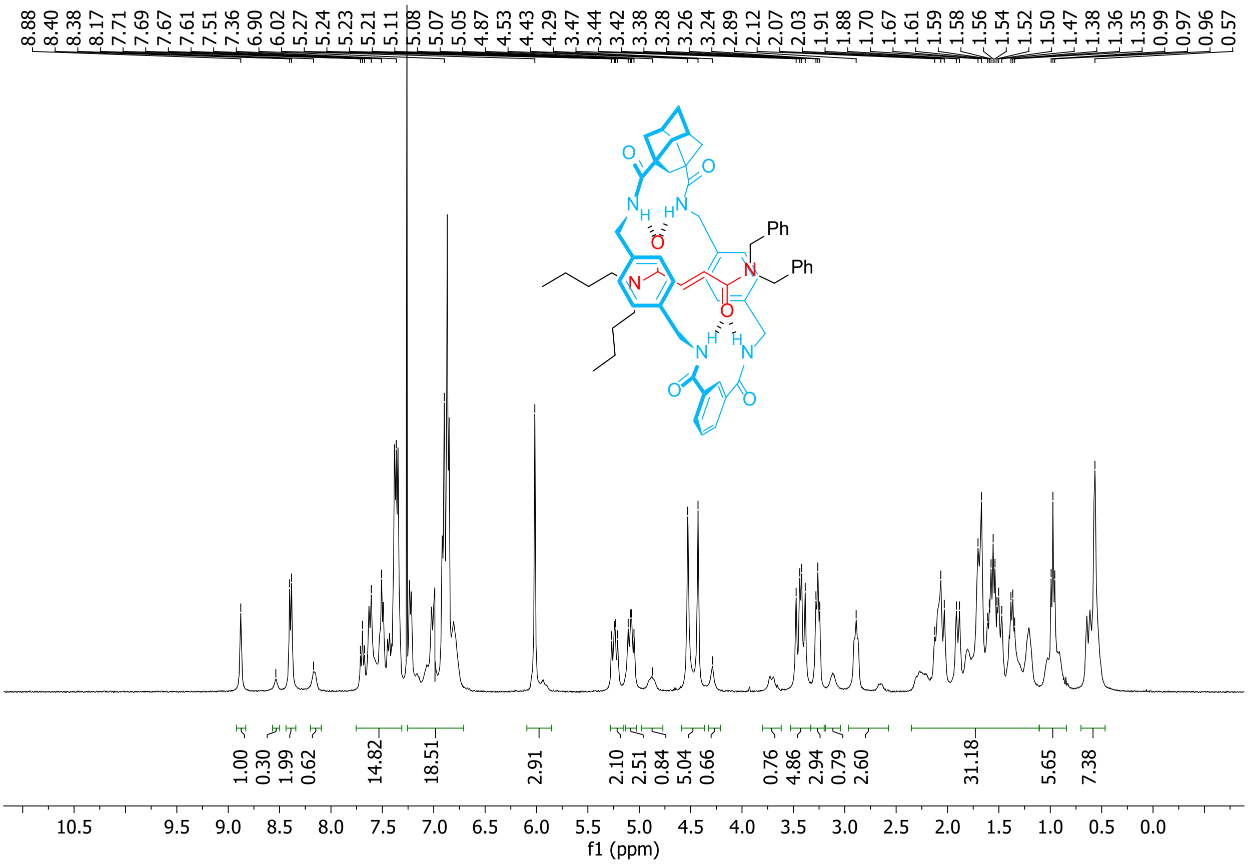

4b (^{13}C NMR, 101 MHz, $CDCl_3$, 298K)

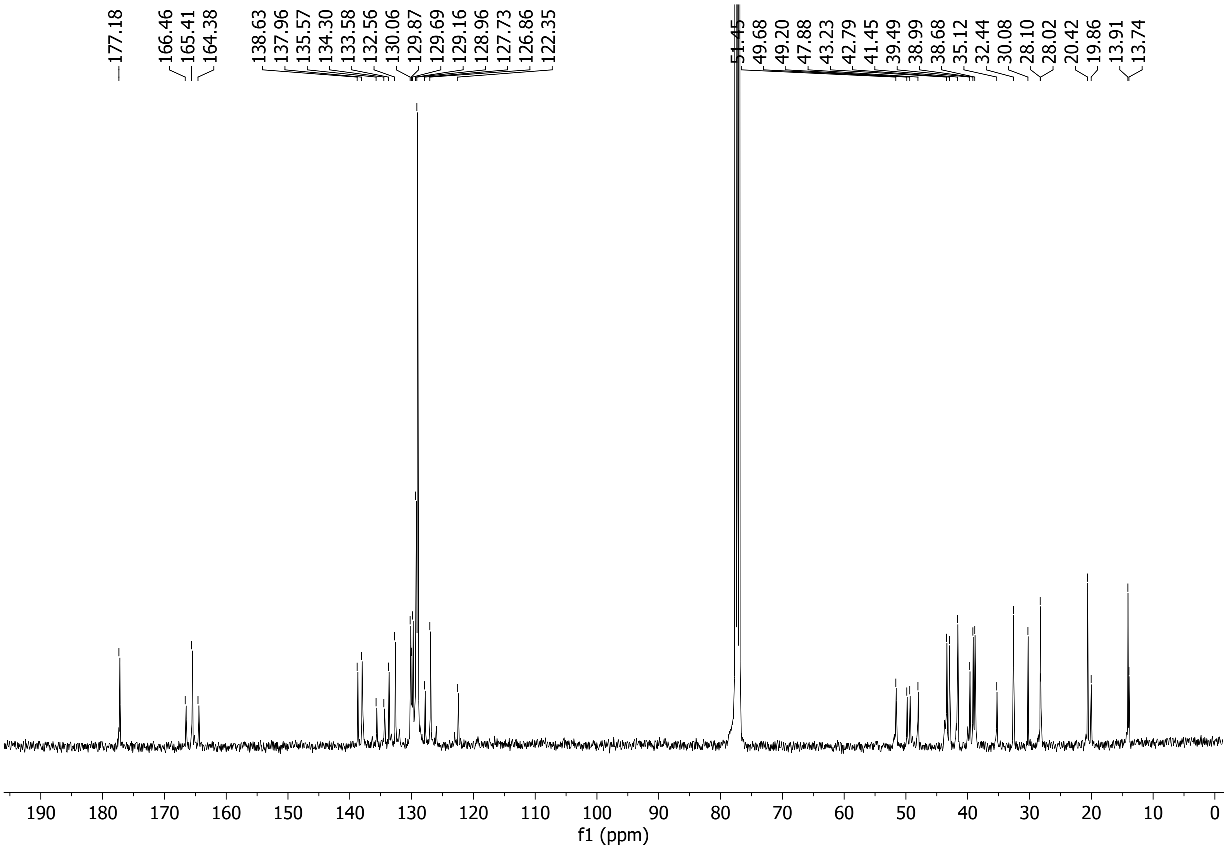

4b (COSY, 400 MHz, $CDCl_3$, 298K)

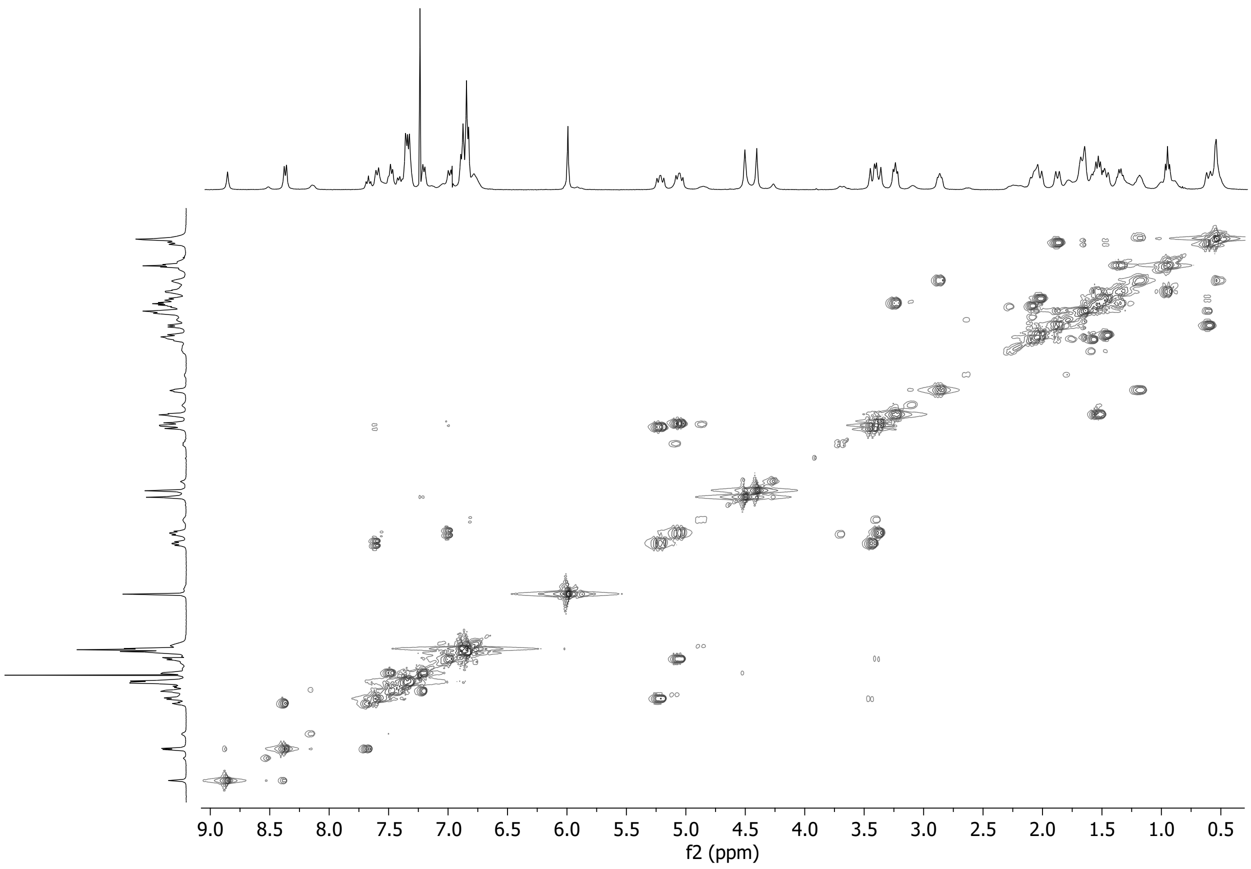

4c (^{1}H NMR, 600 MHz, $CDCl_3$, 298K)

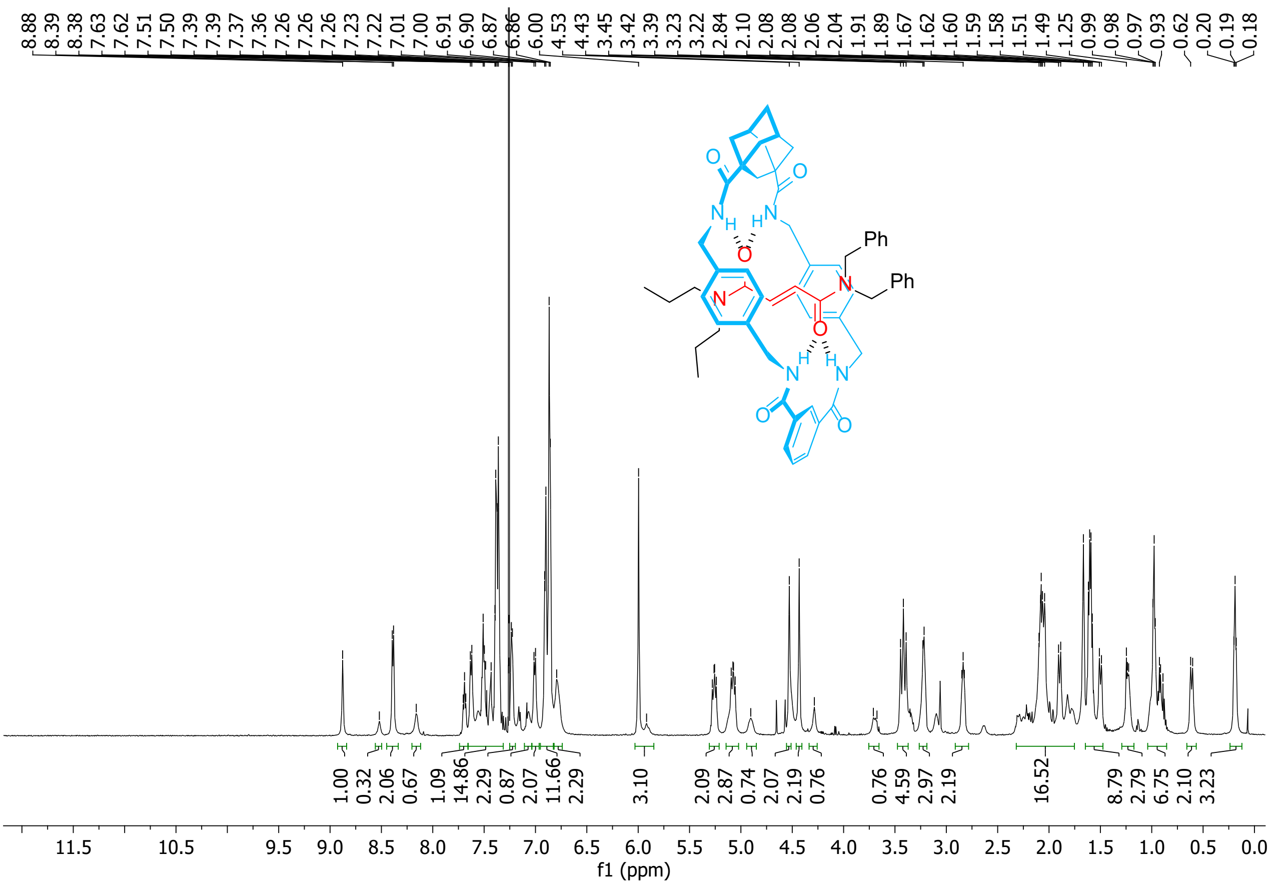

4c (^{13}C NMR, 150 MHz, $CDCl_3$, 298K)

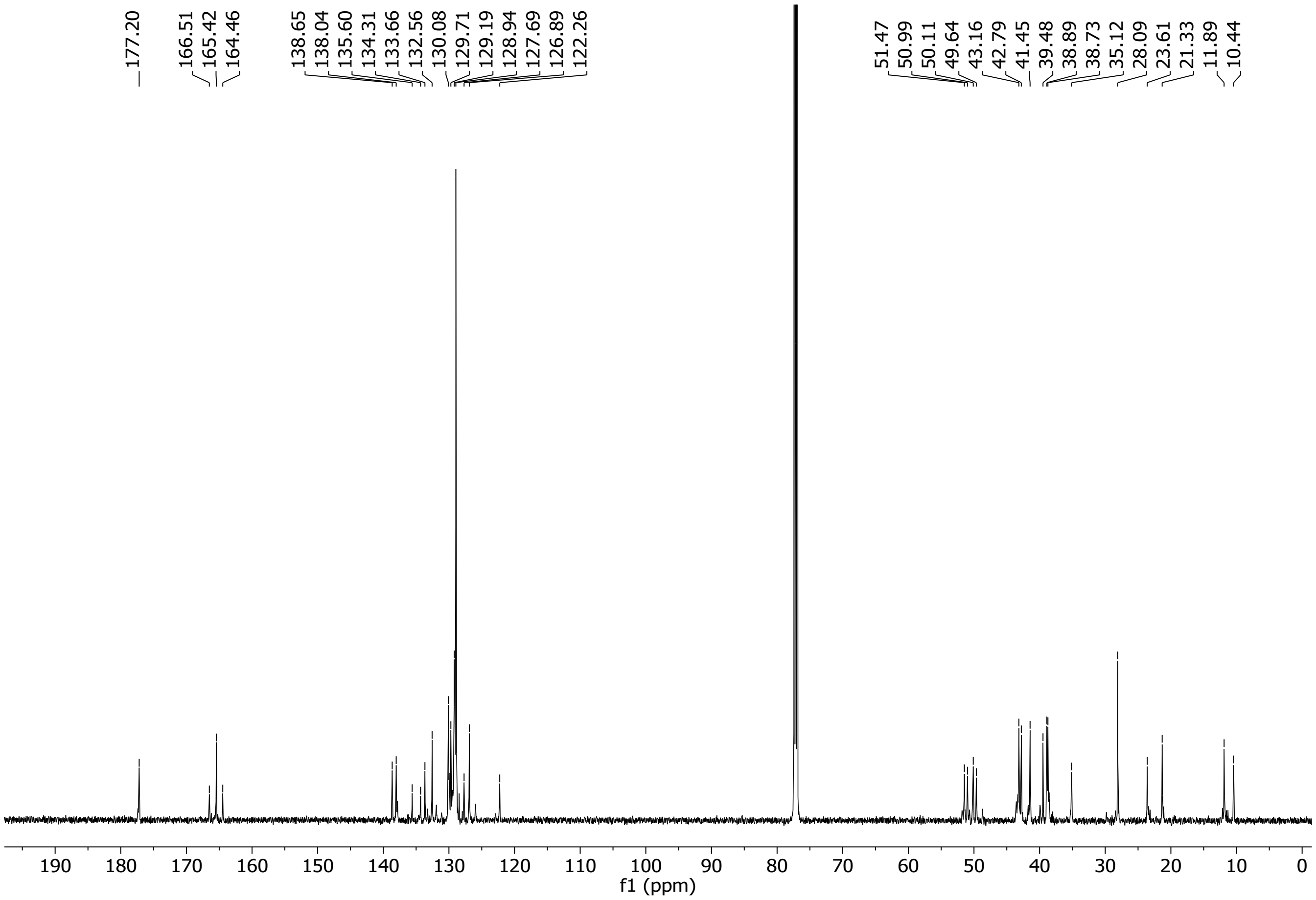

4c (COSY, 600 MHz, CDCl3, 298K)

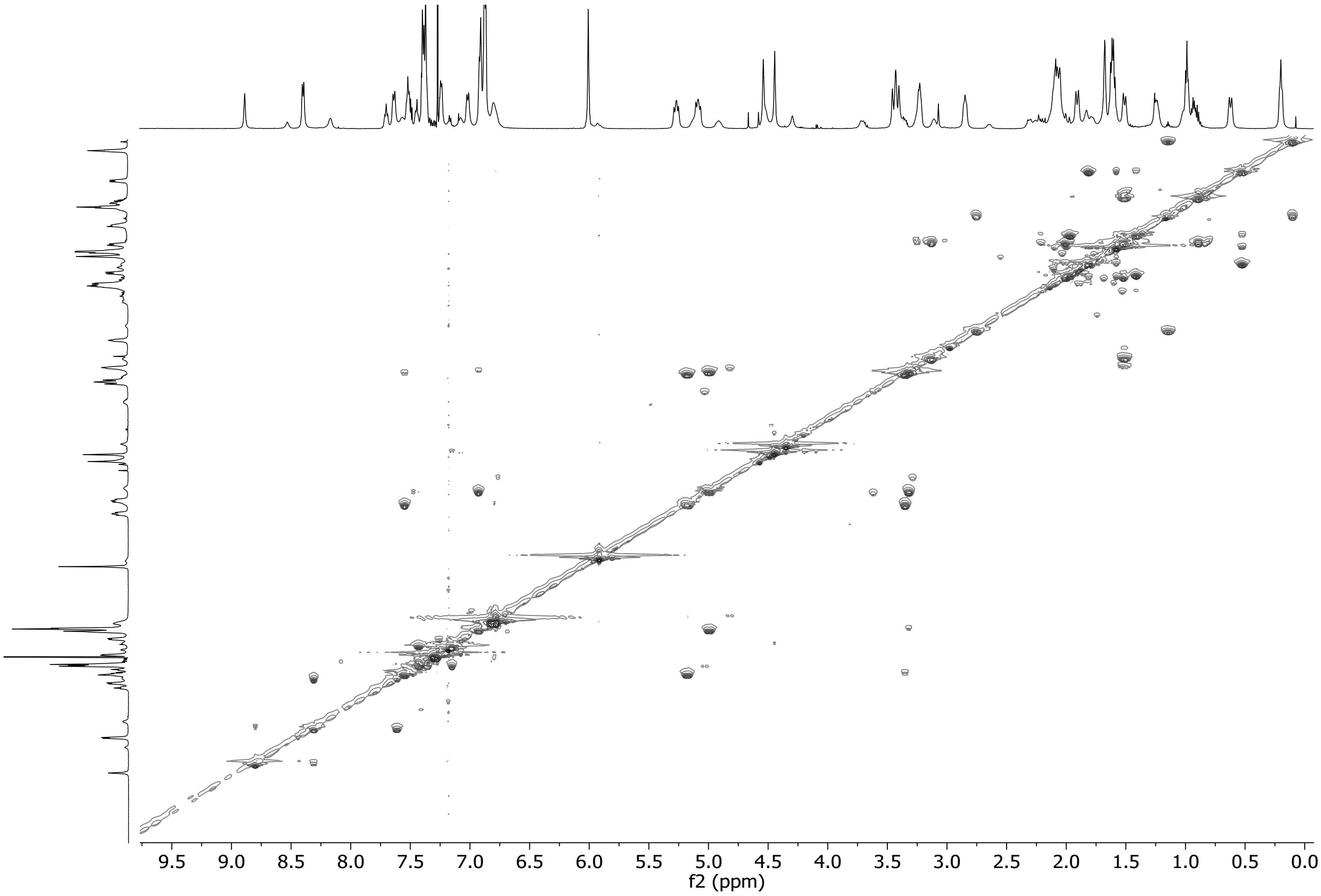

6a (^{1}H NMR, 400 MHz, $C_2D_2Cl_4$, 373K)

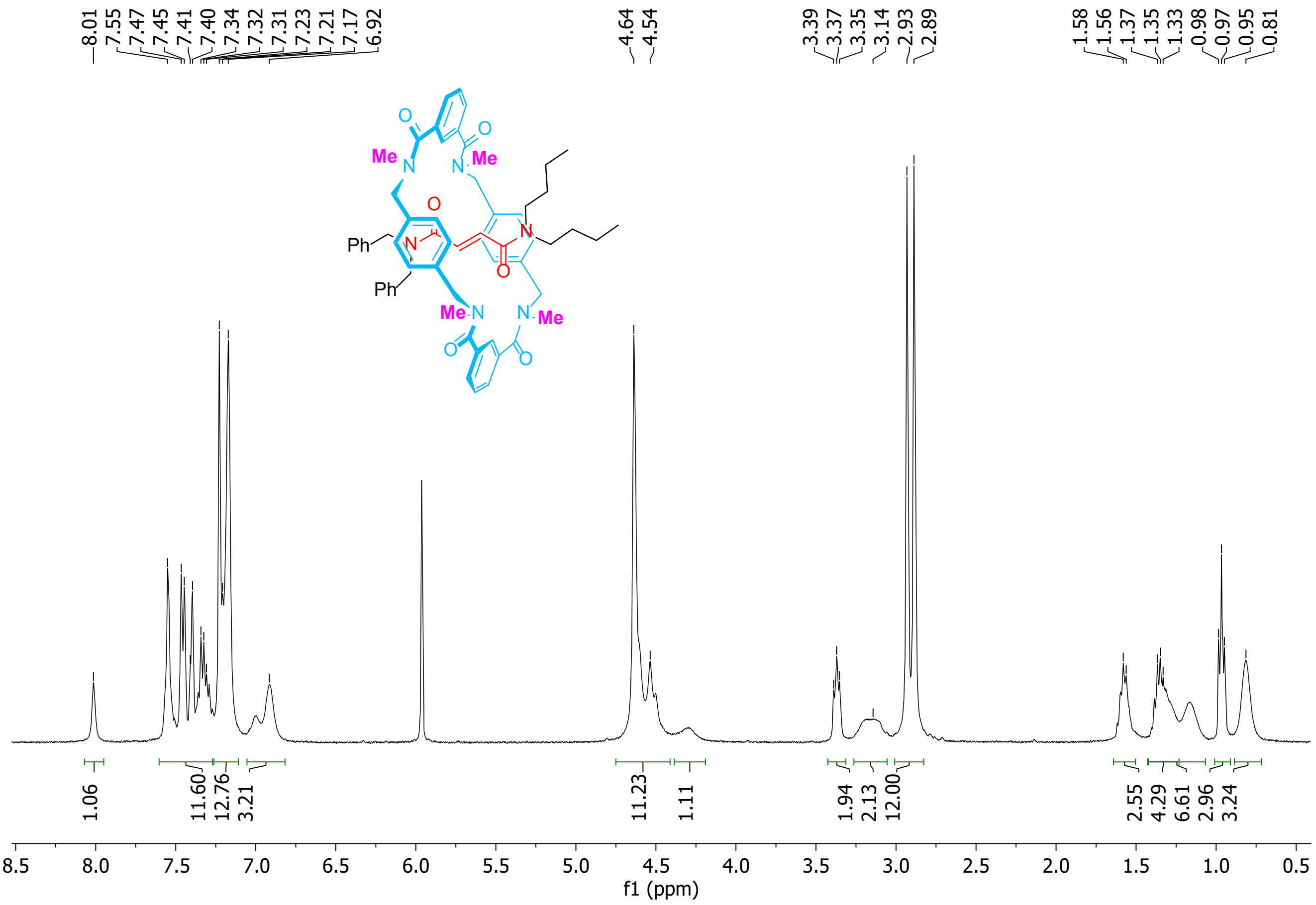

6a (^{13}C NMR, 101 MHz, $C_2D_2Cl_4$, 373K)

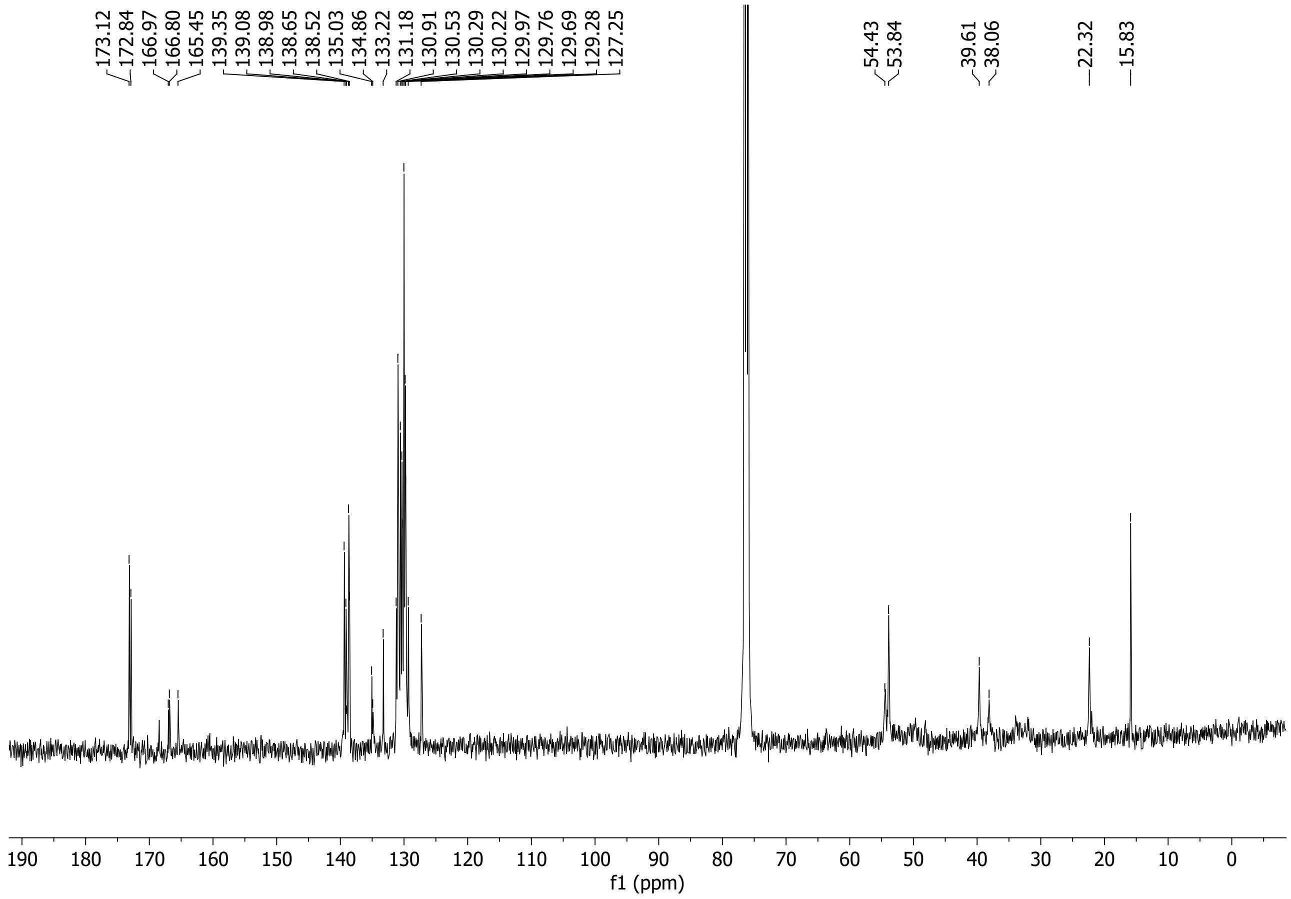

***rac*-1b** (^{1}H NMR, 400 MHz, $CDCl_3$, 318K)

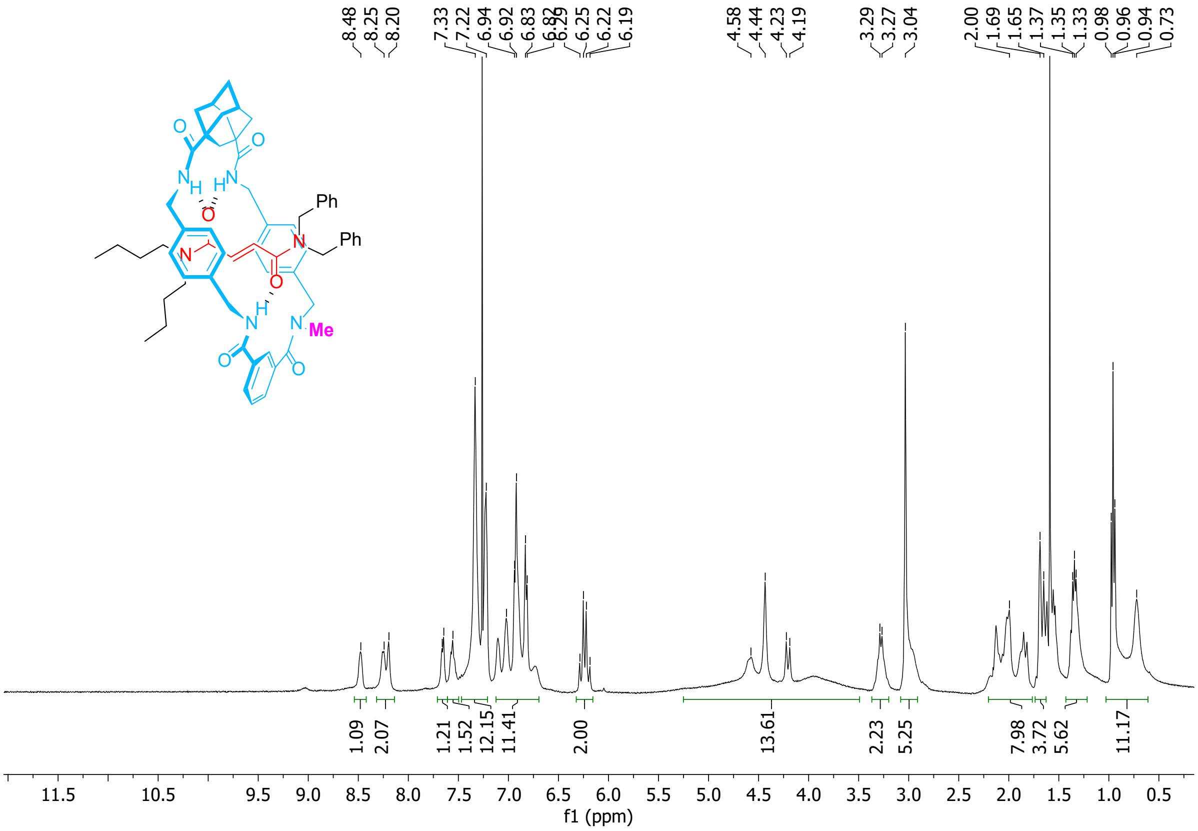

***rac*-1b** (^{13}C NMR, 101 MHz, $CDCl_3$, 318K)

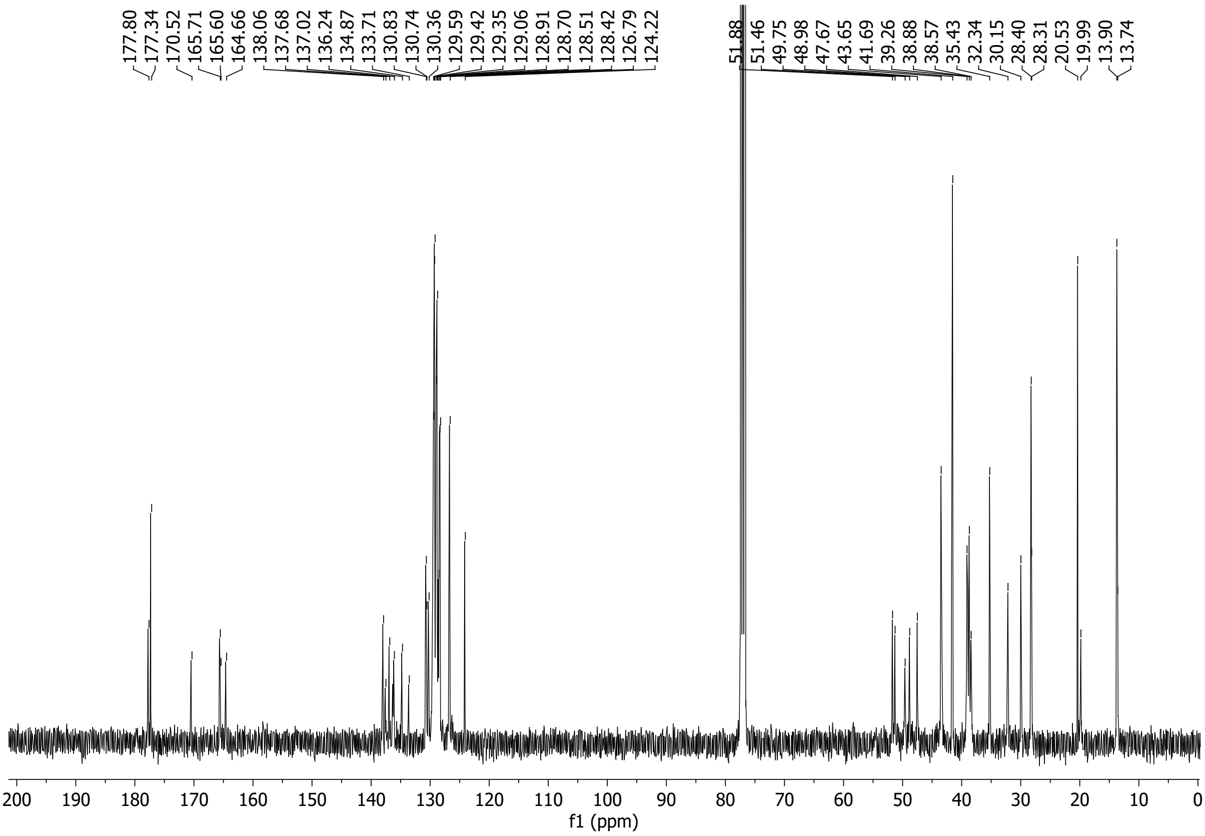

rac-1b (^{135}DEPT NMR, 101 MHz, $CDCl_3$, 318K)

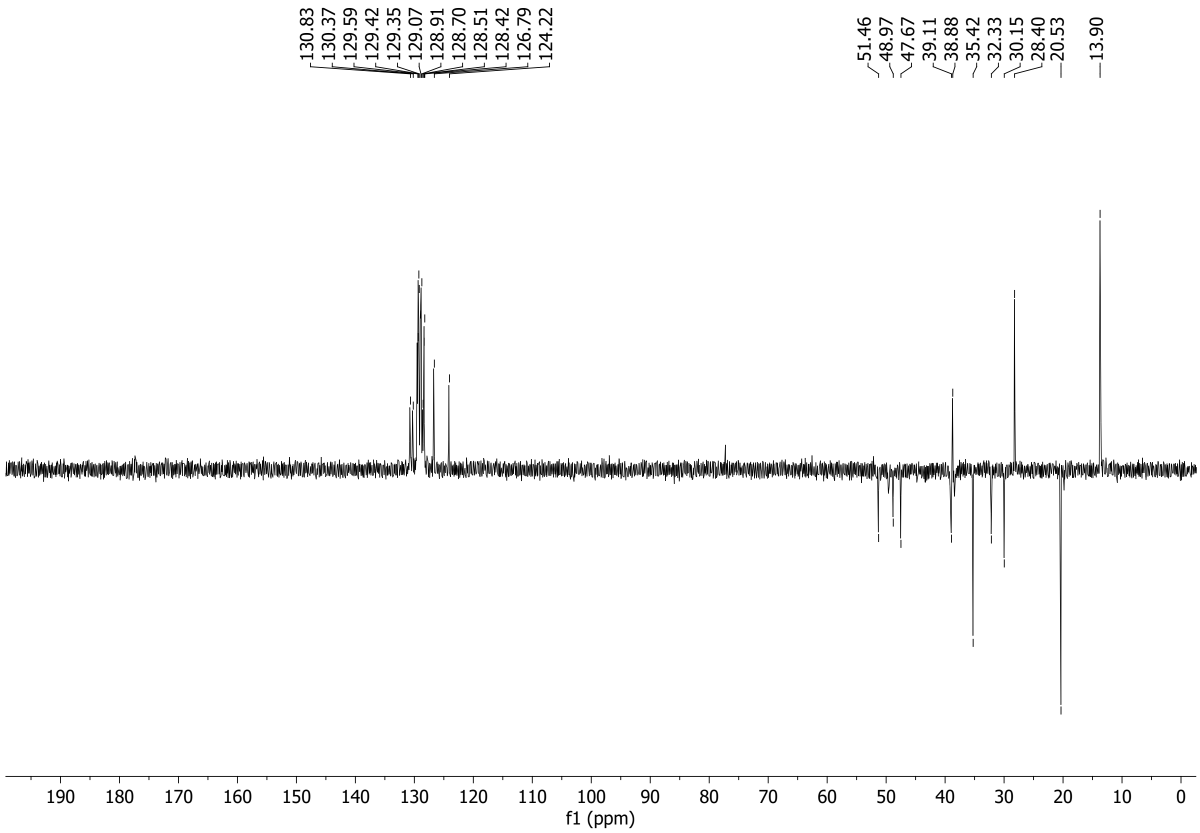

rac-1b (COSY, 400 MHz, $CDCl_3$, 318K)

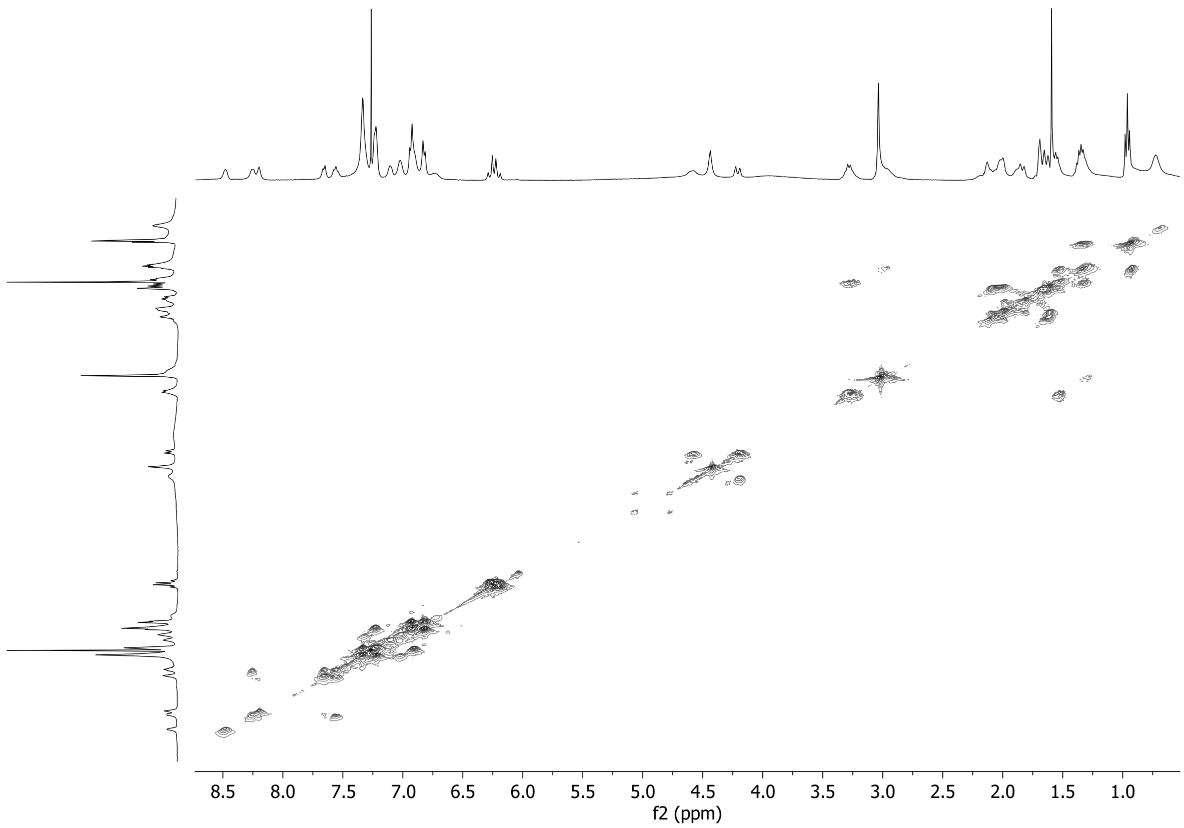

***rac*-1b** (HMQC, CDCl3, 318K)

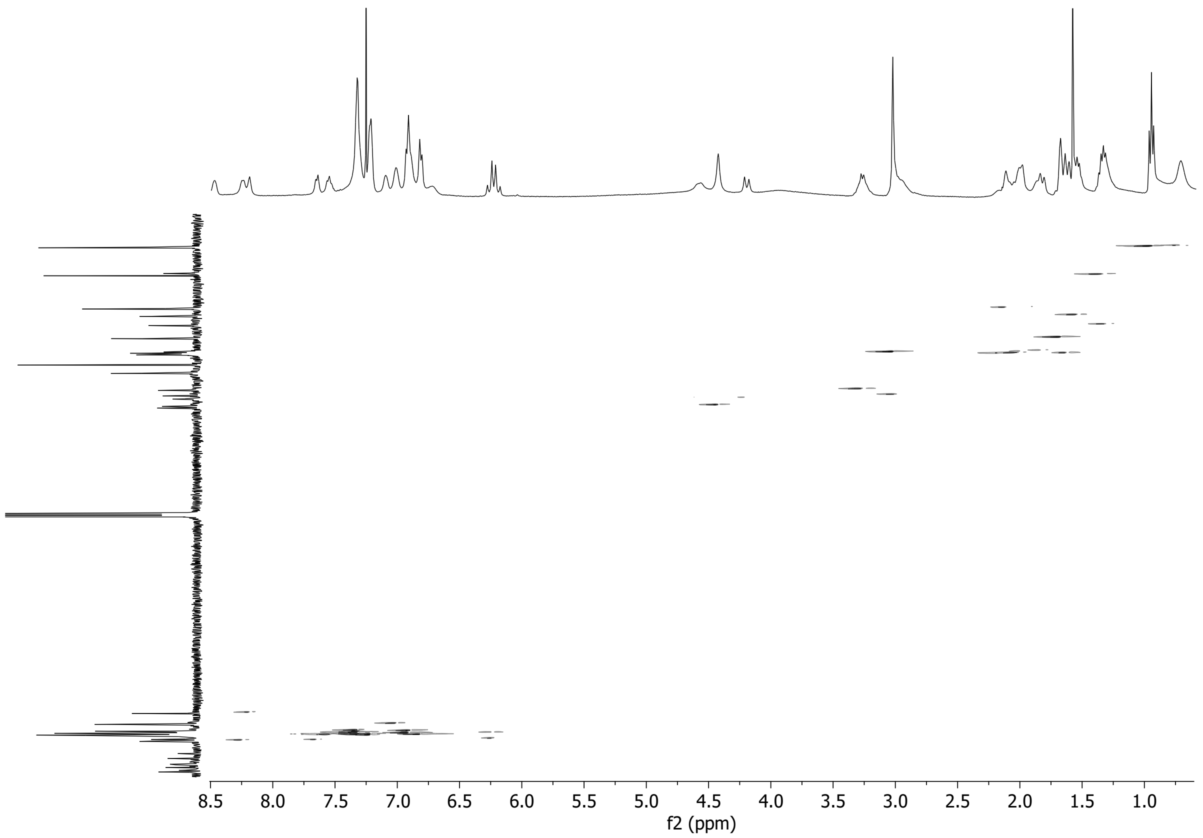

***rac*-1b** (HMBC, CDCl3, 318K)

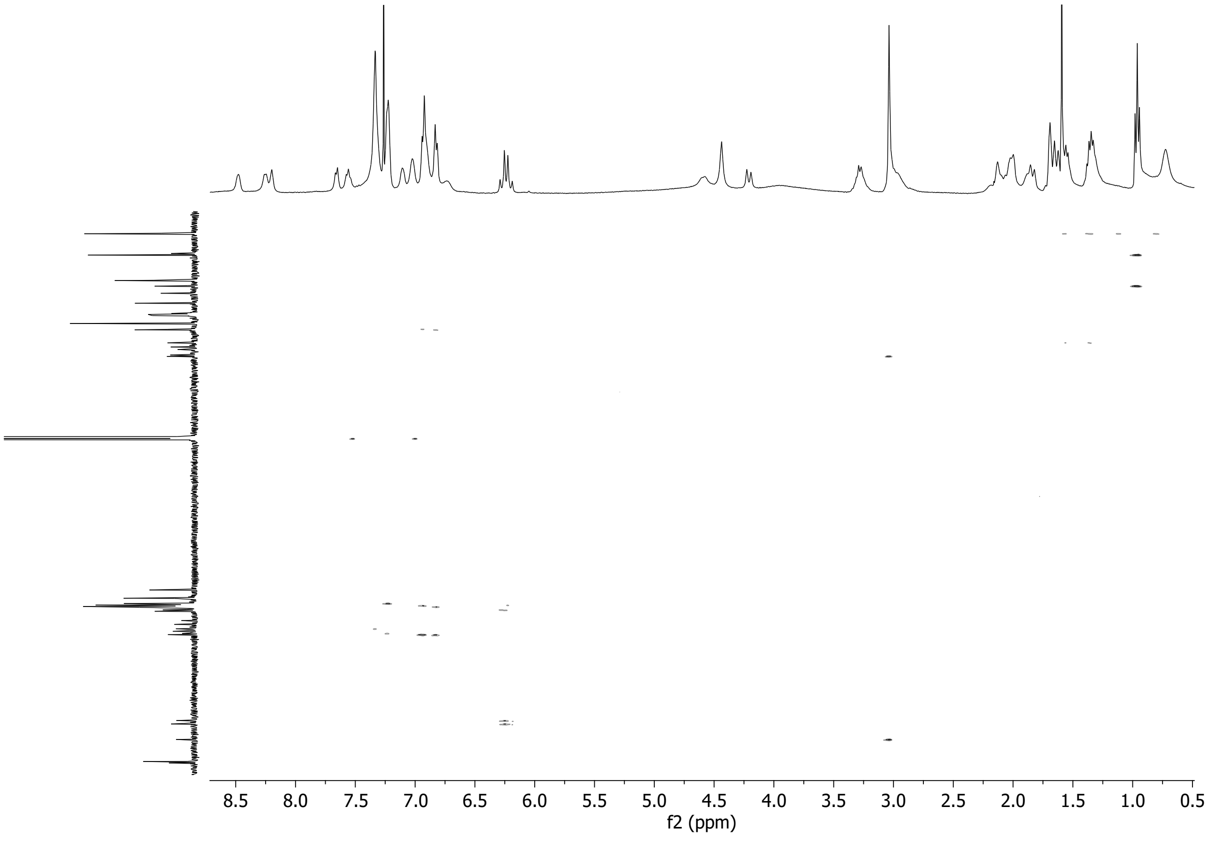

5b (^{1}H NMR, 400 MHz, $CDCl_3$, 318K)

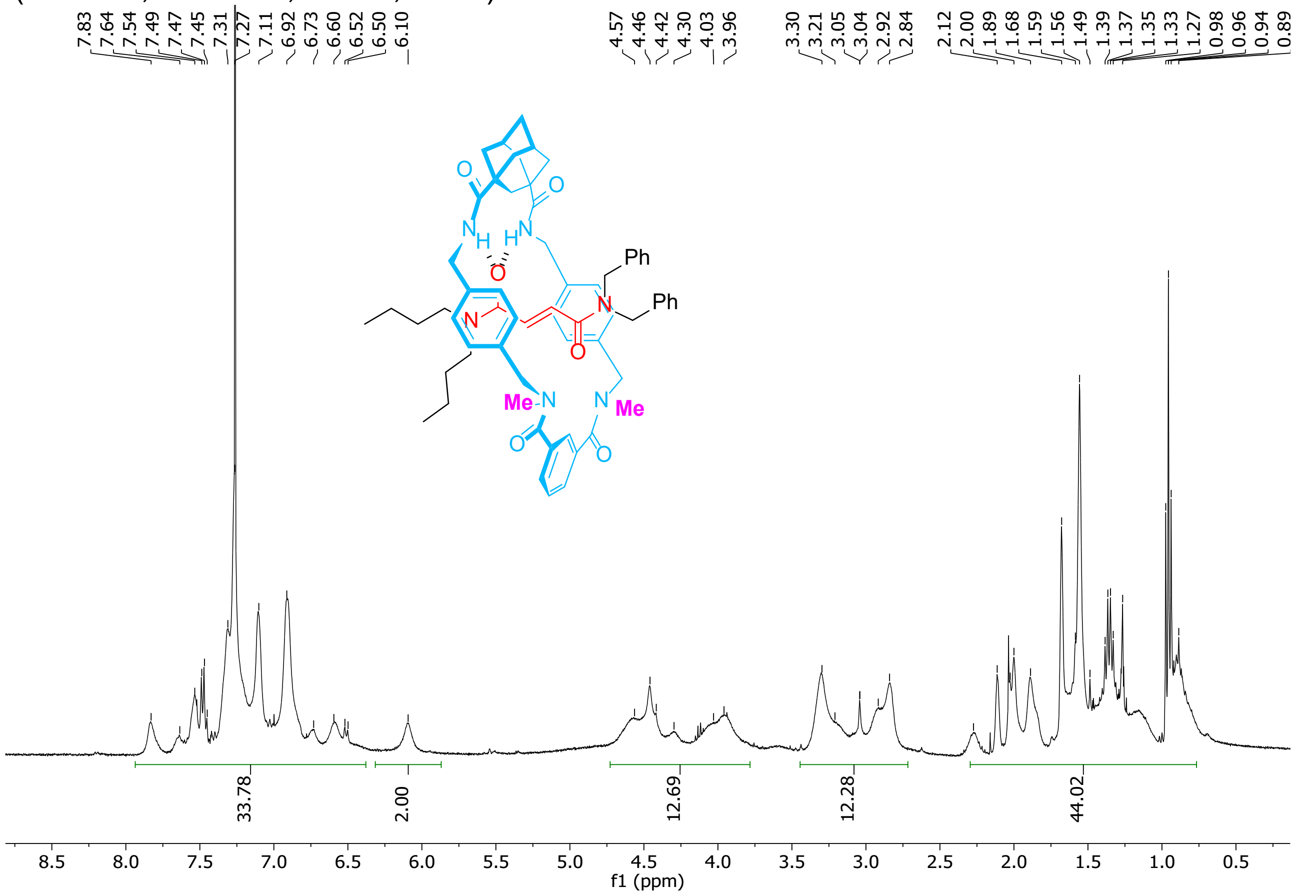

5b (^{13}C NMR, 101 MHz, $CDCl_3$, 318K)

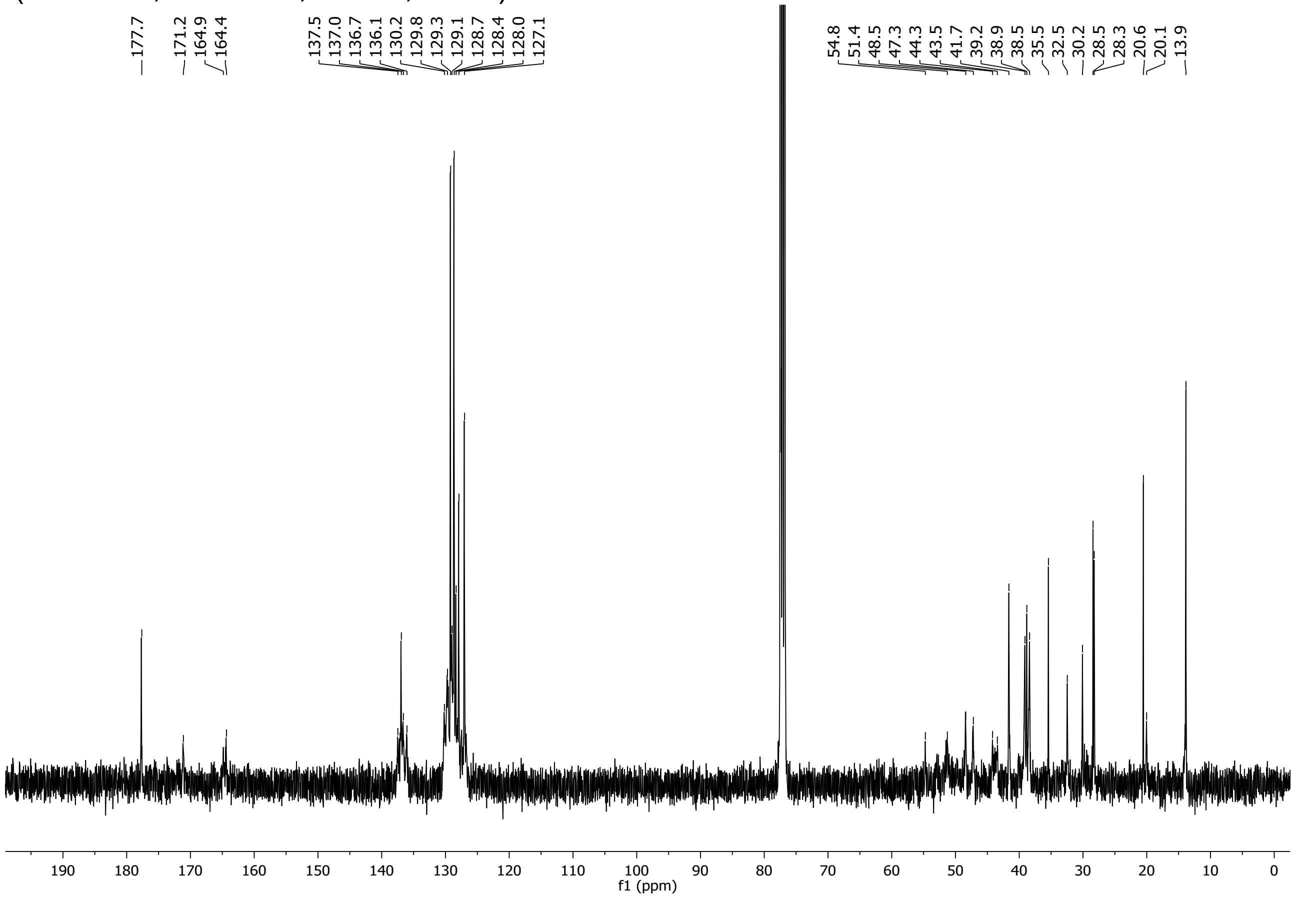

5b (^{135}DEPT NMR, 101 MHz, $CDCl_3$, 318K)

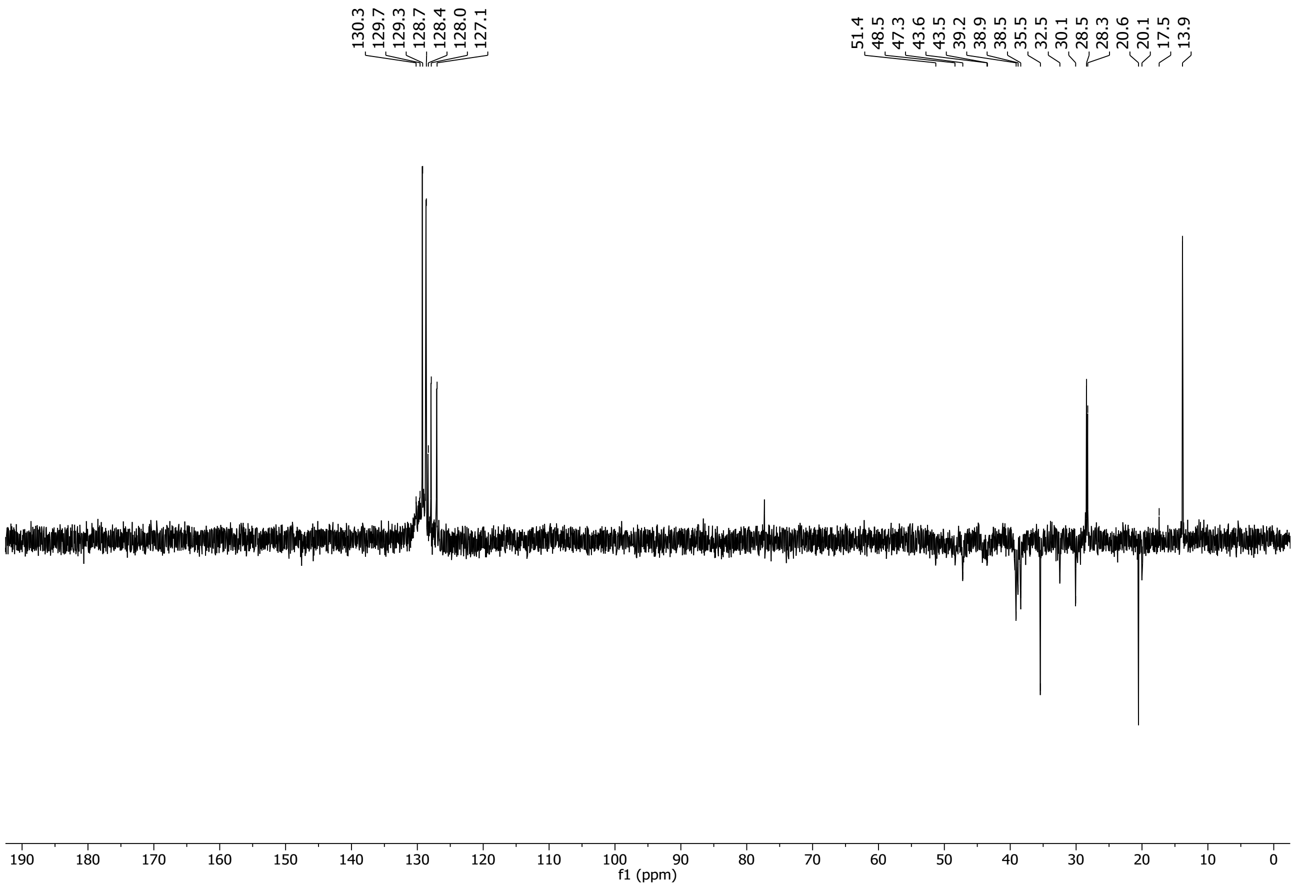

5b (COSY, 400 MHz, $CDCl_3$, 318K)

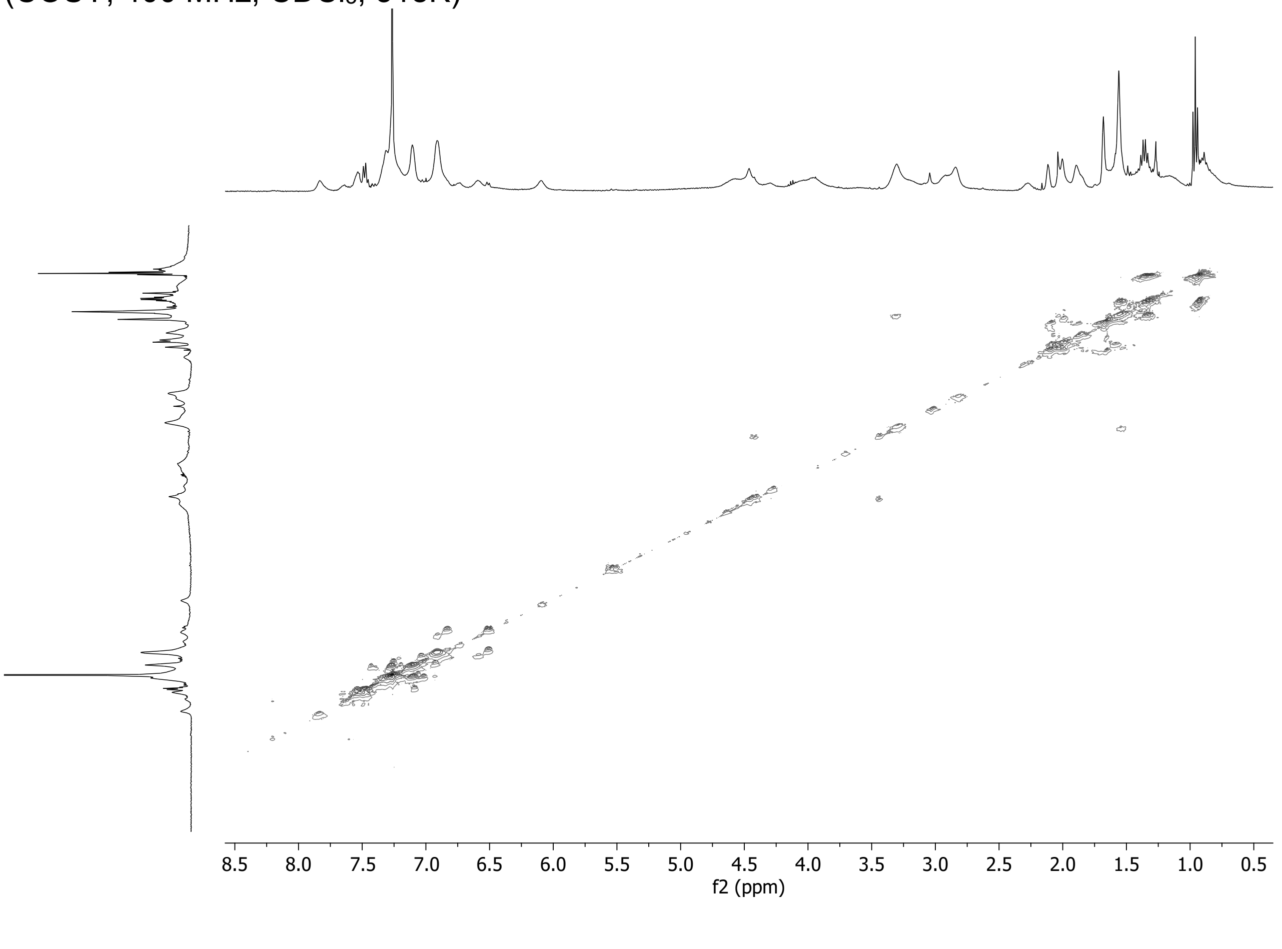

***rac*-7b** (^{1}H NMR, 400 MHz, $CDCl_3$, 318K)

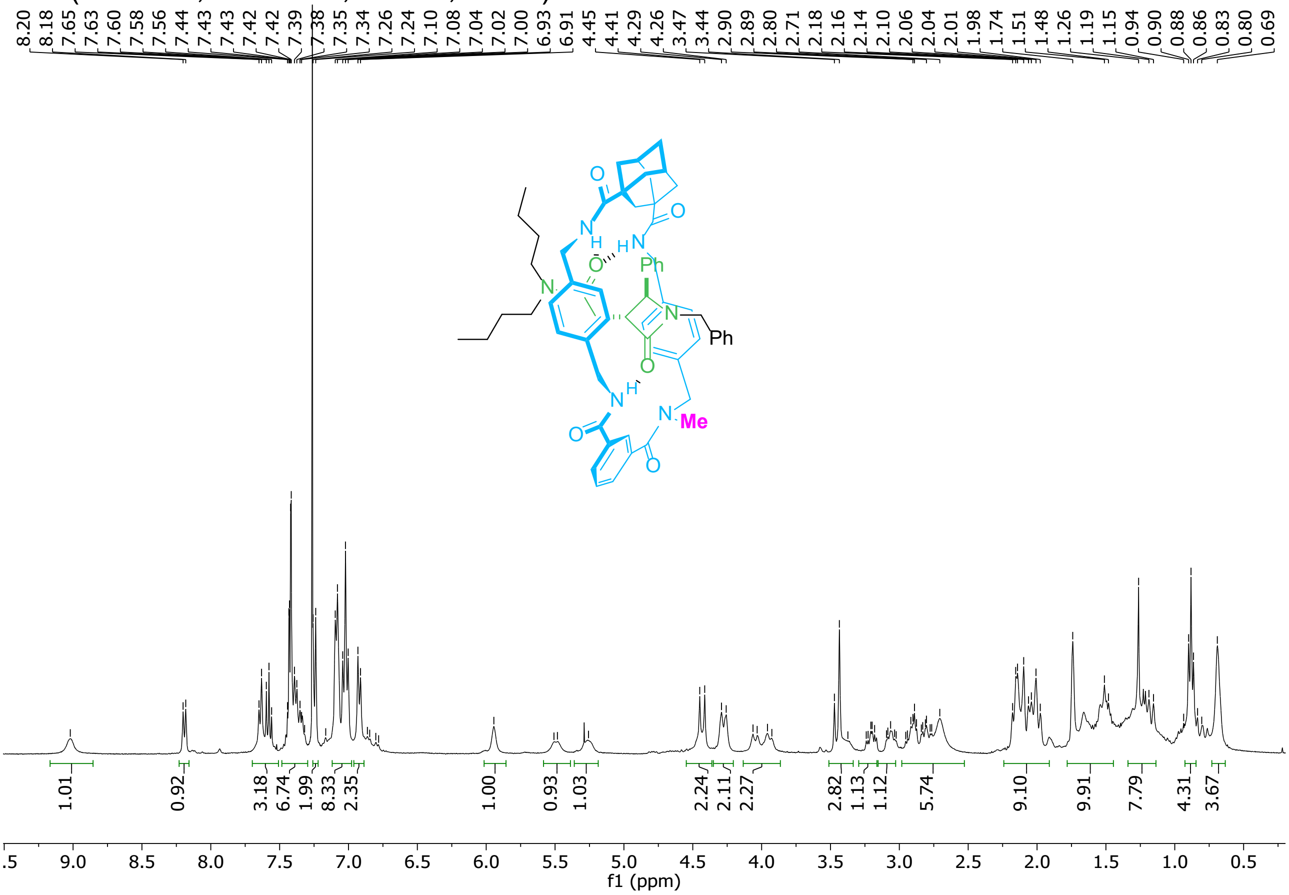

***rac*-7b** (^{13}C NMR, 101 MHz, $CDCl_3$, 318K)

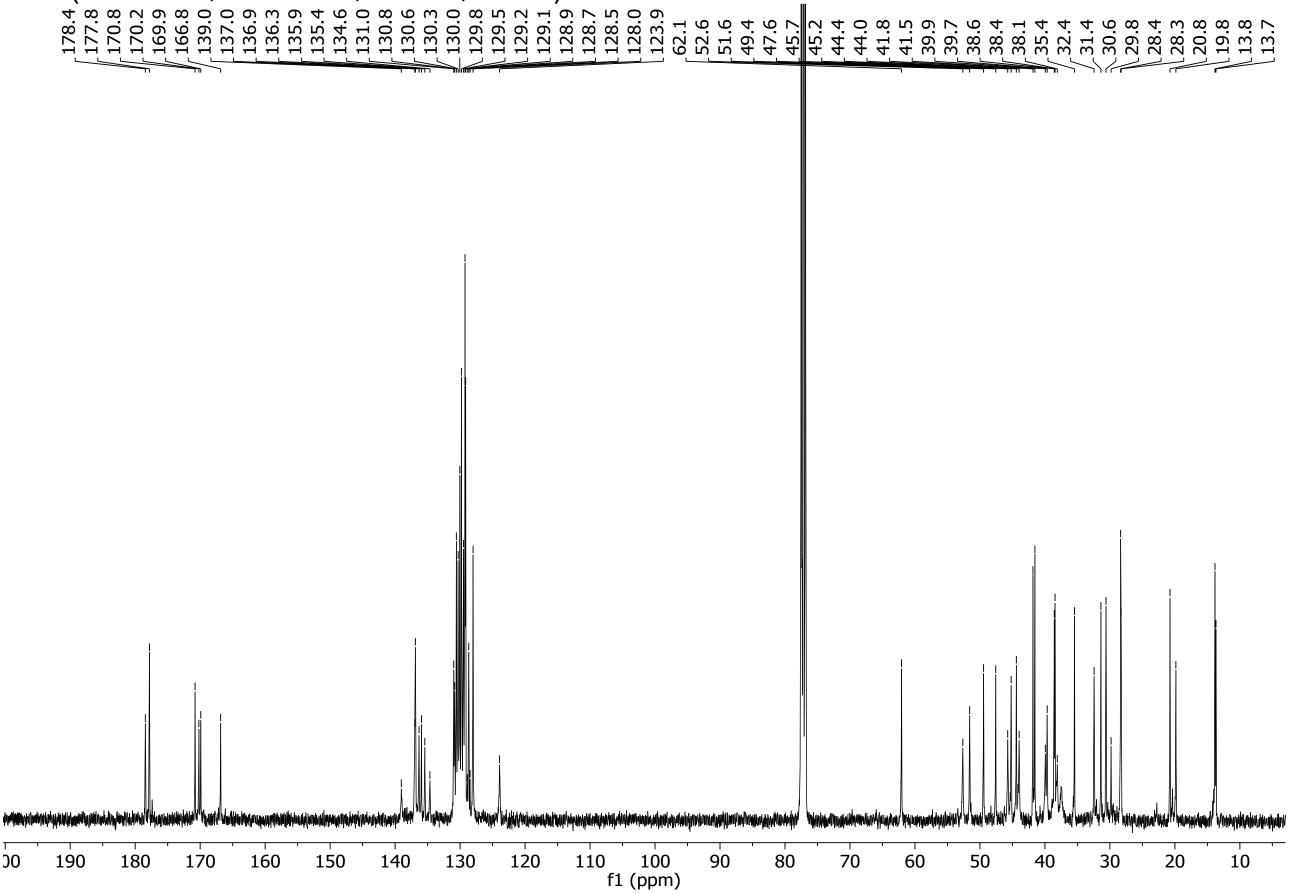

***rac*-7b** (^{135}DEPT NMR, 101 MHz, $CDCl_3$, 318K)

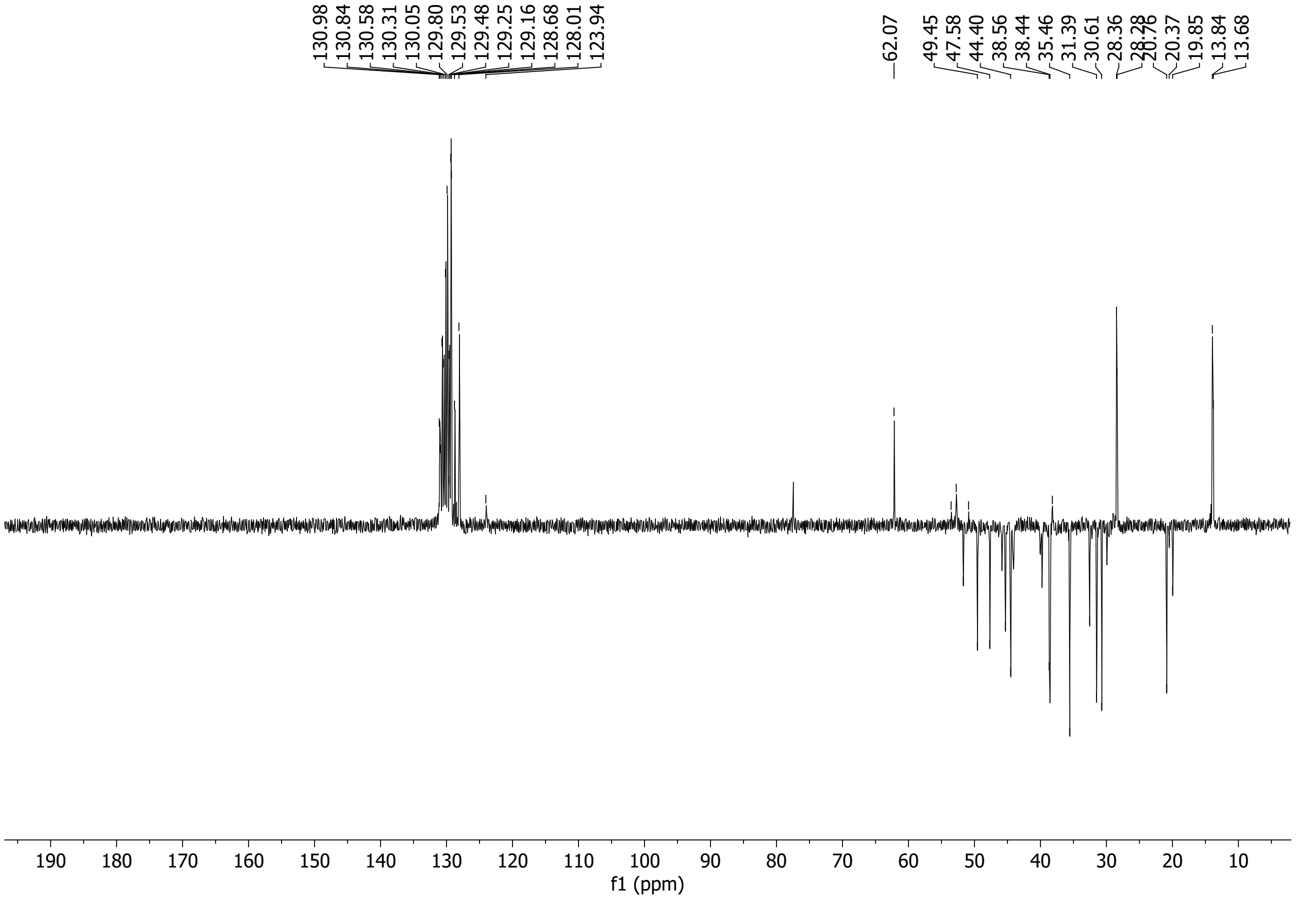

***rac*-7b** (COSY, 400 MHz, $CDCl_3$, 318K)

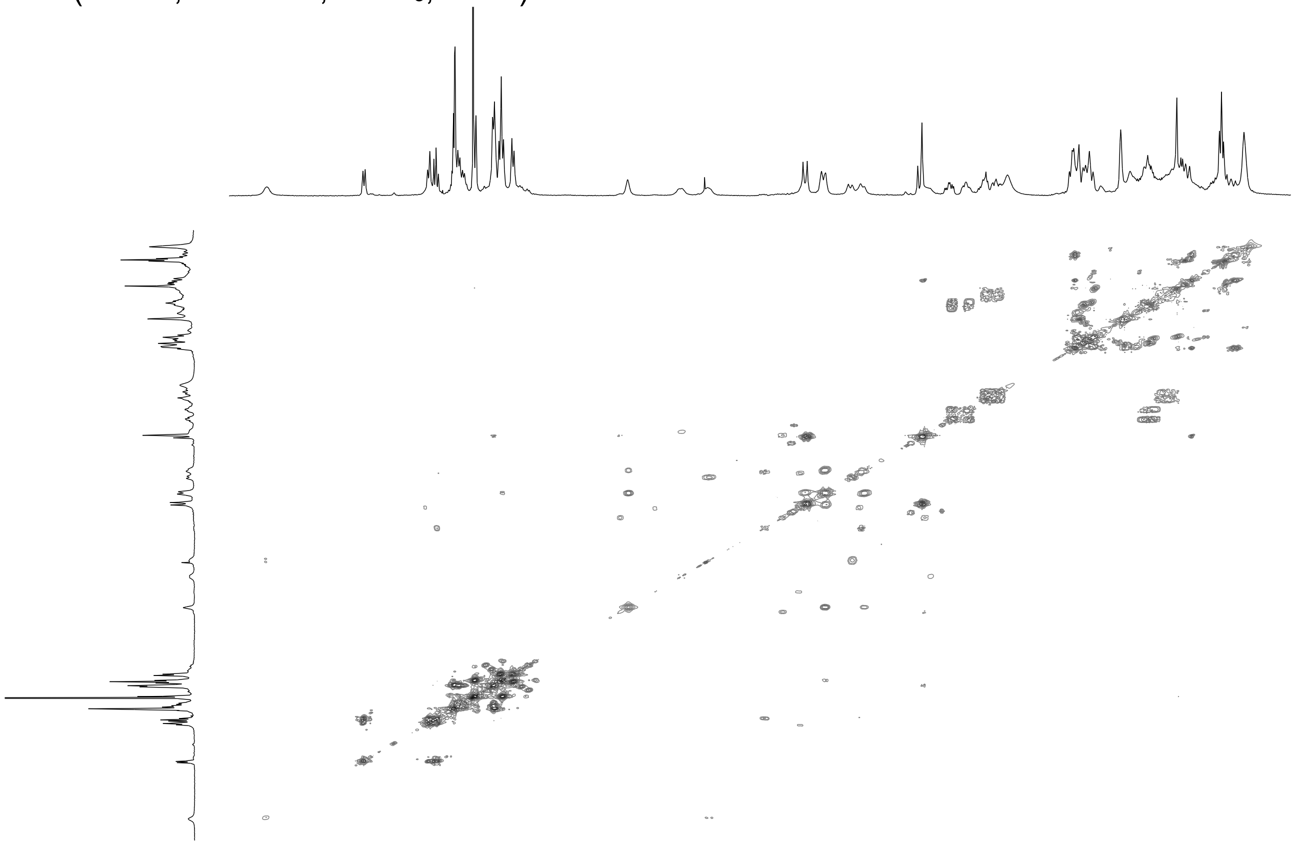

rac-7b (HMQC, $CDCl_3$, 318K)

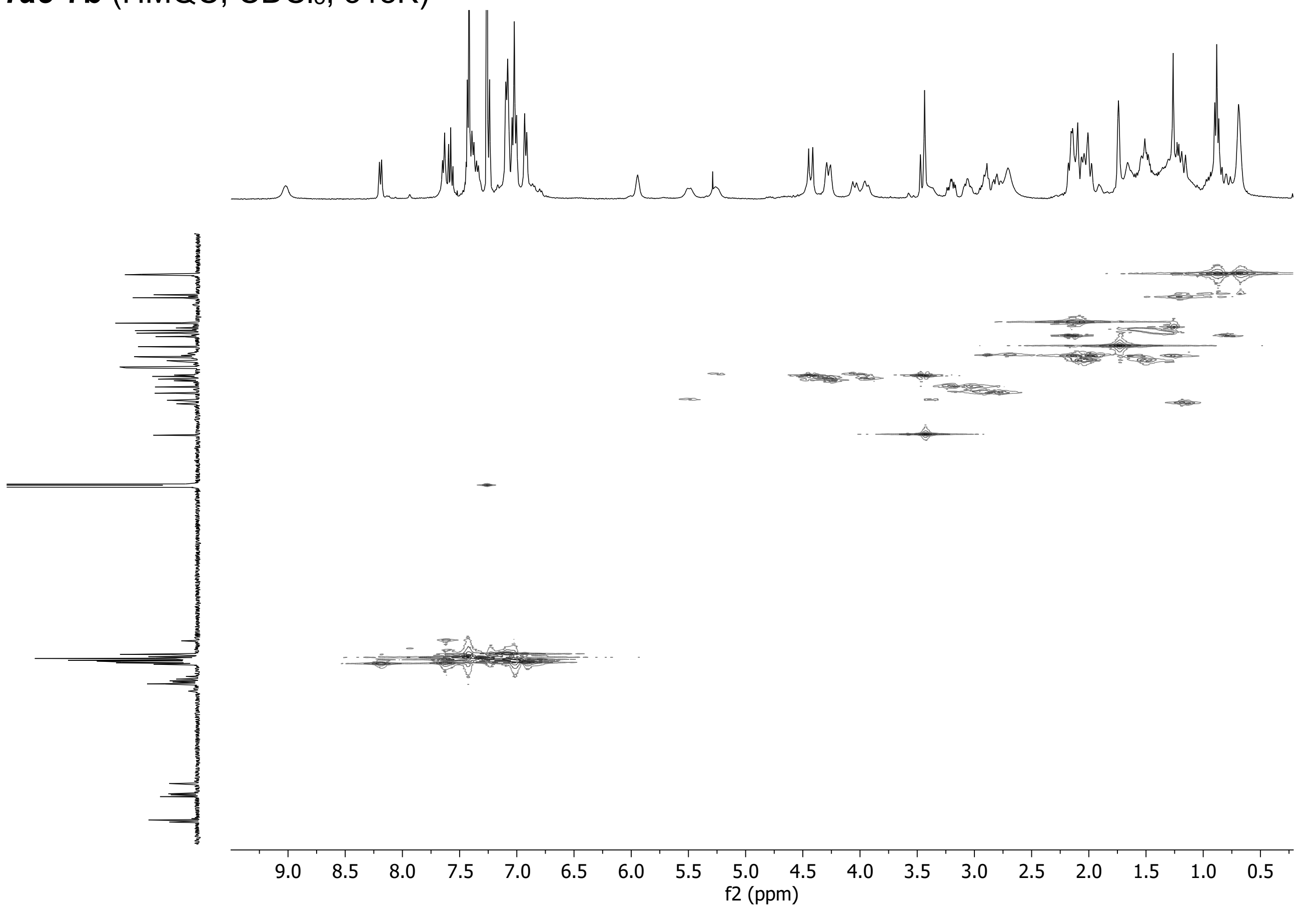

rac-7b (HMBC, $CDCl_3$, 318K)

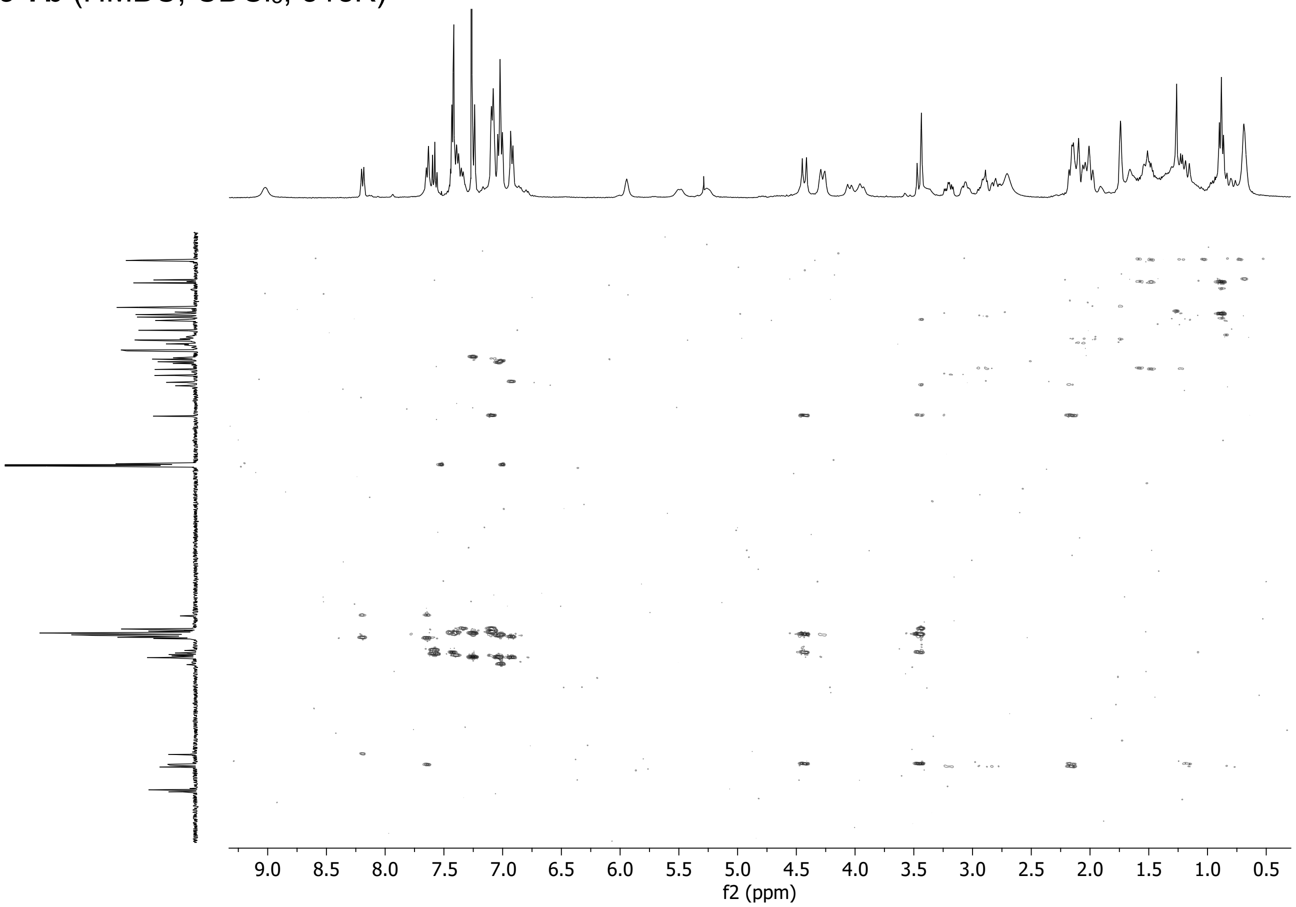

M1 (^{1}H NMR, 300 MHz, DMSO-d6, 353K)

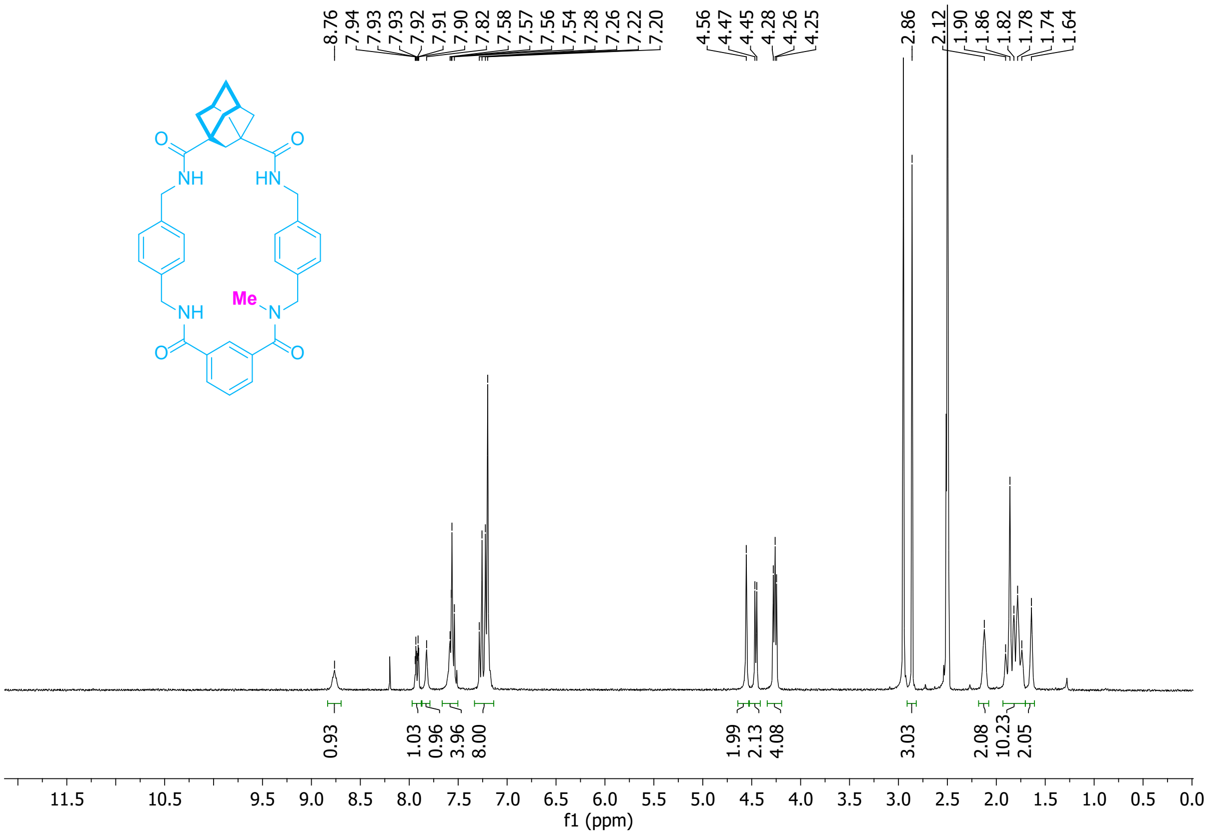

M1 (COSY, 300 MHz, DMSO-d6, 353K)

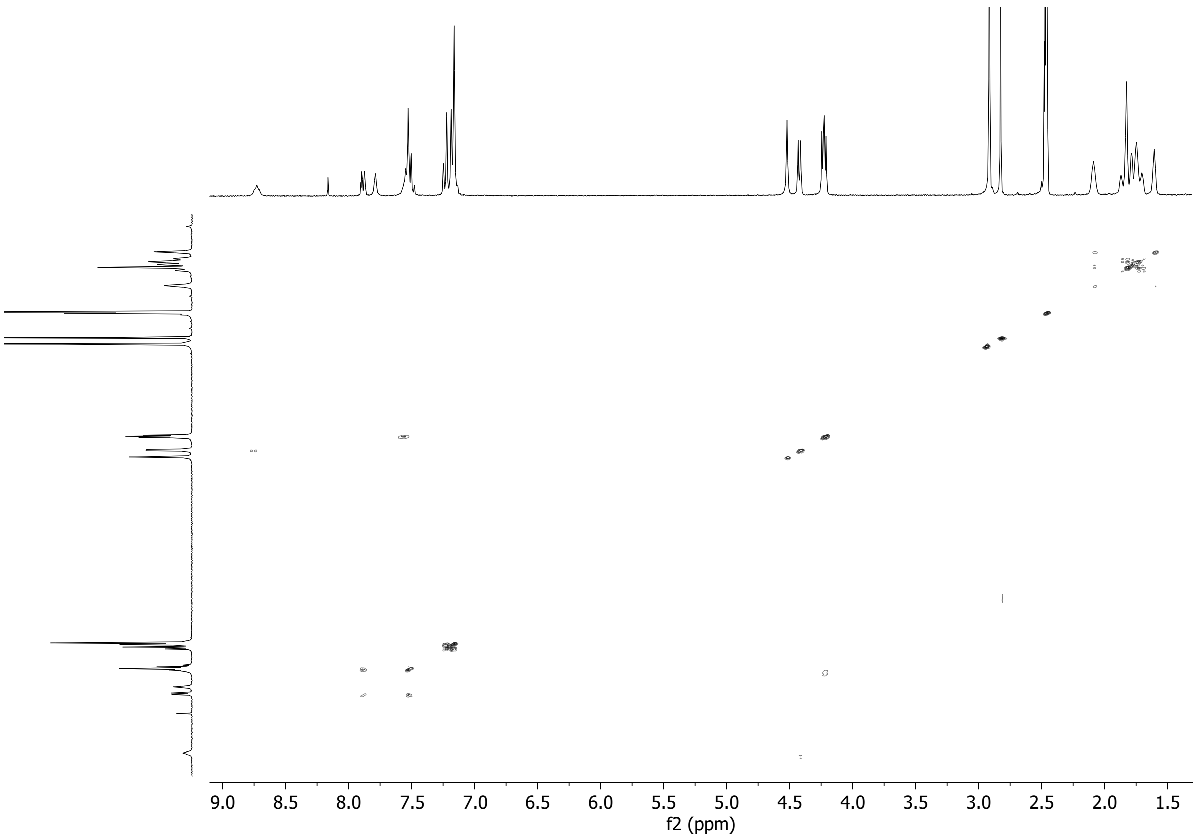

M1 (^{13}C NMR, 75 MHz, DMSO-d$_6$, 353K)

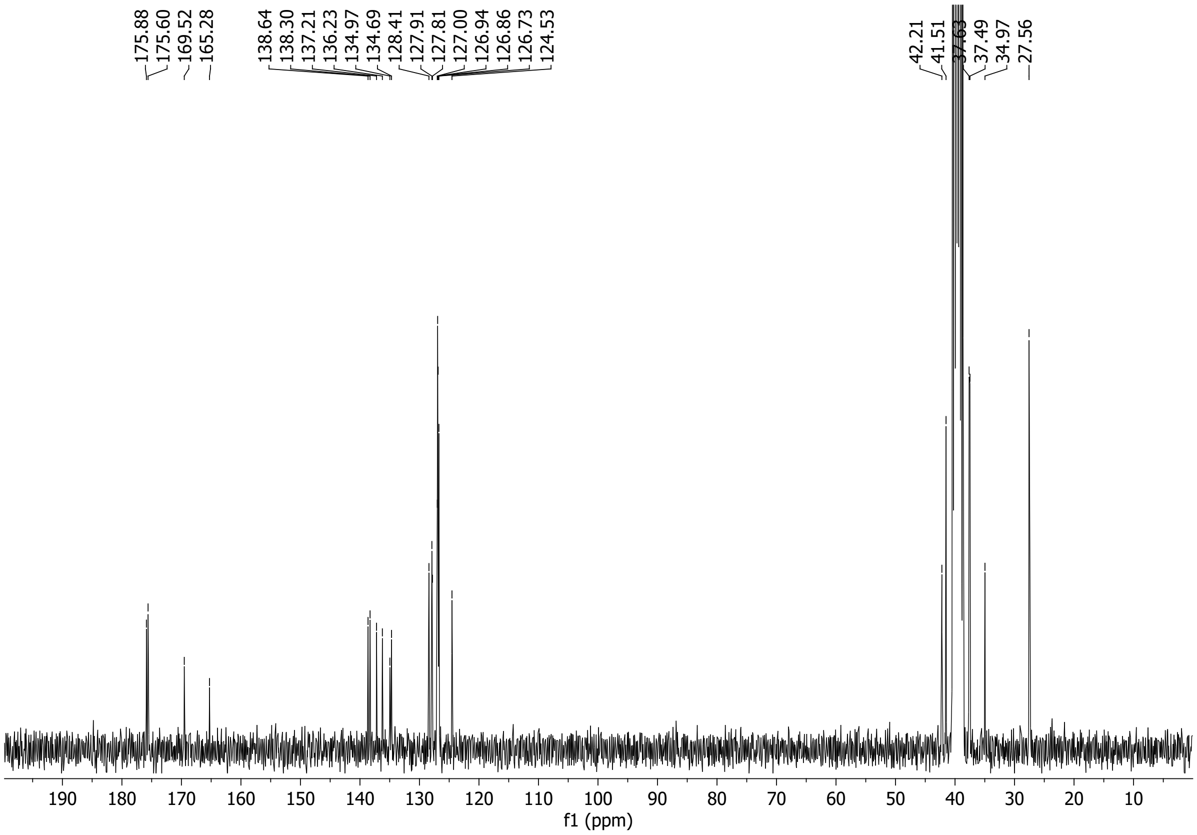

M1 (^{135}DEPT, 75 MHz, DMSO-d$_6$, 353K)

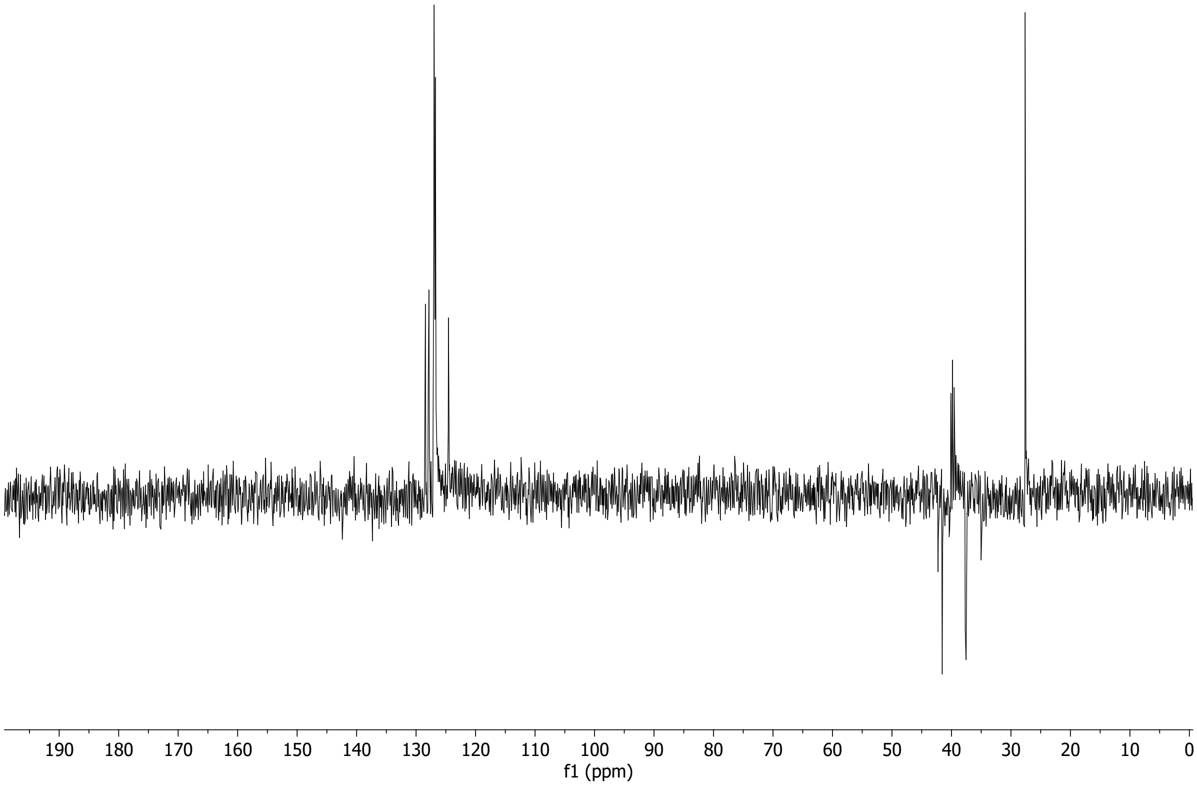

M1 (HMQC, DMSO-d6, 353K)

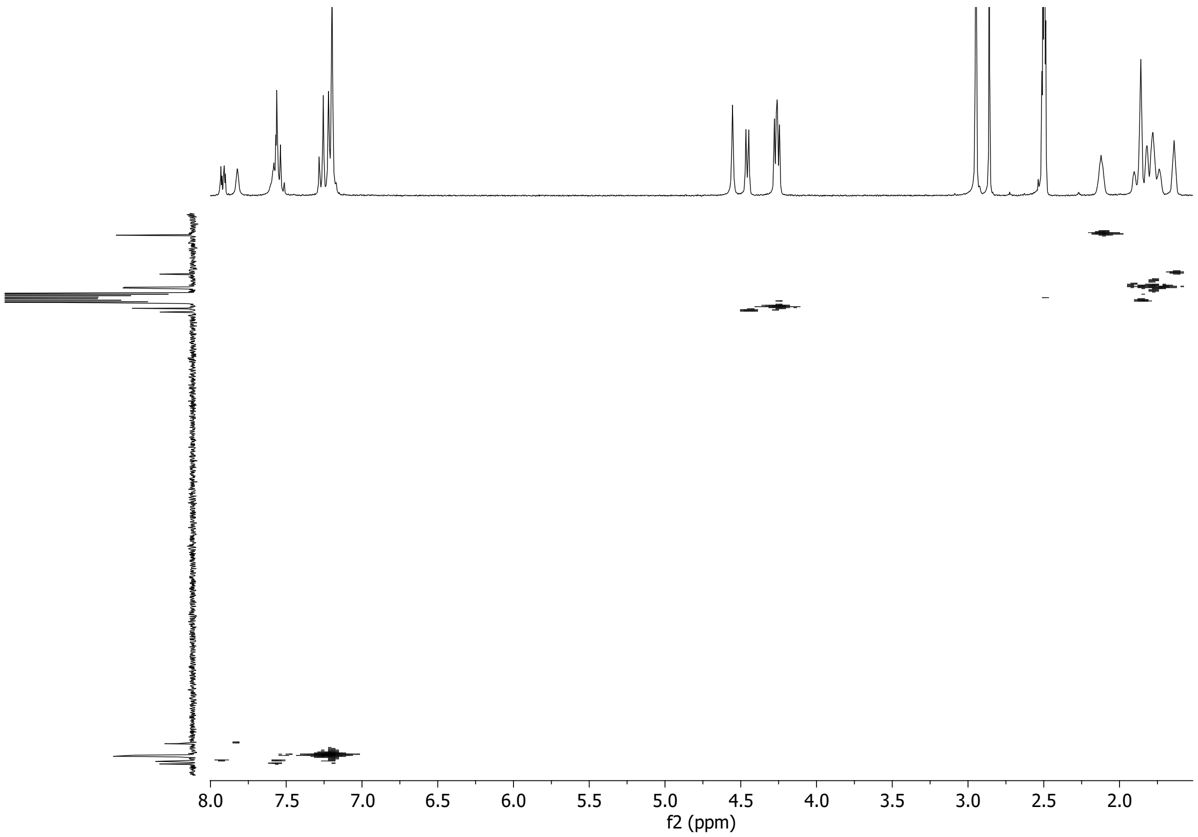

16. Copies of HPLC Chromatograms

Conditions: Hypersil Gold (silica, 5 μm) column, DCM: IPA: NH_4OH (30:0.25:0.1), 0.75 mL/min, 20°C, 280 nm

Rotaxane 4b

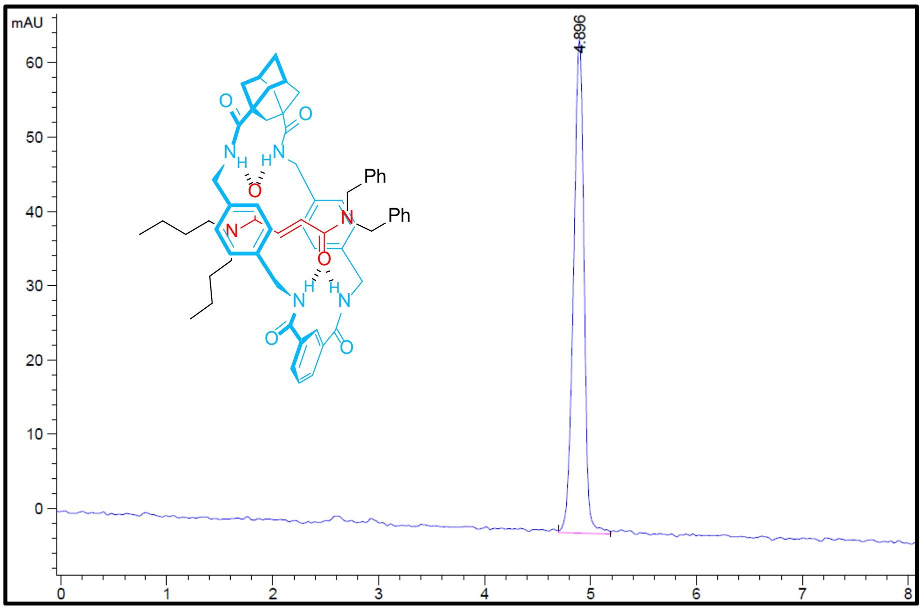

Rotaxane 1b

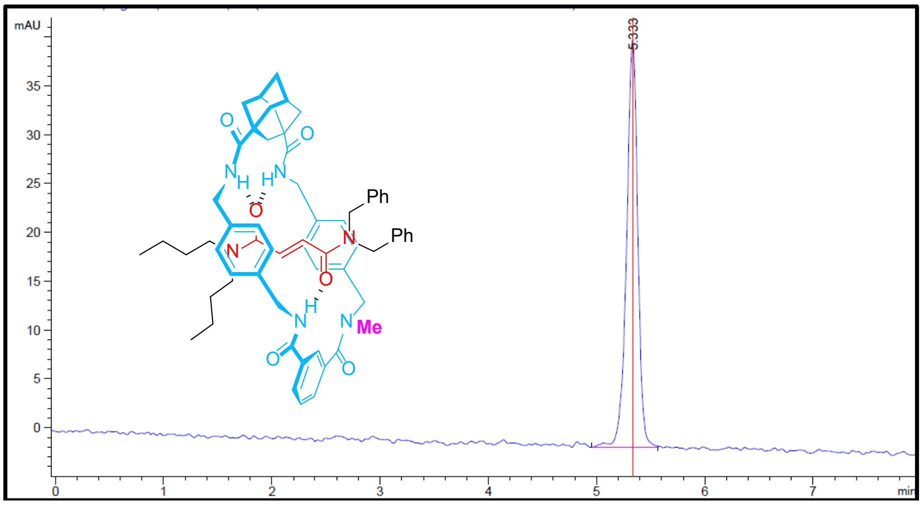

Rotaxane 5b

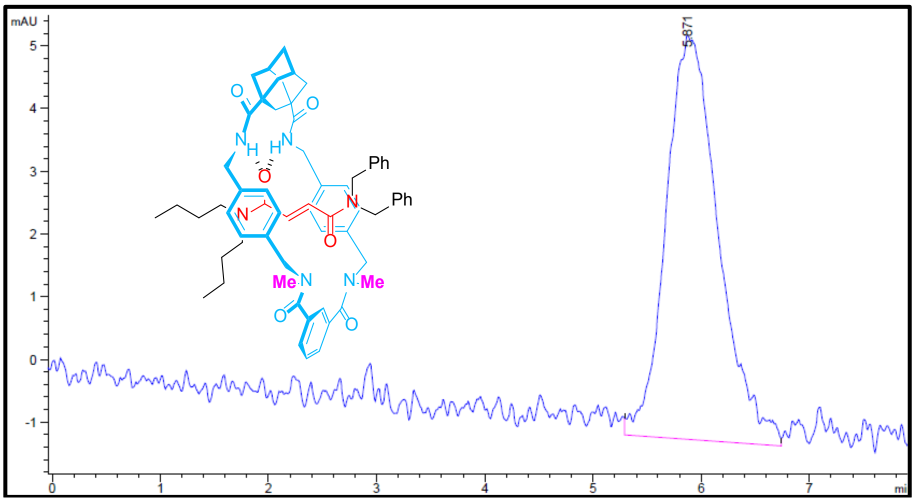

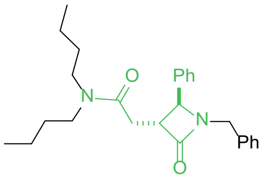

Conditions: Chiralpak IA-3 column, 97:3 Hex:IPA, 1 mL/min, T = 25 ºC, 210 nm

***rac*-2b**

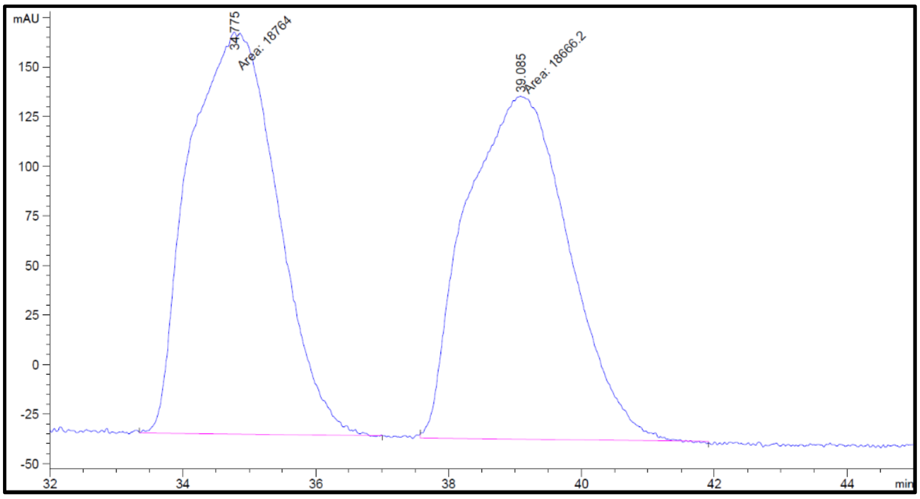

Peak #	RetTime [min]	Type	Width [min]	Area [mAU*s]	Height [mAU]	Area %
1	34.775	MM	1.5435	1.87640e4	202.60709	50.1306
2	39.085	MM	1.7976	1.86662e4	173.06734	49.8694

Reaction at 25 ºC from E1-1b

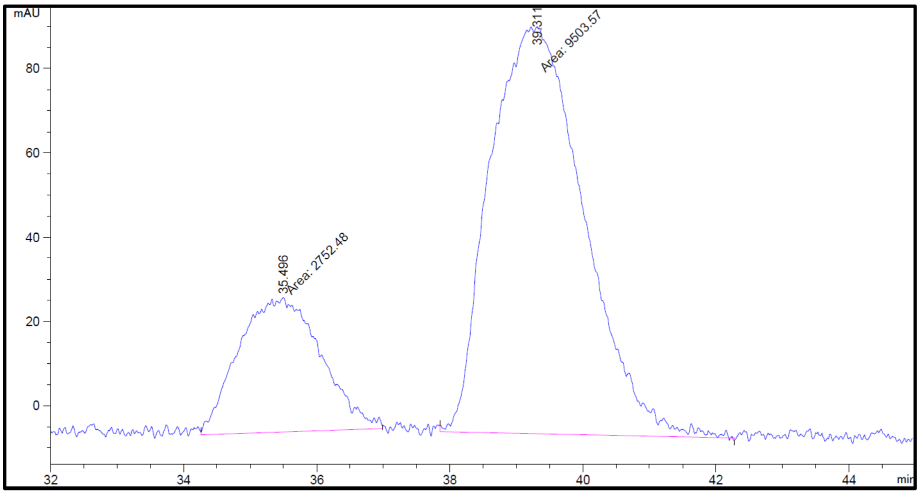

Peak #	RetTime [min]	Type	Width [min]	Area [mAU*s]	Height [mAU]	Area %
1	35.496	MM	1.4371	2752.47681	31.92138	22.4581
2	39.311	MM	1.6391	9503.56738	96.63568	77.5419

Reaction at - 20 ºC from E1-1b

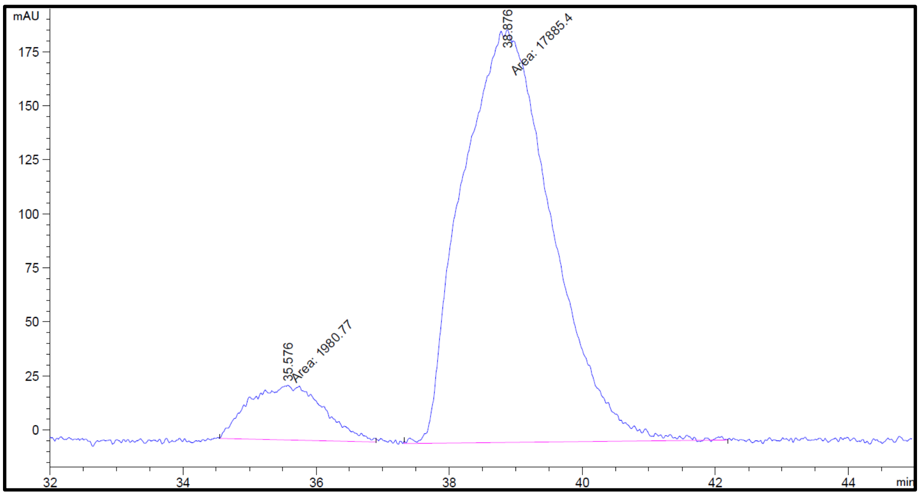

Peak #	RetTime [min]	Type	Width [min]	Area [mAU*s]	Height [mAU]	Area %
1	35.576	MM	1.3035	1980.77393	25.32708	9.9706
2	38.876	MM	1.5602	1.78854e4	191.05386	90.0294

Reaction at - 20 ºC from E2-1b

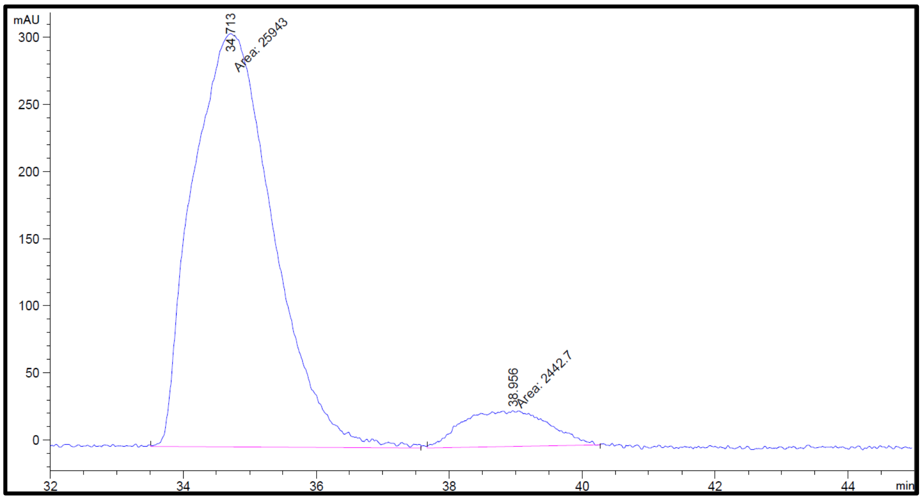

Peak #	RetTime [min]	Type	Width [min]	Area [mAU*s]	Height [mAU]	Area %
1	34.713	MM	1.4035	2.59430e4	308.07233	91.3946
2	38.956	MM	1.5192	2442.69653	26.79718	8.6054

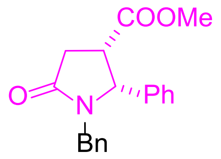

4

Conditions: Chiralpak IC-3 column, 70:30 Hex:IPA, 1 mL/min, T = 25 ºC, 210 nm

***rac*-3b**

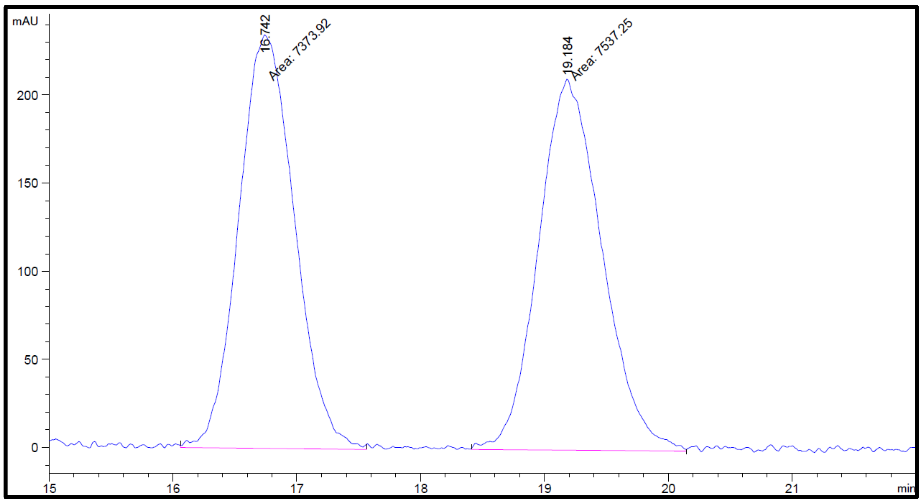

Peak #	RetTime [min]	Type	Width [min]	Area [mAU*s]	Height [mAU]	Area %
1	16.742	MM	0.5239	7373.91895	234.59293	49.4523
2	19.184	MM	0.5965	7537.25146	210.58682	50.5477

Reaction at - 20 ºC from E1-1b

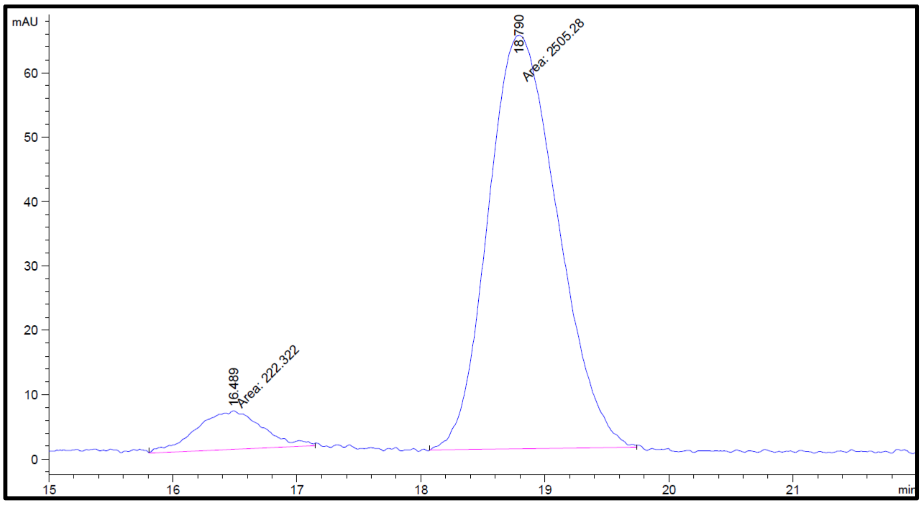

Peak #	RetTime [min]	Type	Width [min]	Area [mAU*s]	Height [mAU]	Area %
1	16.489	MM	0.6155	222.32243	6.01973	8.1508
2	18.790	MM	0.6504	2505.27881	64.20184	91.8492

Reaction at - 20 ºC from E1-1b

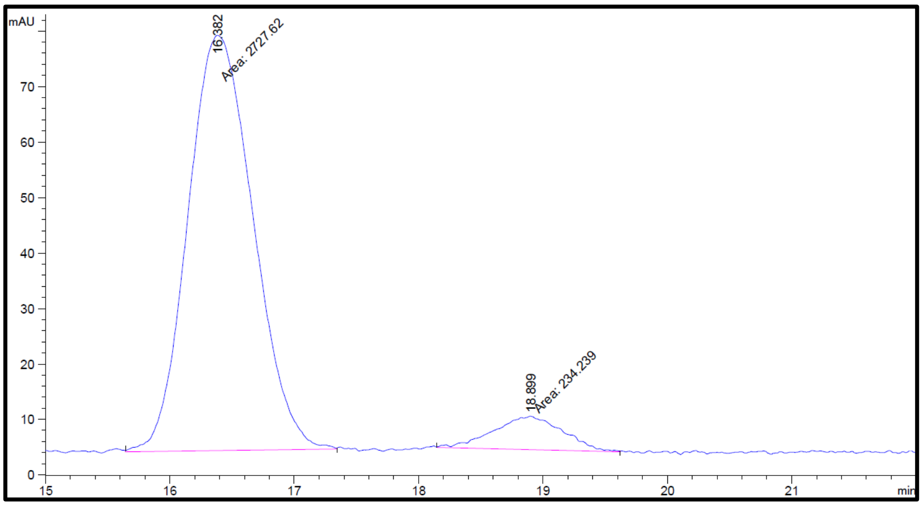

Peak #	RetTime [min]	Type	Width [min]	Area [mAU*s]	Height [mAU]	Area %
1	16.382	MM	0.6073	2727.62036	74.85676	92.0915
2	18.899	MM	0.6449	234.23883	6.05391	7.9085

17. References

[1] A. Martinez-Cuezva, C. Lopez-Leonardo, D. Bautista, M. Alajarin, J. Berna, *J. Am. Chem. Soc.*, **2016**, 138, 8726–8729.
[2] A. Martinez-Cuezva, F. Morales, G. R. Marley, A. Lopez-Lopez, J. C. Martinez-Costa, D. Bautista, M. Alajarin, J. Berna, *Eur. J. Org. Chem.* **2019**, 3480-3488.
[3] D. González Cabrera, B. D. Koivisto, D. A. Leigh, *Chem. Commun.* **2007**, 4218-4220.
[4] Its synthesis was carried out by following reported protocols: V. N. Wakchaure, B. List, *Angew. Chem. Int. Ed.* **2016**, *55*, 15775-15778.
[5] C. Lopez-Leonardo, A. Saura-Sanmartin, M. Marin-Luna, M. Alajarin, A. Martinez-Cuezva, J. Berna, *Angew. Chem. Int. Ed.* **2022**, *61*, e202209904.
[6] M. Swain, chemicalize.org. *J. Chem. Inf. Model.* **2012**, *52*, 613−615.
[7] O. V. Dolomanov, L. J. Bourhis, R. J. Gildea, J. A. K. Howard, H. Puschmann, *J. Appl. Cryst.* **2009**, *42*, 339-341.
[8] A. Altomare, G. Cascarano, C. Giacovazzo, A. Guagliardi, *J. Appl. Crystallogr.***1993**, *26*, 343.
[9] a) G. M. Sheldrick, F2 SHELXL-2014/7: Program for the Solution of Crystal Structures; University of Göttingen: Göttingen, Germany, **2014**. B) G. M. Sheldrick, Crystal structure refinement with SHELXL, *Acta Cryst.* **2015**, C71, 3-8.
[10] C. Lopez-Leonardo, A. Saura-Sanmartin, M. Marin-Luna, M. Alajarin, A. Martinez-Cuezva, J. Berna, *Angew. Chem. Int. Ed.* **2022**, *61*, e202209904.
[11] a) C. Reuter, A. Mohry, A. Sobanskiand, F. Vögtle, C*hem.Eur. J.*, **2000**, *6*, 1674; b) C. Reuter, C. Seel, M. Niegerand F. Vögtle, *Helv. Chim. Acta*., **2000**, *83*, 630.
[12] J.-D. Chai, M. Head-Gordon, M. Long-Range Corrected Hybrid Density Functionals with Damped Atom–Atom Dispersion Corrections. *Phys. Chem. Chem. Phys.* **2008**, *10*, 6615-6620.
[13] F. Weigend. Accurate Coulomb-Fitting Basis Sets for H to Rn. *Phys. Chem. Chem. Phys.* **2006**, *8*, 1057-1065.
[14] R. Bauernschmitt, R. Ahlrichs. Stability Analysis for Solutions of the Closed Shell Kohn–Sham Equation. *J. Chem. Phys.* **1996**, *104*, 9047–9052.
[15] S. Grimme, A. Hansen, S. Ehlert, J.M. Mewes. r2SCAN-3c: A "Swiss army knife" composite electronic-structure method. *J Chem Phys.* **2021**, *154*, 064103.
[16] A. V. Marenich, C. J. Cramer, D. G. Truhlar, Universal Solvation Model Based on Solute Electron Density and on a Continuum Model of the Solvent Defined by the Bulk Dielectric Constant and Atomic Surface Tensions. *J. Phys. Chem. B* **2009**, *113*, 6378–6396.
[17] Gaussian 16, Revision C.01, M. J. Frisch, G. W. Trucks, H. B. Schlegel, G. E. Scuseria et al. Gaussian, Inc., Wallingford CT, **2016**.
[18] F. Neese, Software update: The ORCA program system—Version 5.0. *WIREs Comput Mol Sci*. **2022**; 12:e1606.
